# Tetracene Diacid Aggregates for Directing Energy Flow toward Triplet Pairs

**DOI:** 10.1021/jacs.4c02058

**Published:** 2024-04-12

**Authors:** Nicholas F. Pompetti, Kori E. Smyser, Benjamin Feingold, Raythe Owens, Bimala Lama, Sandeep Sharma, Niels H. Damrauer, Justin C. Johnson

**Affiliations:** † National Renewable Energy Laboratory, 15013 Denver West Pkwy, Golden, Colorado 80401, United States; ‡ University of Colorado, Boulder, Colorado 80401, United States; § Renewable and Sustainable Energy Institute, University of Colorado Boulder, Boulder, Colorado 80401, United States

## Abstract

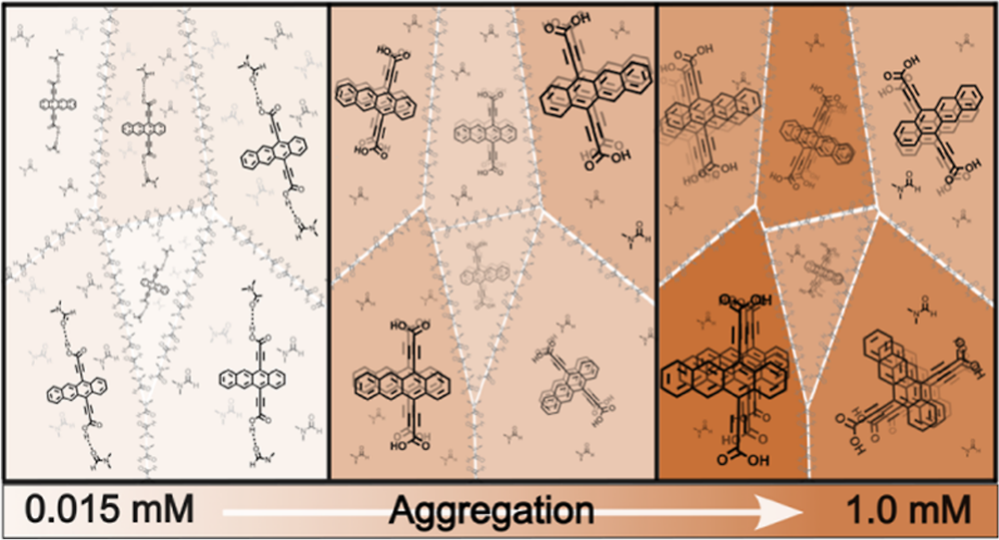

A comprehensive investigation of the solution-phase photophysics of tetracene bis-carboxylic acid [**5,12-tetracenepropiolic acid** (**Tc-DA**)] and its related methyl ester [**5,12-tetracenepropynoate** (**Tc-DE**)], a non-hydrogen-bonding counterpart, reveals the role of the carboxylic acid moiety in driving molecular aggregation and concomitant excited-state behavior. Low-concentration solutions of **Tc-DA** exhibit similar properties to the popular 5,12-bis((triisopropylsilyl)ethynl)tetracene, but as the concentration increases, evidence for aggregates that form excimers and a new mixed-state species with charge-transfer (CT) and correlated triplet pair (TT) character is revealed by transient absorption and fluorescence experiments. Aggregates of **Tc-DA** evolve further with concentration toward an additional phase that is dominated by the mixed CT/TT state which is the only state present in **Tc-DE** aggregates and can be modulated with the solvent polarity. Computational modeling finds that cofacial arrangement of **Tc-DA** and **Tc-DE** subunits is the most stable aggregate structure and this agrees with results from ^1^H NMR spectroscopy. The calculated spectra of these cofacial dimers replicate the observed broadening in ground-state absorption as well as accurately predict the formation of a near-UV transition associated with a CT between molecular subunits that is unique to the specific aggregate structure. Taken together, the results suggest that the hydrogen bonding between **Tc-DA** molecules and the associated disruption of hydrogen bonding with solvent produce a regime of dimer-like behavior, absent in **Tc-DE**, that favors excimers rather than CT/TT mixed states. The control of aggregate size and structure using distinct functional groups, solute concentration, and solvent in tetracene promises new avenues for its use in light-harvesting schemes.

## Introduction

Molecular aggregates, defined as the complexation of two or more chromophores, exhibit photophysical, electronic, and chemical properties not available to the isolated monomer on its own.^1^ As functional units within energy/charge-transfer (CT) architectures, aggregates are of particular interest for cutting-edge biomedical (biological imaging through aggregate-induced emission), solar-energy-harvesting (singlet fission, upconversion, and CT), and light-generating technologies (organic diodes).^2−4^ Emergent properties underlying these functions are highly sensitive to the arrangement of molecules within the aggregate structure and can be further tuned through chemical functionalization or environmental factors such as solvent, temperature, or pressure. For example, perylene-based aggregates coerced to organize with orthogonally oriented transition dipoles are expected to effectively filter charges via transport differences between electrons and holes, which could lead to a highly charge-carrier-selective and anisotropic optoelectronic material.^5^ Furthermore, tailored H-aggregate structures of 4,6-diphenyl-2-carbazolyl-1,3,5-triazine significantly enhance the triplet excited-state lifetime, enabling luminescence lifetimes up to 1 s.^6^

The simplest aggregate is a dimer consisting of two molecular subunits that interact with one another via noncovalent interactions (hydrogen bonding, π-anion, and π–π interactions). The strength of these intermolecular interactions and the solvent environment determine the energetic driving force and propensity toward stable and deterministic aggregation. Weak intermolecular interactions allow molecules to dissociate from one another and act as monomer units, precluding the possibility of aggregate-derived emergent behaviors. When interactions are strong and not designed to lead to specific structures, large and uncontrolled aggregates are more likely to form, sometimes compromising solubility. This leaves an opportunity and a challenge for synthetic chemists to design chromophores with intrinsic functionalities that balance through-space interactions and produce a desired result.

Tetracene (Tc) and its derivatives have been well-studied in a variety of fields relating to optoelectronic applications. Their dimers and higher-order oligomers have become ubiquitous elements of singlet fission (SF) investigations.^7,8^ SF and its inverse process, triplet–triplet annihilation-based upconversion (TTA–UC), rigorously require at least two chromophores to interact while the system carries an excited electronic state. To reveal interchromophore interactions that optimize the respective processes, the fields of SF and TTA–UC typically employ two classes of molecular assemblies—those that form in solutions and those in the solid state. In solution, covalent bridges are conveniently used to bond chromophores at different distances and relative orientations while maintaining contact with an adaptable dielectric environment, though, except for a few examples, most covalent dimers have flexible components yielding many degrees of freedom, often obscuring true structure–function relationships.^9−12^ In contrast, films and crystals of SF molecules have well-defined and rigid intermolecular geometries driven by electrostatic or van der Waals forces. However, crystallization can be difficult to achieve for some molecular systems, and inhomogeneity and lack of contact with an adaptable dielectric environment are detrimental to deriving a fundamental understanding, particularly regarding the role of CT states. Nanoscale solid structures, often termed molecular nanoparticles, can be soluble, making them convenient for solution-phase spectroscopic studies. However, their surfaces typically require some functionalization to produce colloidal stability, and their degree of crystallinity is difficult to discern.^13,14^ The heterogeneous molecular environment complicates analysis. Small aggregates, if readily produced with well-defined structures, possess the benefits of rigid geometries without the constraint of complicated synthesis or long-range crystallization and retain controlled adaptability through external and environmental perturbations. In the context of photophysical or photochemical transformations, requiring diffusion-controlled encounters between reactants can limit efficiency when excited-state lifetimes are short, and the preassociation of molecules in stable aggregates is potentially advantageous.^15,16^

**Figure 1:**
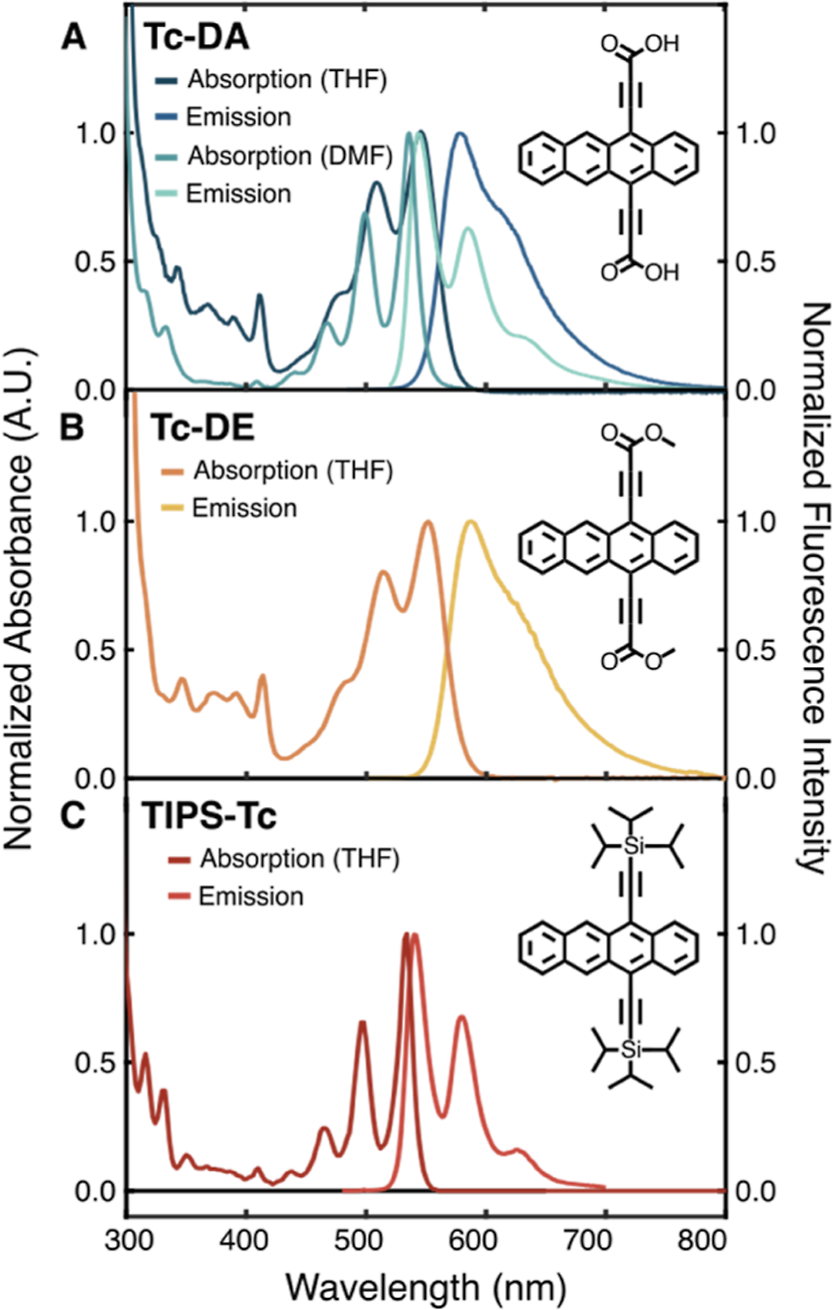
Steady-state absorption and emission spectra of 20 μM (A) **Tc-DA**, (B) **Tc-DE**, and (C) **TIPS-Tc** in THF. Panel (A) demonstrates solvent dependence on the absorbance and emission spectral profile of **Tc-DA** between THF (blue) and dimethylformamide (DMF) (green).

Here we report a newly synthesized tetracene diacid, **5,12-tetracenepropiolic acid** (**Tc-DA**), Figure 1A, designed for chemical tethering to semiconductor surfaces through functionalization along the tetracene short axis (peri position). Substitution along this axis has been understudied compared to pro-cata derivatization of tetracene (and pentacene) for the same purpose.^17−20^ A dimethyl ester analogue, **5,12-tetracenepropynoate** (**Tc-DE**), Figure 1B, was also synthesized to provide isomorphic comparison but without the potential for chemical tethering and intermolecular hydrogen bonding. The steady-state and time-resolved solution-phase optical properties of these derivatives are investigated, revealing a strong concentration dependence of the optical behavior of **Tc-DA** dissolved in DMF. Through electronic structure calculations, we assign an emergent absorption band to be a transition associated with through-space intermolecular CT between the **Tc-DA** or **Tc-DE** subunits. An argument for the likely aggregate structure is made by the use of ^1^H NMR spectroscopy, computational modeling, and concentration-dependent optical behavior in the framework of exitonic coupling. By systematic control and comprehensive analysis of well-defined tetracene aggregates, we develop insights into the types of intermolecular interactions that likely lead to their emergent photophysical behavior, including SF.

## Results

### Synthesis

The synthesis of **Tc-DA** proceeds in a single step from 5,12-bis((trimethylsilyl)ethynyl-tetracene) (**TMS-Tc**), detailed in the Supporting Information (Scheme S1). This synthetic route is an extension of the desilylation chemistry developed by Yonemoto-Kobayashi et al. whereby **TMS-Tc** was exposed to cesium fluoride in the presence of carbon dioxide.^21^ Fluoride, in this case, acts as an efficient desilylating reagent of the trimethylsilyl group, and the resulting acetylide intermediate can undergo nucleophilic addition to CO_2_ dissolved in solution to afford a 5,12-carboxylate-capped ethynyl tetracene derivative (**Tc-DA**^**2–**^). The bis-carboxylate can be protonated in solution through aqueous workup to form the bis-carboxylic acid (**Tc-DA**, Figure 1A) or trapped with methyl iodide to form the bis-methyl ester (**Tc-DE**, Figure 1B). These reactions are particularly well-suited for the synthesis of symmetrically derivatized tetracene chromophores because the reaction can be performed at room temperature, is completed in 3 h under a CO_2_ atmosphere, and utilizes the resulting acetylide anion as an effective nucleophile.

**Tc-DE** was synthesized as a strategy to prevent intermolecular hydrogen bonding between tetracene subunits in solution while also preserving the core electronics of the **Tc-DA** molecule. As expected, **Tc-DE** proved to be more readily soluble than its diacid counterpart and can access a range of solvents convenient for spectroscopic characterization. Interestingly, both derivatives exist as dark blue amorphous solids but form a bright red solution upon reconstitution within an appropriate solvent. The drastic change in color from the solid state to solvated in solution is not typical of tetracene derivatives and hints at complex interactions between chromophores within the aggregated state.

### Steady-State Spectroscopy

Electronic absorption data were collected at room temperature in THF for both **Tc-DA** (Figure 1A) and **Tc-DE** (Figure 1B) at 20 μM and are shown in comparison to those of the well-studied 5,12-bis((triisopropylsilyl)ethynyl-tetracene (**TIPS-Tc**) (Figure 1C). All three tetracene derivatives exhibit a vibronic progression associated with the lowest-energy S_1_ ← S_0_ transition referred to as the ^1^L_a_ band in Platt notation.^22^ The onset of this transition is at 534, 550, and 552 nm for **TIPS-Tc**, **Tc-DA**, and **Tc-DE**, respectively. The relative heights of 0–0* and 0–1* within the ^1^L_a_ band, expressed as *A*_0–0_/*A*_0–1_, are 1.51 for **TIPS-Tc** but 1.25 for both **Tc-DA** and **Tc-DE**. Continuing to higher-energy wavelengths, the most intense absorption feature observed for all three derivatives (Figure S2) is referred to as the B band in Platt notation and is analogous to the S_3_ ← S_0_ transition in tetracene. This transition is centered at 290 nm for **TIPS-Tc** and slightly red-shifted to 294 nm for both **Tc-DE** and **Tc-DA**.

In THF, through a range of 0.006–1.00 mM, both **Tc-DA** and **Tc-DE** exhibit concentration-independent absorption behavior demonstrated through normalization of their lowest absorption peak (Figures S3 and S6). The molar absorption coefficients of these features are 10,700 and 11,700 M^–1^ cm^–1^, respectively. **TIPS-Tc** also demonstrates a concentration-independent optical absorption profile, owing to the solubilizing TIPS groups, but with a much larger 0–0* molar attenuation coefficient of 35,800 M^–1^ cm^–1^ (Figure S1). Broadening of the absorption profile appears to be unique to **Tc-DA** and **Tc-DE** in THF with respect to **TIPS-Tc** but also to previously studied carboxylated tetracene derivatives, such as 6,11-bis((triisopropylsilyl)ethynyl)tetracene-2-carboxylic acid, 5-tetracene-carboxylic acid, and others.^15,17,18^

The emission profiles for **Tc-DA** and **Tc-DE** in THF lack mirror image symmetry of the absorption spectrum, a behavior not typically found in other tetracene derivatives. Instead, **Tc-DA** and **Tc-DE** exhibit a significantly broadened emission with a Stokes shift of approximately 130 and 140 meV, respectively (Figure 1A,B). The fluorescence quantum yield (QY) in THF was measured to be 57% for **Tc-DA** and 51% for **Tc-DE**, with fluorescence lifetimes of 17.4 and 11.2 ns, respectively (Figures S15 and S16). For comparison, **TIPS-Tc** has an emission profile that mirrors its absorbance in THF (Figure 1C) with a small Stokes shift of 30 meV, a QY of 74%, and a 12.5 ns fluorescence lifetime (Figure S14). From Figure 1, the observed S_1_ energy via intersection of the absorption and emission profile is calculated to be 2.31, 2.21, and 2.18 eV for **TIPS-Tc**, **Tc-DA**, and **Tc-DE**, respectively.

**Tc-DA** and **Tc-DE** are further distinguished from **TIPS-Tc** by exhibiting a stronger absorption band from 370 to 410 nm than **TIPS-Tc** in THF. The band lies where S_2_ ← S_0_ (^1^L_b_) transitions might be for tetracene. This transition is described to be long-axis-polarized within the highly symmetric parent tetracene structure of D_2*h*_ symmetry where it is predicted to be nearly forbidden due to the nature of the out-of-phase combination of the frontier molecular orbitals (HOMO – 1 → LUMO and HOMO → LUMO + 1).^23^ Within the literature, the appearance of this band in tetracene derivatives seems to rely on the breaking of molecular symmetry through addition of electron-withdrawing groups to the tetracene core or through cofacial, long axis intermolecular interactions.^24,25^ The appearance of this feature within **Tc-DA** and **Tc-DE** is surprising because the overall molecular symmetry of the tetracene chromophore has not been reduced compared to **TIPS-Tc** (both of C_2*v*_ symmetry).

Interestingly, when the solvent is changed from THF to a more polar, aprotic solvent such as DMF, we observe a strikingly different absorption profile for **Tc-DA** (Figure 1A, green) but only minor changes to the absorption spectrum for **Tc-DE** (Figure S8). For **Tc-DA** in DMF at 20 μM, the ^1^L_a_ band is blue-shifted by 50 meV and displays improved resolution between vibronic transitions, nearly resembling the absorption profile of **TIPS-Tc** (Figure 1C). Furthermore, the putative, prominent ^1^L_b_ band has lost almost all oscillator strength, resembling a nearly forbidden transition predicted for a C_2*v*_ symmetric tetracene molecule. A further discussion on the nature of this band and its solvent-dependent appearance will be developed below.

Congruent with the absorption, the emission profiles of **Tc-DA** and **Tc-DE** also show solvent-dependent behavior. At 15 μM in DMF, the fluorescence spectrum of **Tc-DA** is a near mirror image of its absorption profile (Figure 1A, green) with a significantly reduced Stokes shift of 40 meV, a QY of 62%, and a fluorescence lifetime of 13.8 ns (Figure S19). The behavior of **Tc-DE** in DMF is distinct (Figure S8), as the emission red shifts resulting in an increased Stokes shift of 220 meV with a corresponding change in QY (51 to 43%) and fluorescence lifetime (11.2 to 15.9 ns, Figures S16 and S25). The observed solvent dependence on the steady-state behavior of **Tc-DA** and **Tc-DE** (Figures S8–S12) hints at an excited state influenced by the solvent polarity. This behavior is further complicated in the case of **Tc-DA**, which displays a concentration dependence.

**Figure 2:**
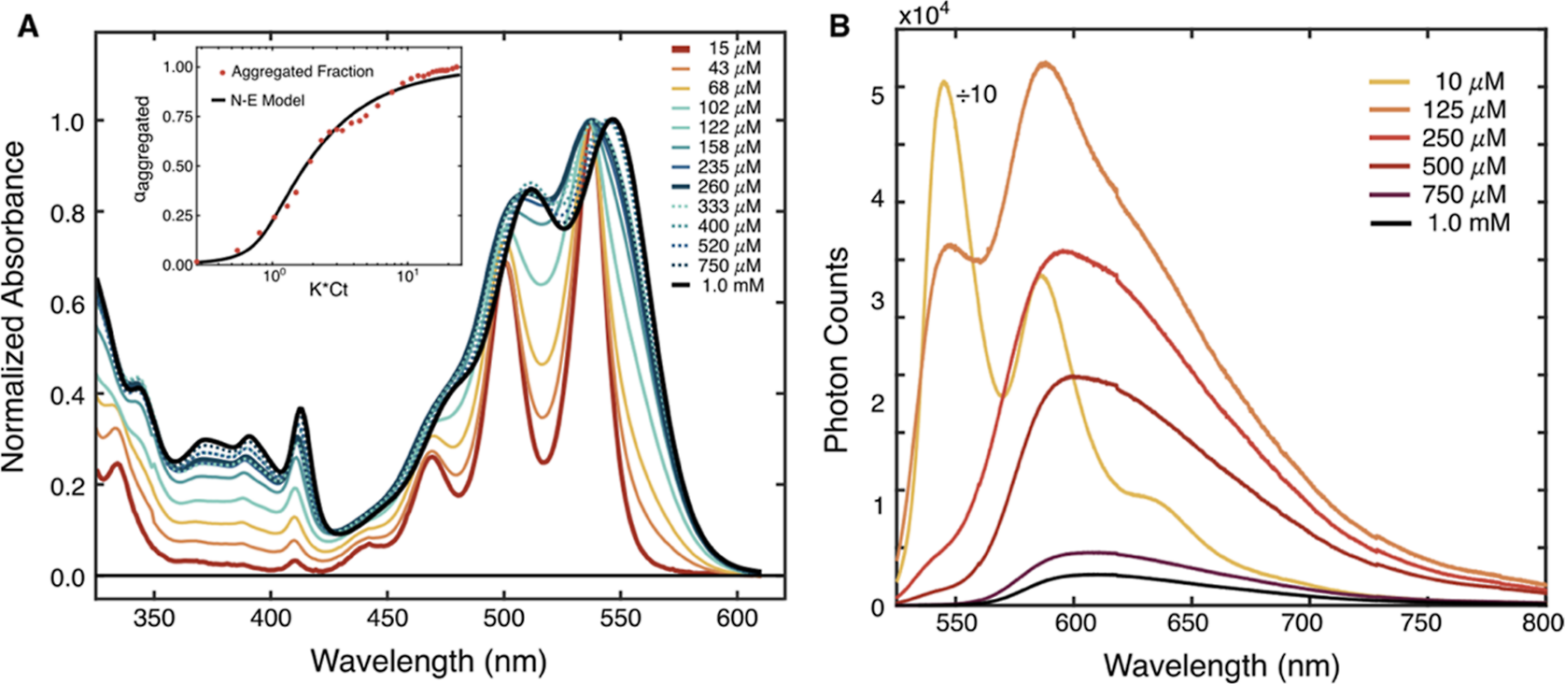
(A) Steady-state absorption and (B) photoluminescence (PL) spectra of **Tc-DA** in DMF as a function of concentration ranging from 15 μM to 1.0 mM. Bolded curves signify key concentration regimes. PL spectra were collected after excitation at 505 nm and scaled according to the proportion of absorbed photons as judged by optical density at the pump wavelength. The inset of (A) shows the fraction of **Tc-DA** aggregates vs concentration (red dots) as determined through a low- and high-concentration spectral basis approach and simulated with a nucleation–elongation model (N–E, black line), described in the Supporting Information.

An initial presumption is that the carboxylic acid functionality of **Tc-DA** naturally leads to more complex interactions with the solvent environment as compared to **Tc-DE**, depending on the solvent polarity and ability to participate in hydrogen bonding. As the concentration of **Tc-DA** is slowly increased in DMF (Figure 2A), the absorbance spectra no longer maintain a uniform shape or follow a linear concentration–absorption Beer–Lambert relationship, which we initially observed in THF (Figure S3). Increasing the concentration of **Tc-DA** up to 0.260 mM in DMF leads to broadening of the vibronic progression. Additionally, the 0–1 of the ^1^L_a_ band red-shifts from 501 to 508 nm, and the ratio of *A*_0–0/0–1_ decreases from 1.43 to 1.19. As the concentration is increased to 1.0 mM, the 0–0 of the ^1^L_a_ band further red-shifts by roughly 50 meV and settles at 550 nm without a significant change in *A*_0–0/0–1_.

Using the lowest and highest absorption spectra as bases (Figure 2A, 15 μM and 1.0 mM, respectively), an isotherm of “aggregate character” was generated and fit using a nucleation–elongation model (Figure 2A, inset).^26,27^ The resulting constants for dimerization and elongation are 345 and 23,000 L/mol, respectively (Figure S13). The fitted dimerization constant is within the range found by Würthner et al. for a series of PBIs with H-bond and π-stack motifs that are highly similar to that of **Tc-DA**.^26,28,29^ The relatively large elongation constant is indicative of a strong cooperativity. After solvation effects are overcome at a critical concentration, the driving forces for aggregation (H-bonding and π-stacking) rapidly drive the system beyond dimers. In addition to the red shift and broadening of the lowest absorption, the diminutive band at 350–420 nm grows more intense as the concentration is increased. It is noteworthy that the 1.0 mM absorbance profile of **Tc-DA** in DMF matches the concentration-independent absorbance profile found for **Tc-DA** in THF (Figure S11). This behavior appears to be unique to the carboxylic acid functionality of **Tc-DA** because the absorption of **Tc-DE**, on the other hand, remains concentration-independent in any solvent studied (chloroform, THF, and DMF; Figures S5–S7).

The PL spectra and observed emission lifetimes of **Tc-DA**, collected via time-resolved photoluminescence spectroscopy (TRPL), are similarly concentration-dependent (Figure 2B). At concentrations of up to approximately 20 μM, the emission spectrum of **Tc-DA** possesses three well-resolved features at 545, 585, and 637 nm that mirror the vibronic progression from absorption. A single exponential lifetime of 13.9 ± 0.04 ns is found for this band (Figure S19). Concurrent with broadening in the absorption spectrum, a change in the overall PL spectrum is observed at 0.125 mM, where the vibronic structure is still apparent but now convolved with a broad emissive feature, centered at 600 nm. At 0.25 mM, global fitting of the time-resolved emission is best described through inclusion of two emissive lifetimes, 4.6 ± 0.1 and 14.4 ± 0.1 ns (Figure S21). The shorter-lived component has a broad profile peaking at 600 nm, with a tail that extends past the detection window of 800 nm. The longer-lived component appears to be a broadened vibronic progression with a significantly diminished 0–0 feature centered at 550 nm and an intense 0–1 band. Further increase in concentration from 0.30 to 1.0 mM has no impact on the overall PL shape, but the intensity is significantly quenched. Global fitting of the time-resolved emission spectra at 1.0 mM remains best represented with two emissive lifetimes, 7.1 ± 0.2 and 15.2 ± 0.1 ns (Figure S23). The concentration-dependent emission implies conversion from a monomeric state at low concentrations into a new species at higher concentrations characterized by broad and weak emission.

### Nuclear Magnetic Resonance

In addition to the steady-state optical clues about aggregation, the chemical environment of the aromatic protons is distinct between different structures and could allow for further differentiation. ^1^H NMR diffusion-ordered spectroscopy (DOSY) was used to support the existence of the aggregates within this system. At 3 mM in d^7^-DMF, **Tc-DA** exhibits a single population diffusing at (4.6 ± 0.2) × 10^–10^ m^2^ s^–1^ (Figure S88). Through the Stokes–Einstein relationship *D* = *k*_b_*T*/6πη*r*_h_, where *D* is the experimentally measured diffusion coefficient, *k*_b_ is the Boltzmann constant, *T* is temperature, and η is the solvent viscosity, a hydrodynamic radius (*r*_h_) of 5.8 ± 0.2 Å was calculated.^30^ A similar DOSY experiment was performed on **Tc-DE** in CDCl_3_ (due to higher solubility) and again, a single species was found to be diffusing with a calculated hydrodynamic radius of 5.2 ± 0.2 Å (Figure S89). This hydrodynamic radius is larger than that found for **TIPS-Tc** in a previous study (*r*_h_ ≈ 3.2 Å), but by less than 2-fold, which would argue against a large aggregate size or an elongated interchromophore disposition (e.g., “linked” **Tc-DA** denoted **Tc-DA-*L***) at high concentration.^31^ It does, however, argue for the presence of a stacked dimer structure, or even a stack of up to a few molecules, where the effective hydrodynamic radius is roughly 50% larger than that found for **TIPS-Tc**.

Nuclear Overhauser effect spectroscopy (NOESY) can also inform on aggregate structures because NOE signals correspond to through-space dipole–dipole coupling (both intra- and intermolecular) between protons, and the distance limitation of this interaction is ≈5 Å.^32^ NOESY was first performed on **TIPS-Tc** in CDCl_3_ to establish a baseline for NOE interactions occurring intramolecularly within a well-solvated, 5,12-substitued tetracene derivative. **TIPS-Tc** exhibits three NOE cross peaks within the aromatic region which are assigned to through-space coupling occurring between neighboring aromatic protons along the planar core (Figures S84–S85). The NOESY spectrum also contains evidence for weak through-space coupling between aromatic protons on the core to the bulky TIPS group. NOESY was performed on **Tc-DA** (20 mM in d^7^-DMF, Figure S87) and **Tc-DE** (20 mM in CDCl_3_, Figure S86). Interestingly, the NOESY spectrum of each sample displays the same aromatic NOE cross peaks found for **TIPS-Tc**. A lack of additional NOE cross peaks does not preclude the possibility of aggregates in solution, which is convincingly evident from other experiments. Instead, this result, in combination with a small hydrodynamic radius inferred from DOSY, suggests an aggregate whose geometric cross section is not significantly extended past that of a monomer and maintains a high symmetry profile that preserves chemical equivalence of protons within the system.^33^

### Calculated Ground-State Geometries and Excited-State Energies

**Figure 3:**
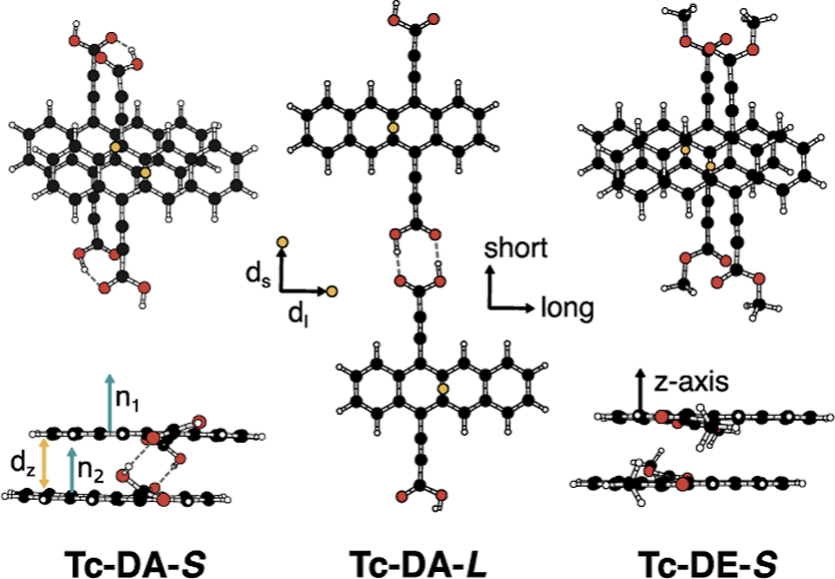
Optimized ground-state geometries for the **Tc-DA** and **Tc-DE stacked (S)** and **linked (L)** dimers. Yellow arrows indicate the direction of relative displacements of the monomer units with respect to the short (s), long (l), and *z* axes. All displacements were measured with respect to the center of mass of carbon comprising the Tc units (yellow dots). Blue arrows demonstrate normal vectors to the Tc plane (n_1_ and n_2_) and dotted lines show predicted hydrogen bonds.

Optimized geometries of ground-state monomeric and dimeric **Tc-DA** and **Tc-DE** were obtained from density functional theory (DFT) in the gas phase at room temperature (ωB97xD/def2-TZVP, see Methods/Supporting Information).^34^ We chose to study two dimer geometries for **Tc-DA**, shown in Figure 3, that can engage in π stacking or hydrogen bonding. We refer to the stacked dimer as **Tc-DA-*S*** and the hydrogen-bond-linked dimer as **Tc-DA-*L***. The monomers of **Tc-DA-*S*** that form the dimer are nearly parallel to one another, with only a 0.5° angle between vectors normal to the Tc planes (Figure 3A, blue arrows and Table 1), while the vectors normal to the **Tc-DA-*L*** dimer are parallel. The distance between the centers of mass for the carbons comprising the Tc units in the stacked dimer is 3.7–3.9 Å in total, similar to reported geometries for Tc dimers (3.3–4.0 Å).^35,36^ The interaction energy of the **Tc-DA-*S*** dimer in DMF is −33 kcal/mol, which is about 10 kcal/mol lower than that reported for Tc dimers, predicting additional stabilization from the two HO–H hydrogen bonds (Figure 3A, dotted lines).^35^**Tc-DA-*L*** has an interaction energy of −14 kcal/mol in DMF. All **Tc-DA** geometries are more greatly stabilized by solvents when compared to the corresponding **Tc-DE** geometries, and all dimers are more stable in DMF than THF (Table S3). The stabilization of the ground state of the **Tc-DA** monomer in THF (−0.36 eV) is equivalent to the stabilization energy of **Tc-DE** in DMF.

**Table 1:** Geometries for Optimized Ground-State **Tc-DA** and **Tc-DE** and Dimers^a^

	|**d**_**l**_| (Å)	|**d**_**s**_| (Å)	|**d**_***z***_| (Å)	|*r*| (Å)	θ (deg)
Tc-DA-*S*	1.5	1.3	3.4	3.9	0.5
Tc-DA-*L*	2.7	14.7	0.0	15.0	0.0
Tc-DE-*S*	1.3	0.9	3.4	3.7	0.3

a **d**_**l**_, **d**_**s**_, and **d**_***z***_ (Å) are the absolute distances in the noted direction [long (l), short (s), and *z*-axis] between the center of mass of carbons in the Tc units, as depicted in Figure 3 (yellow arrows and dots, respectively), and |*r*| is the root-mean-square distance between. Theta is the angle between vectors normal to each Tc unit (Figure 3, blue arrows).

**Figure 4:**
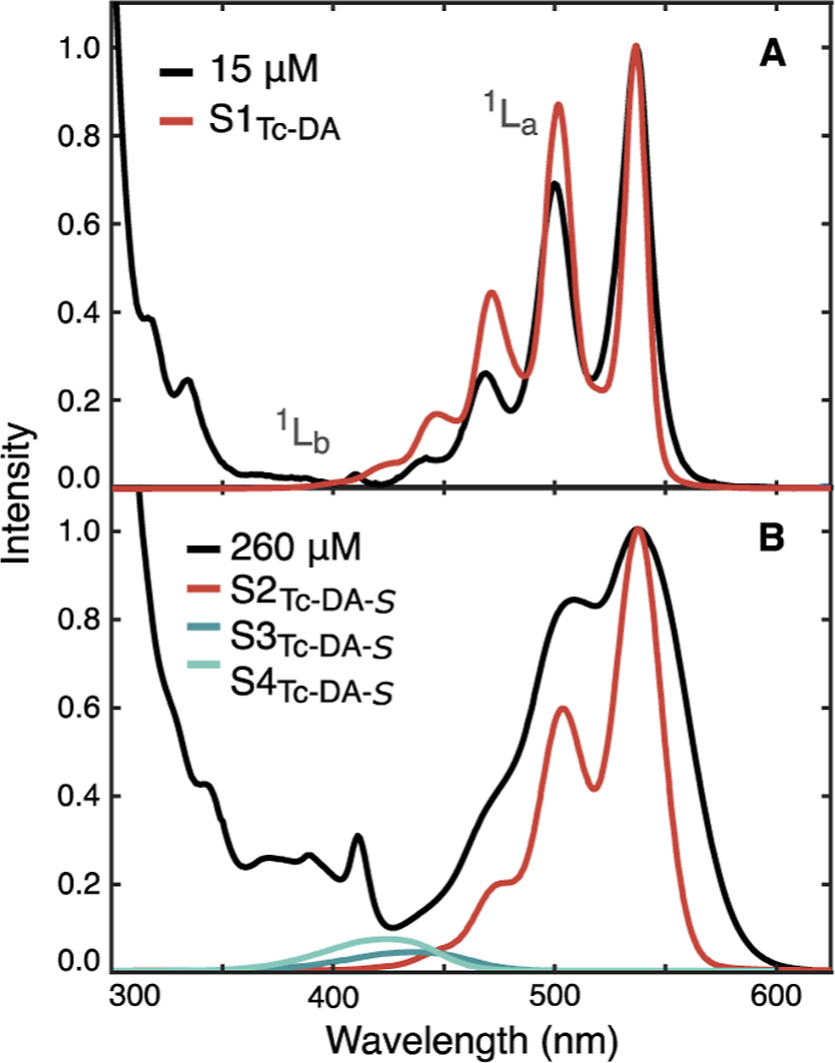
Steady-state absorption spectrum of 15 and 260 μM solutions of **Tc-DA** in DMF (black, from Figure 2A) and calculated vibrationally resolved electronic spectra for the (A) monomer and (B) stacked dimer (also see Figure S67). Signals are from the ground-state S0 to the Sn-th excited electronic state from the TD-DFT calculation; the subscript denotes the monomer (**Tc-DA**) or dimer (**Tc-DA-*S***) geometry. All lines corresponding to the dominant progression are normalized to one and the same normalization factor is applied to higher excited-state transitions. Transitions are shown at their vertical (bottom of the well) energies, which are all shifted by 0.1 eV (≈21 nm) for better comparison to data. Only transitions with appreciable oscillator strength in the ^1^L_a_ and ^1^L_b_ regions are shown (>0.005).

The excited-state electronic structure corresponding to the optimized ground-state geometry was calculated with time-dependent DFT (TD-DFT). Using the ground-state frequencies from DFT, the vibrationally resolved electronic spectra were calculated using the vertical gradient (VG) model for vibrational analysis and the time-dependent formulation for intensities.^37^ The calculated monomer spectrum agrees with the data from **Tc-DA** at low concentrations (15 μM, Figure 4A). The calculated **Tc-DA-*S*** spectrum not only broadens with respect to the monomer spectrum but also features broad overlapping bands in the ^1^L_b_ region, like those that grow at higher concentrations (260 μM, Figure 4B). The spectrum from **Tc-DA-*L*** (Figure S90C) is narrow and like the monomer features only a dominant signal from the ground state to the S1_Tc-DA-*L*_ progression. The dominant configuration that contributes to the S1_Tc-DA_ monomer state (98%, Table S4) and the S2_Tc-DA-*S*_ dimer state (77%) is from the HOMO → LUMO electronic configuration. The S3_Tc-DA-*S*_ and S4_Tc-DA-*S*_ excited states are dominated by HOMO → LUMO + 1 (70%) and HOMO – 1 → LUMO + 1 (73%) contributions, respectively. **Tc-DE** spectra follow those of **Tc-DA** (Figure S90A).

### Excitonic Coupling

For tetracene-based chromophores, the S_1_← S_0_ (^1^L_a_) transition dipole moment is oriented along the short axis of the chromophore. By comparing the three proposed structures and the predicted single-chromophore transition dipole moments for the S_0_ → S2_Tc-DA-*S*_ stacked dimer transitions and S_0_ → S1_Tc-DA-*L*_ linked dimer transitions (Figure S91), we assign the π-stacked dimers as H-like dimers and the linked dimer as a J-like dimer from Kasha’s theory. The observed **Tc-DA-*S*** spectrum in Figure 2A red-shifts at concentrations greater than 260 μM, but it shows a decreasing *A*_0–0/0–1_; it shows signatures of both Kasha dimers. The spectrum from H-like dimers blue-shifts, so the 0–0 of the blue-shifted ^1^L_a_ band that overlaps with the 0–1 of the ^1^L_a_ monomer band may contribute to the appearance of a decreasing *A*_0–0/0–1_ (Figure 2A). This explanation further supports the asymmetrical broadening of the 0–1 peak within the ^1^L_a_ band in the observed spectrum. Only small perturbations from the calculated energies would be necessary to achieve the range of shifts observed.

The complex interplay between Coulomb and CT coupling in the expanded Kasha model by Hestand and Spano means that the oft-used observables of absorption spectral envelope and shift from monomer to aggregate may not immediately reveal the structure.^38^ Attempts to fit steady-state absorption spectra using global analysis are shown in Figure S13 and generally support the HJ mode of coupling for **Tc-DA**. The H-like Coulomb coupling and J-like CT coupling interfere, yielding almost no shift but considerable broadening, as the intermolecular distance is reduced below 4 Å. Though the small particle radius measured via DOSY does not suggest the formation of large aggregates, growth of aggregates beyond the dimer through further stacking is possible at high concentration. This moderate growth beyond the dimer could explain the further red shift in the ^1^L_a_ band going from 0.25 to 1.0 mM. However, it is impossible to predict all dimer geometries using the computational framework herein, especially when considering large aggregates and the role of solvent interactions. An explicit solvation study, which captures the dynamic and specific interactions with solvents and may also improve our understanding of the nucleation–elongation equilibrium, is left to future work.

### Transient Absorption Spectroscopy

**Figure 5:**
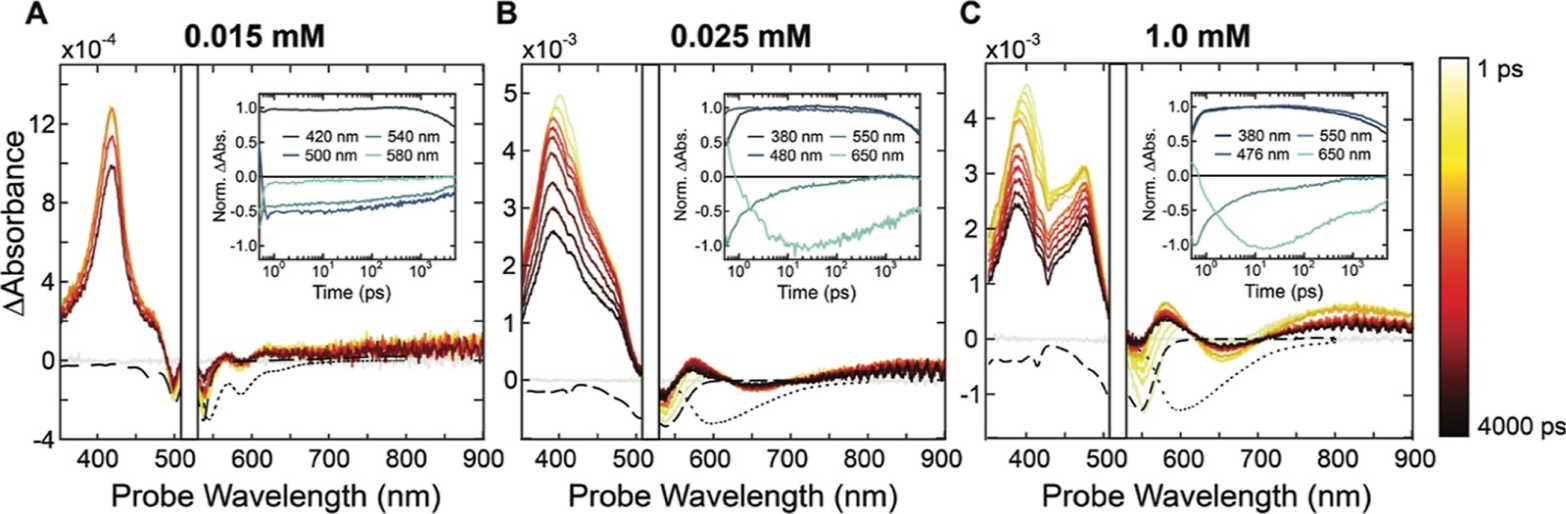
Transient absorption spectral slices from 1 to 4000 ps of **Tc-DA** in DMF at concentrations of 0.015 mM (A), 0.25 mM (B), and 1.0 mM (C) after excitation at 520 nm with 55 μW. The negatives of the ground-state absorption and emission spectra for each concentration are included as dashed black lines in each panel for clarity of spectral assignments. Single wavelength kinetic traces are provided as an inset in each panel.

To uncover consequences of the aggregation on the excited-state dynamics for **Tc-DA** in DMF, we turned to transient absorption spectroscopy, which was performed on samples at three concentrations representing distinct behaviors gleaned from steady-state experiments: 0.015, 0.25, and 1.0 mM. An excitation wavelength of 520 nm was chosen as a compromise between sufficient absorption for each sample at this wavelength and minimization of pump scatter in regions of particular interest. Transient absorption data for **Tc-DA** at 0.015 mM in DMF reflect the monomeric behavior (Figure 5A) inferred from the absorbance and fluorescence spectroscopy shown above. After selective excitation at 520 nm, approximately 60 meV higher than the 0–0* transition, a ground-state bleach (GSB) appears, which matches the steady-state absorption profile. Concomitantly, a strong excited-state absorption (ESA) feature centered at 420 nm appears within the instrument response time along with broad ESAs extending from 600 to 900 nm and in the near-infrared (NIR) region centered at 1287 nm (Figure S27). The decay of these ESAs matches the recovery of the GSB with a lifetime that extends well past the 5 ns delay stage. At longer pump/probe delay times accessible with a separate electronically delayed probe, we find the same spectral profile decaying with a lifetime of 14.2 ± 0.1 ns (Figure S29). The decay of this feature is coincident with the rise of a small signal centered at 500 nm that decays with an exponential component of 130 ± 8 μs, determined from global analysis. We assign the 14.2 ns species to the **Tc-DA** first singlet excited state (S_1_) and the 130 μs component to the triplet state (T_1_), which is formed through a low yield of intersystem crossing. The spectral profiles and lifetimes of these excited states are in good agreement with those found for the first singlet and triplet excited states of the well-solvated **TIPS-Tc** measured in THF (Figures S26, S52, and S53).

Exploring the dynamics at 0.25 mM (Figure 5B), a broadened GSB appears after initial photoexcitation, which reflects the change in the ground-state absorbance spectrum for this higher concentration from Figure 2A. Concomitant with the GSB formation, a broad ESA centered at approximately 400 nm rises and shifts to 388 nm across a time scale of a few picoseconds, where it then remains and decays by approximately 65% through the 5 ns experimental window. An additional broad ESA from 570 to 900 nm is immediately present and appears to be overlaid on a broad stimulated emission profile. In the NIR spectrum, an intense ESA at 1300 nm forms immediately after excitation, similar to the 0.015 mM sample (Figure S31). This NIR ESA shifts to 1260 nm within 10 ps, where it persists for the remainder of the 5 ns time window. After the early picosecond dynamics, the spectral profile remains constant as ESAs and GSB diminish concurrently. An accurate global fit was found with two exponential decay components of 5.4 ± 0.6 and 13.6 ± 1.2 ns, in agreement with the lifetimes found through TRPL (Figure S21).

At the highest concentration of 1.0 mM (Figure 5C), the transient absorption profile was found to change remarkably. After excitation at 520 nm a GSB forms, centered at 550 nm, reflecting the red-shifted steady-state absorption profile shown in Figure 2A. Interestingly, the GSB appears to significantly diminish in intensity within approximately 100 ps, indicating overlap with a strong ESA. Concomitant with the formation of the GSB, a double-humped ESA in the near-UV is observed with two prominent features centered at 399 and 475 nm. The spectral profile in this region is in stark contrast to that of the lower-concentration samples, which display only a single ESA to the blue of the excitation wavelength. The higher-energy feature at 399 nm shifts by 90 meV across a 100 ps time scale and settles at 388 nm where it then decays in intensity by about 30% through the remainder of the 5 ns time window. The second feature at 475 nm does not shift as a function of delay time nor does it decay as rapidly. Instead, it gains approximately 20% more amplitude across the initial 10 ps and then decays by 40% across the 5 ns time window.

At longer probe wavelengths, a broad ESA feature and broad stimulated emission profile from 580 to 900 nm are superimposed. Stimulated emission appears to grow roughly 3-fold more intense across 10 ps and then decays to baseline across the 5 ns experimental window. In the NIR, a single ESA appears at 1250 nm immediately after excitation and then shifts to 1185 nm where it persists for the remainder of the delay (Figure S35). Beyond a 100 ps delay, there are no further changes in the transient absorption spectrum. Extending from the nanosecond to microsecond time regime, all ESAs decay and the GSB recovers within 50 ns, and the data are again well-fit by two exponentials, 6.4 ± 0.5 and 17.5 ± 1.2 ns.

**Figure 6:**
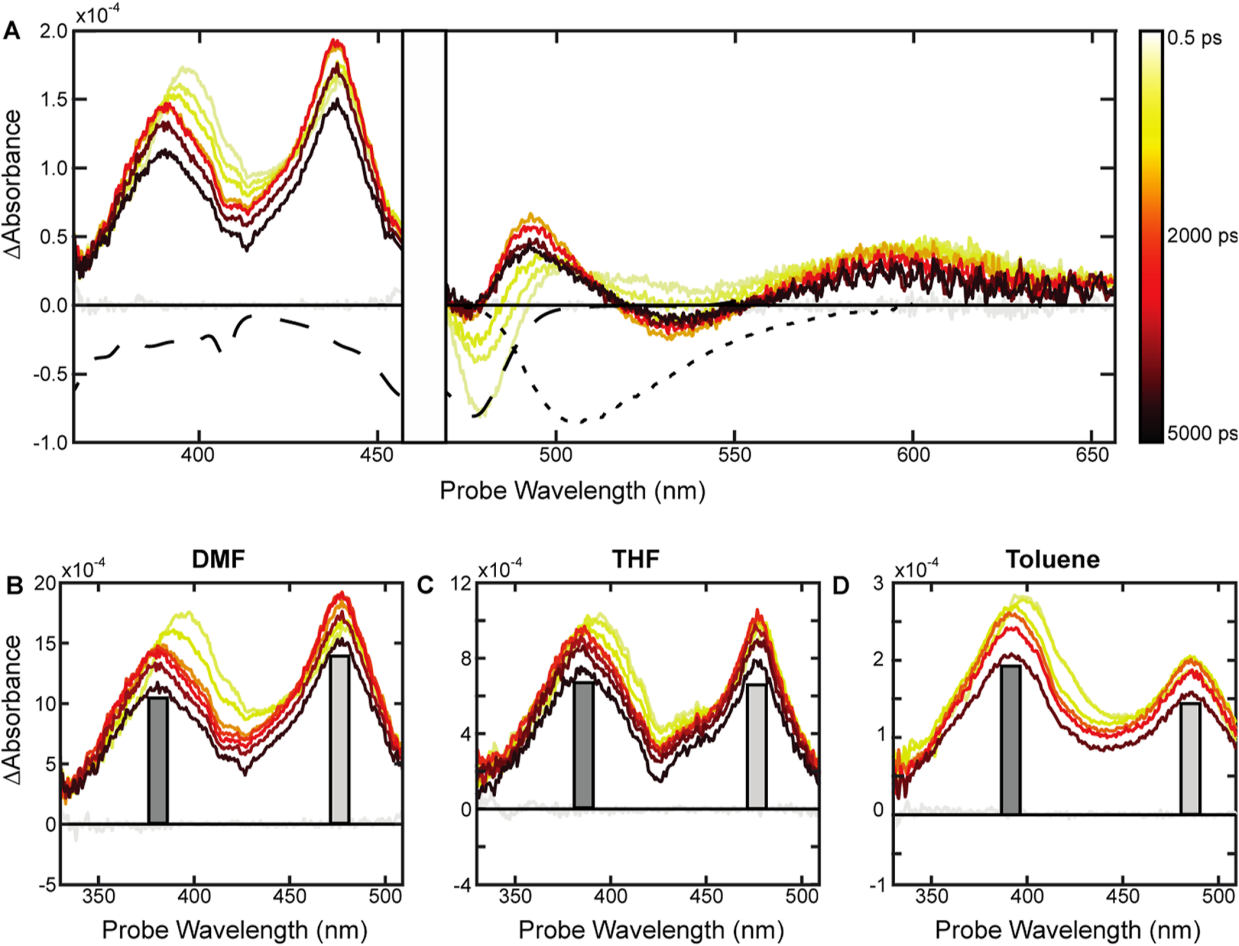
(A) Spectral slices from the transient absorption experiment of 0.1 mM **Tc-DE** in DMF after excitation at 520 nm with 55 μW. (B–D) Spectral slices in the range of 330–510 nm for 0.1 mM **Tc-DE** in DMF, THF, and toluene. Bars are included to guide the eye for relative amplitudes of primary features.

A similar set of transient absorption experiments were performed on **Tc-DE** in DMF and are highlighted in Figure 6A. Interestingly, the transient absorption behavior of **Tc-DE** at 0.1 mM (Figure 6A) is nearly identical to that found for **Tc-DA** at 1.0 mM (Figure 5C) and does not show a strong concentration dependence on its photophysics (Figures S40 and S46). The ESAs found at 380 and 476 nm have not been observed in any previously reported tetracene systems and appear to be unique to **Tc-DE** and **Tc-DA** (at higher concentrations). The exact nature of these transitions and why they emerge as a function of the concentration for **Tc-DA** will be discussed below.

The enhanced solubility of **Tc-DE** was leveraged to access solvents with varying polarities and further probe the electronic state with an approximately 16 ns lifetime. Aprotic solvents toluene, THF, acetone, and DMF were used to span a range of polarities (Figures 6 and S40, S47, and S50). When placed in nonpolar toluene, the photophysics and overall transient profile resemble those of **Tc-DE** in DMF, but two differences emerge (Figure S47). Most importantly, the stimulated emission of **Tc-DE** in toluene does not become more intense as time progresses, unlike in DMF. Second, after 100 ps, there is no further spectral evolution, leaving the two prominent ESAs in the near-UV maintaining a relative ratio of *A*_380 nm/476 nm_ = 1.33, which is higher than the 0.75 ratio found in DMF at a similar concentration. This ratio appears to track with solvent polarity, where *A*_380nm/476nm_ is 1.33, 0.95, 1.05, and 0.75 for toluene, THF, acetone, and DMF, respectively (Figures 6B–D and S50).

### Assignment of Excited-State Character

The initially formed excited state of **Tc-DA** at 0.015 mM exhibits a strong ESA centered at 420 nm and narrow GSB, with lifetimes that are traditionally found for well-solvated tetracene derivatives in solution. Thus, this is assigned to the S_1_ electronic state in the purely monomer regime. At 0.25 mM, the S_1_ of **Tc-DA** exhibits broadened ESA features and stimulated emission (Figure 5B, 1 ps spectrum) compared with 0.015 mM **Tc-DA** in DMF (Figure 5A), consistent with the distinction found for calculated dimerization of **Tc-DA** subunits (S0_Tc-DA-*S*_ → S2_Tc-DA-*S*_, Figure 4B). We assign this broadened dimer signal to the SF precursor commonly referred to as S_0_S_1_. For **Tc-DE** and 1.0 mM **Tc-DA**, the previously assigned S_0_S_1_ excited state is no longer visible and instead is characterized by a bifurcated ESA in the near-UV. The new spectral features in transient absorption and their distinct evolution with time and solvent polarity suggest involvement of a state with intermolecular character. This could be excimer, intermolecular charge resonance (CR) or CT, or a correlated triplet pair state (TT) that would be expected for the initial step of SF. The lack of spectral resemblance to **Tc-DA** at lower concentrations or to other tetracenic systems in the literature suggests mixed character that requires explicit characterization of the relevant electronic basis states, which would contribute to this intermolecular electronic state.

**Figure 7:**
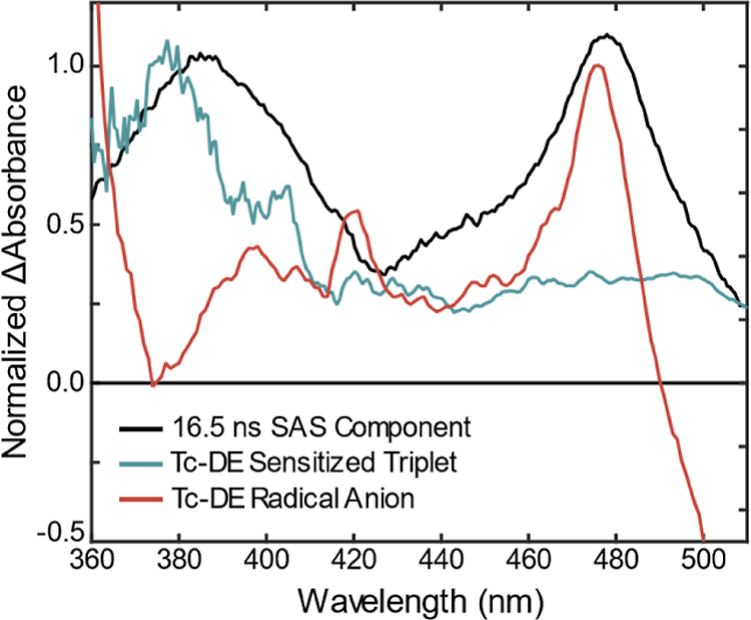
Comparison of the differential absorption spectra for the states Tc-DE*, ^**3**^**Tc-DE**, and **Tc-DE**^•**–**^ all in THF.

Triplet sensitization was performed on **Tc-DA** and **Tc-DE** through use of palladium(II) *meso*-tetraphenyltetrabenzoporphyrin (T_1_ = 1.56 eV) to support the assignment of TT character resulting directly from the **Tc-DA** and **Tc-DE** S_0_S_1_ states.^39^ This claim is substantiated by the subsequent emergence of a UV ESA band at ≈380 nm in the sensitized triplet absorption spectrum of **Tc-DE** (Figure 7, teal line; see also Figure S54) that closely matches the peak in the 16.5 ns component obtained through transient absorption global analysis (Figure S45). Interestingly, this near-UV band is also present for ^3^**Tc-DA** when its ground-state absorption spectrum resembles that of **Tc-DE** such as in THF or at high concentrations in DMF (Figures S57 and S59). In contrast, the triplet ESA at low concentrations in DMF (20 μM, Figure S60) matches that of ^3^**TIPS-Tc** (Figure S53), as well as most other tetracene derivatives studied in the literature, with a band only from 400 to 500 nm and the most prominent triplet ESA feature centered at approximately 500 nm. Without sensitization, the unique near-UV triplet ESA feature is found for both high-concentration **Tc-DA** (Figure 5C) and **Tc-DE** (Figure 6), the latter of which is invariant in concentration and solvent. The lack of previous reports for triplet absorption in the near-UV means precedence in other tetracene derivatives or aggregates is difficult to verify. Nonetheless, we assign the features to the triplet formed in an aggregated species, spectrally distinct from that of isolated tetracene.

Thermodynamic feasibility of TT formation is estimated by the energetic difference between the excited singlet state (S_1_) and the TT state which can be approximated as 2 times the triplet energy (Δ*E*_SF_ = S_1_ – 2T_1_). The triplet energy of **Tc-DA** was computationally predicted to be 0.91 eV (Table S1) in DMF, which is quite low compared to the reported triplet of **TIPS-Tc** (1.25 eV).^31^ Experimentally, a T_1_ of 1.14 ± 0.06 eV was found for **Tc-DE** by using five different triplet sensitizers and fitting measured *k*_TET_ values against the donor’s triplet energy in a Sandros plot (Figures S62–S67).^40^ Assuming that the triplet energy is not significantly perturbed between the structural analogues, **Tc-DE** and **Tc-DA**, formation of the TT would be roughly isoergic (S_1_ = 2.18 eV for **Tc-DE**) and more favorable in comparison to **TIPS-Tc** which is approximately endoergic by 190 meV. The formation time of the TT component extracted from the global fit (ranging from 3 to 7 ps; Figures S37 and S41) also supports the notion of a nearly isoergic process. Further supporting the TT assignment is the relatively short lifetime of this state (≈15 ns) compared to the long-lived sensitized single triplet of 1.0 mM **Tc-DA** (≈180 μs; Figures S55, S57, and S59). For the intermolecular state, the near resonance between TT and S_0_S_1_ (≈70 meV in THF; Figure S11) dictates that decay kinetics are similar to that of pure S_1_ for monomers. The lack of available outlets for TT dissociation limits opportunities for long-lived triplet formation, which is a topic addressed in studies of tetracene supramolecular structures and could be ameliorated in larger aggregates.^41^

The ESA profiles of ^**3**^**Tc-DA** and ^**3**^**Tc-DE** are spectrally distinct from the S_0_S_1_ state, but they match only one of the bands of the bifurcated ESA in the near-UV. This prevents our assignment of the correlated triplet pair to being solely responsible for the electronic state formed through aggregation of **Tc-DA** and **Tc-DE** subunits. We further evaluated the possible involvement of a CT state by characterizing the spectral profiles of the radical anion and cation of **Tc-DE** (**Tc-DE**^•**–**^ and **Tc-DE**^•**+**^, respectively) via spectroelectrochemistry (chemical oxidation was required for the radical cation, see Figures S74–S75). We find that the most prominent absorption band of **Tc-DE**^•**+**^ occurs in the near-UV at 360 nm (Figure S75). On the other hand, for **Tc-DE**^•**–**^, the most prominent feature occurs at 476 nm (Figure 7, red; see also Figure S73) which closely matches the unassigned lower-energy absorption band of the bifurcated ESA feature. The presence of the radical anion feature in the transient absorption spectrum, along with the strong CR character found in the ground state of dimer species through computational modeling (Figure 4), would suggest that the excited state has a prominent CT character. The CT energy (2.31 eV; see Supporting Information for further details) also lies close in energy to the lowest excited singlet. Furthermore, the manifestation of the spectrally distinct features associated with ^**3**^**Tc-DA** and **Tc-DE**^•–^ along with their simultaneous decay implicates a CT and TT mixed state in **Tc-DE** and **Tc-DA** aggregates. The presence of stimulated emission from this state implies at least weak radiative recombination from either the CT or the TT component. In addition, we find that the CT character becomes more pronounced, evidenced from the increased amplitude at 476 nm (Figure 6), as the solvent polarity is increased. The steady-state and time-resolved emission experiments also track with increased involvement of CT in the excited state as the radiative rate constant decreases with solvent polarity going from 5.4 × 10^7^ to 4.6 × 10^7^ to 2.7 × 10^7^ s^–1^ as the solvent is changed from toluene to THF to DMF.^42^ On the other hand, the TT component of the mixed state is expected to be nonemissive (except in rare circumstances).^43,44^ The formation time of the CT component near 480 nm decreases with the solvent polarity (Figures S41 and S48), suggesting its further stabilization and underpinning the increase in its amplitude with respect to the TT component (Figure 6). The clear involvement of CT states in TT formation predicted for linked tetracene dimers is juxtaposed here with the possibility that excessive CT character could create a “sink” that hinders evolution toward TT.^45,46^

The other excited-state species, found only in **Tc-DA** at intermediate and high concentration, possesses broad features throughout the ESA, bleach, and stimulated emission and lacks the sharp vibronic bands that are present at lower concentrations (Figure 5B). This species forms in less than a picosecond after photoexcitation and decays primarily with a 5–7 ns time constant in both TA and TRPL (Figures S21, S23, S33, S37, and S39). These properties imply a distinct species with some TT character, as the triplet ESA at 390 nm remains as a shoulder, but little to no CT character, as the sharp 480 nm ESA characteristic of the anion is absent. Due to the limited aggregate size, we hypothesize that a state with incomplete charge separation forms in this regime. For purposes of distinguishing the short-range charge separation in this state from the CT state defined earlier, we call it an “excimer” due its proposed dimer-like nature. Past work on a covalent tetracene dimer with an enforced face-to-face geometry found a similar broad excited-state absorption in UV TA that was not assigned but may similarly possess this excimer/TT character.^47^ We note that such a strongly bound excimer state is much less likely for **Tc-DA-*L***.^48,49^ The broad and unstructured fluorescence spectral shapes associated with the approximately 15 and 5 ns components in the intermediate- and high-concentration regimes match what is observed for stimulated emission in TA (Figures S21 and S23). There is a general trend toward weaker steady-state and stimulated emission as the concentration increases (e.g., Figure 3), which is expected as both pure CT and TT character increases at the expense of the emissive excimer. However, the near-identical spectra of the two emission components suggest that the excimer may remain involved as a minor species, kinetically connected to the dominant but likely dark CT/TT.

**Figure 8:**
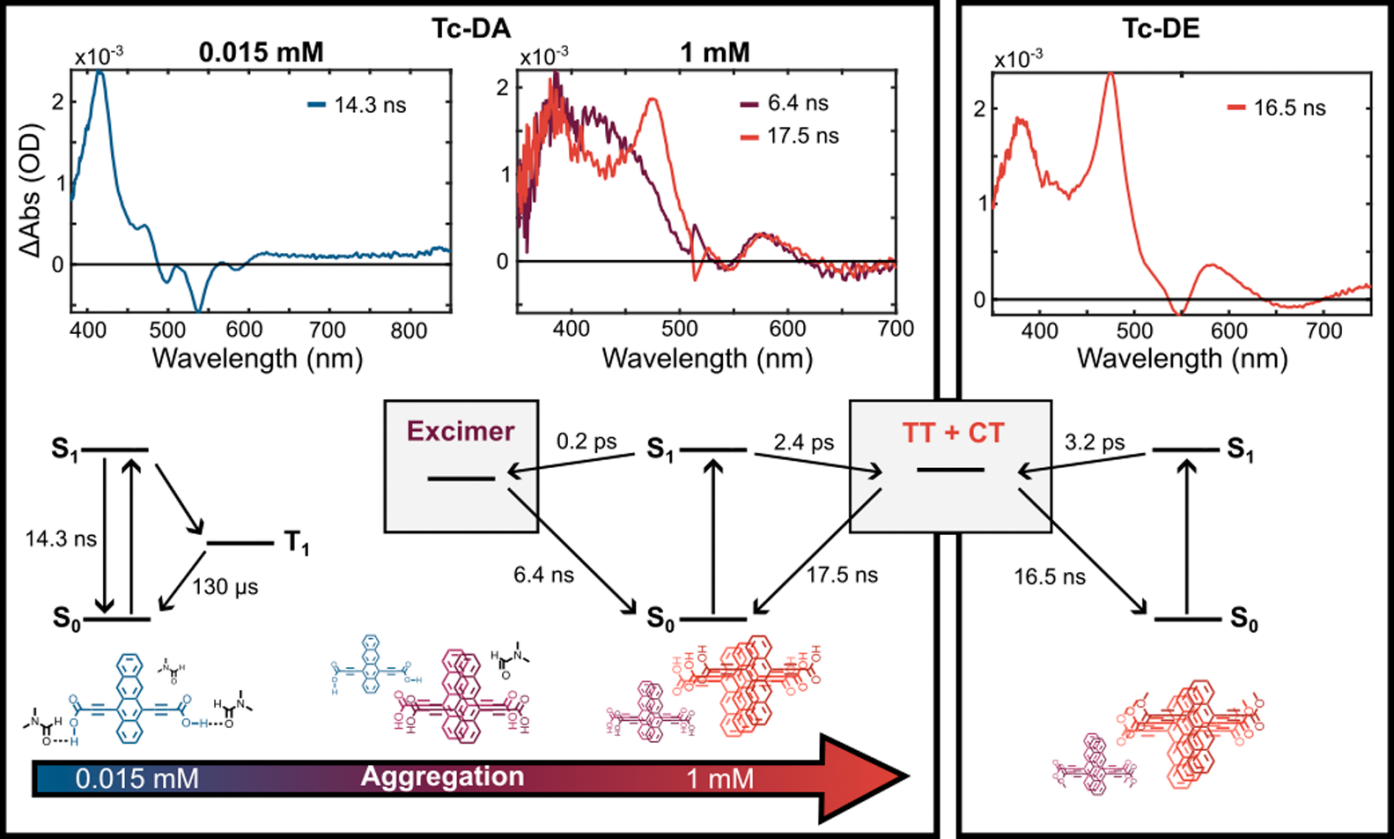
Species-associated spectra and their respective excited-state decay lifetimes for monomeric 0.015 mM **Tc-DA**, aggregated 1.0 mM **Tc-DA**, and aggregated 0.1 mM **Tc-DE**. The corresponding Jablonski diagram depicting the photophysics for each sample is depicted above. The lifetimes in the schemes are derived from a global fit of the transient absorption data sets employing kinetic models described in detail in the Supporting Information. Schematic structures representing the dominant species in each regime are found below each scheme.

From left to right in Figure 8, the evolution from monomer-like ESA to a single broad ESA and a bifurcated ESA supports the heuristic model: S_0_S_1_ → excimer + TT/CT. However, the kinetics of ultrafast excimer formation vs ps-scale CT/TT formation is incommensurate with the relative yields, which are evidently similar at intermediate concentrations. Therefore, we hypothesize that multiple aggregate species are present at intermediate concentration, with some that are predisposed to excimer formation due to differences in geometry with those that favor CT/TT. Furthermore, the continued red shift of the steady-state absorption and PL spectrum going from 0.25 to 1.0 mM suggests that aggregates of **Tc-DA** are larger than dimers at higher concentrations, and these aggregates may support and stabilize CT/TT vs excimer formation. In the lower portion of Figure 8, we illustrate the outcomes of these proposed models in which the initial excited state changes from an ensemble of excimer and CT/TT states for **Tc-DA** at intermediate and high concentrations, contrasting with the CT/TT exclusively for **Tc-DE** at all concentrations.^35−37^ The ensemble model that fits the higher-concentration **Tc-DA** TA data on a femtosecond time scale is demonstrated through the species-associated spectra shown in Figure S37. The initial component in the CT/TT pathway already has features reminiscent of the superposition of the triplet and radical anion, and its evolution through the first nanosecond suggests further relaxation, including a channel that may reversibly connect to the emissive excimer, yielding a small amount of residual stimulated emission throughout its roughly 17 ns lifetime that was also detected in time-resolved fluorescence (vide supra).^50^ On the other hand, the initial component of the excimer pathway resembles the localized S_1_ of the monomer, before rapidly converting to a broadened single peak that decays in approximately 6 ns. The fact that this latter pathway persists at higher concentrations can be attributed to the retention of some population of dimers even as the aggregate size distribution shifts and broadens.

**Tc-DE**’s behavior, which does not participate in hydrogen bonding, is similar to that of **Tc-DA** only at its highest concentration (1.0 mM). **Tc-DE**, however, avoids excimer formation under all conditions, and its absorption spectrum is largely insensitive to the concentration or solvent (Figure 1). Figure 6 demonstrates the only exception, which is that the degree of CT vs TT character is modulated by solvent polarity. Fluorescence from **Tc-DE** in DMF does not contain a short-lived component and is best represented by a single exponential decay on a 16 ns time scale. **Tc-DE**, unlike **Tc-DA,** thus appears to be aggregated at all concentrations, implying that intermolecular forces other than hydrogen bonding, such as π–π interactions, are dominant over solvent–solute interactions. These strong interactions may be unquenched upon dimerization, so that larger aggregates form at very low concentrations. This contrasts with hydrogen-bonded **Tc-DA,** wherein the monomer to dimer equilibrium can be characterized with a nucleation–elongation model through the available concentration range, as shown in the Figure 2A inset. Because no excimer-like component exists in the **Tc-DE** global analysis, we hypothesize that the excited-state motions necessary to stabilize the excimer, which likely include interplanar contraction and solvent dynamics, may be sluggish in larger aggregates.^51^ As Figure 3 demonstrates, the different geometries predicted for **Tc-DA** and **Tc-DE** dimers may also play a role in facilitating CT/TT formation in **Tc-DE**, which more readily facilitates stacking, unimpeded by hydrogen bonds that distort **Tc-DA** dimers.

Interestingly, the aggregates that form for both **Tc-DE** and **Tc-DA** appear to have a critical limit to their size apparent from the single, and relatively small, diffusing population found through DOSY (Figures S87–S89). We postulate that there are two factors that influence the equilibrium aggregate size in these systems: enthalpic and entropic.^52^ For the **Tc-DA-S** aggregate, enthalpic stabilization will likely diminish as subunits are added beyond the dimer as the hydrogen bonds that strongly drive aggregation become quenched. Similar arguments can be made for **Tc-DE**, as π-stacking that initially drives aggregation is balanced by entropy loss as aggregates grow. For **Tc-DE**, the initial π-stacking that drives dimer formation is enthalpically driven with a favorable interaction energy of −26.8 kcal/mol (Table S3). How this interaction changes for larger aggregates is unknown but is undoubtedly smaller than that for the dimer. Further aggregation and enthalpic gains are thus balanced by a loss of entropy within the system that leads to a critical limit for the size, as the thermodynamics switches from favorable to unfavorable for further aggregation. What is unique about **Tc-DA-S**, however, is that the specific **Tc-DA**/DMF interactions that confer solubility and isolation to **Tc-DA** molecules at low concentration likely result from hydrogen bonds between DMF and –COOH. The network of solvent–solute hydrogen bonds around **Tc-DA** is disrupted as dimerization occurs, potentially increasing the entropy in this step and creating a stable **Tc-DA-S** species in a specific concentration range. Once the solution contains primarily dimers, the trend with concentration becomes similar to that of **Tc-DE**, where reduced entropy of aggregation balances enthalpic gains as the aggregates grow. The balance in strength between **Tc-DA**/DMF and **Tc-DA**/**Tc-DA** hydrogen bonding yields a liminal dimer species with distinct photophysics in a narrow concentration regime, which evidently possesses the appropriate geometry to quickly form the excimer. Eventually, π–π interactions dominate at the highest concentrations, revealing the convergence of **Tc-DA** and **Tc-DE** behavior.

## Conclusions

From the point of view of conventional aggregation models, **Tc-DA** and **Tc-DE** present striking and unexpected contrast. For **Tc-DA**, changes in steady-state and time-resolved absorption spectra characterize the transition from monomer to aggregate-dominated behavior that is separately modulated through concentration- and solvent-specific interactions with installed functional groups. We surmise from transient absorption analysis and predicted intermolecular geometries that a narrow regime exists in which **Tc-DA** hydrogen-bonded dimer behavior dominates the photophysics, a regime absent from that of **Tc-DE**. To our knowledge, evidence for through-space interactions at concentrations as low as 50 μM such as those shown here has not been reported for single-chromophore tetracene derivatives, implicating the hydrogen bonds exclusive to **Tc-DA** aggregates. The similar behavior exhibited between **Tc-DE** and **Tc-DA** at high concentrations, despite the lack of hydrogen bonding for the former, suggests that multiple types of aggregates are at play, including those driven by π–π interactions that extend beyond a dimer. NMR and computational studies, in combination with the trends gleaned from our spectroscopic results, allow us to build a case for aggregate structures, which is rarely achieved in solution-phase polyacenes.

The demonstrated control of noncovalent tetracene-based aggregates simply through solvent polarity and concentration leads to the formation of mixed CT and multiexcitonic states on a rapid picosecond time scale. These states are known in covalent dimers of related systems but are much less studied in noncovalent acene aggregates.^53^ Tuning the aggregate distribution rationally can affect both the SF and triplet–triplet annihilation pathways, as they both depend sensitively on the geometry that supports the bound TT state.^54^ The additional chromophores that exist in larger aggregates in **Tc-DE** or **Tc-DA** may facilitate the formation of states with TT character, but they evidently are not sufficient to drive independent T + T formation, unlike in related tetracene structures with larger excited-state delocalization.^41^ However, these aggregates do appear to separate charges efficiently as in related symmetry-breaking charge separation systems, a property that has potential value in light harvesting.^55,56^

## Supporting Information Available

Materials and methods, synthesis and characterization including NMR of all compounds, additional steady-state and transient spectroscopic characterization, electrochemical data, 2D NMR of aggregates, and electronic structure calculation details (PDF)
