# Supplementary material for: Tetracene Diacid Aggregates for Directing Energy Flow toward Triplet Pairs
Source: J Am Chem Soc. 2024 Apr 12;146(16):11473–85. doi: 10.1021/jacs.4c02058 (PMC11046478; doi:10.1021/jacs.4c02058)
Supplement: Supplementary file 1 — ja4c02058_si_001.pdf [file ja4c02058_si_001.pdf]

Supporting Information for

Tetracene Diacid Aggregrates for Directing Energy Flow toward Triplet Pairs

Nicholas F. Pompetti[1,2], Kori E. Smyser[2], Benjamin Feingold[2], Raythe Owens[2], Bimala Lama[2], Sandeep Sharma[2], Niels H. Damrauer[2,3], Justin C. Johnson*[1,2,3]

[1]National Renewable Energy Laboratory, 15013 Denver West Pkwy, Golden, CO

[2]Department of Chemistry, University of Colorado Boulder

[3]Renewable and Sustainable Energy Institute, University of Colorado Boulder

*Corresponding author email:

Justin.Johnson@nrel.gov

This PDF file includes:

Materials and Methods

Supplementary Text

Figures S1 to S91

Tables S1 to S5

Eq S1 to S4

Other Supplementary Materials for this manuscript include the following:

Materials and Methods 3

Spectroscopic Characterization 8

Steady State Absorption and Emission 8

Solvent Dependent Steady State Absorption and Emission 11

Steady-state absorption global analysis 14

Time Correlated Single Photon Counting (TCSPC) 16

Time Resolved Photoluminescence 18

Transient Absorption 22

Triplet Sensitization 36

Sandros Plot 42

Electrochemical Characterization 49

Cyclic Voltammetry 51

Spectroelectrochemistry 54

Chemical Oxidation of Tc-DE with $NOBF_4$ 55

Synthesis 56

NMR Experiments 65

Concentration Dependent ^{1}H-NMR 65
NOESY 66
DOSY 68
Electronic Structure Theory 69
Optimized geometries 73
References 86

Materials and Methods

General Methods

All reactions were run under a nitrogen atmosphere in flame-dried glassware, unless otherwise specified. Tetrahydrofuran was distilled from sodium benzophenone ketyl prior to use. Unless otherwise noted, all reagents were obtained from commercial suppliers and used without further purification. ^{1}H NMR spectroscopy was performed at 300 MHz in $CDCl_3$ using residual chloroform as an internal standard (7.26 ppm). ^{13}C NMR spectroscopy was performed at 75 MHz in $CDCl_3$ using the center line of solvent (77.16 ppm) as an internal standard.

Photophysical Experiments

Steady-state Absorption and Emission: UV-Visible absorption spectra were collected with an HP 8452A diode array spectrophotometer using 1.0, 0.2, 0.1 cm pathlength quartz cuvettes as noted. Steady-state emission spectra were collected using an Olis SLM 8000C fluorimeter with the appropriate wavelength-dependent correction (calculated as the multiplicative factor necessary to match the collected emission profiles of two solid emissive NIST standards -SRM 2940-B and SRM 2943 to their true profiles) applied to the raw data. Most of the data collected herein utilized a configuration in which the slit-widths before and after each monochromator were set to 1mm (which corresponds to an effective bandpass of 2 nm). Quantum yields were calculated relative to the emission standard 4-(dicyanomethylene)-2-methyl-6-(p-dimethylaminostyryl)-4H-pyran (DCM) which was separately measured to have an absolute quantum yield of 43% in aerated ethanol. Samples for quantum yield measurements were diluted so as to have OD at or slightly below 0.1 at the 0-0 vibronic transition of the $^{1}L_a$ band so as to minimize inner filter effects. A wavelength was chosen where both the tetracene derivative and quantum yield standard had the same optical density and fell within the range of acceptable excitation wavelengths for DCM.[1] Emission spectra were then collected within a spectral window so as to fully capture the fluorescence spectra of both samples using a 0.5s integration time at each wavelength.

Time-Correlated Single Photon Counting (TCSPC): TCSPC measurements were made using a DeltaFlex Modular Fluorescence Lifetime System from Horiba Scientific. A Horiba NanoLED-405L was used as the excitation source (405 nm center-wavelength [CWL], c. 220 ps full-width at half maximum [FWHM]). Temperatures were measured with a built-in digital temperature sensor in the Horiba device. Temperature was controlled by a Quantum Northwest TC-1 temperature control unit coupled with an aquarium pump submerged in a reservoir, and a jacketed sample holder.

Femto- and Nanosecond Transient Absorption (Helios and EOS): Coherent Libra Ti:Sapphire laser outputs pulses centered at 800 nm with repetition rate of 1 kHz, ∼ 4 W power and ∼ 100 fs pulse width. The 800 nm beam splits into two pathways: One to TOPAS, a commercial optical parametric amplifier, at right phase-matching angle, a pulse centered at 600 nm is generated as our pump beam to the sample. The other one is directed to a Sapphire crystal with different thickness to generate 450-850 nm and 750-1600 nm probe light. The delay time between the pump beam and the probe beam is controlled by the multipath delay stage for fsTA, which can reach up to ∼ 5 ns delay time. As for nsTA, the longer delay time is manipulated electronically with EOS (electro-optical system). For both nsTA and fsTA, the broadband probe beam is separated into two beams for reference detection to minimize the influence of pulse-to-pulse fluctuation to the transient absorption signal and increase the signal quality. Each beam is focused to the optical fibers and detected by a photodiode array. All samples for transient absorption experiments were prepared under an atmosphere of nitrogen unless specified otherwise.

Nanosecond Transient Absorption for Sandros Plot: A pulsed Nd:YAG laser (Continuum Surelite II) with a 10 Hz repetition rate, ~5 ns pulse width, and centered at 355 nm was used to pump a Continuum Surelite optical parametric oscillator to obtain other excitation colors. The power was attenuated to between 0.1-0.6 mW at the sample as needed using neutral density filters. The spot size was measured with a knife-edge measurement and fit to a Gaussian profile (~4mm x 4mm). The emission of a 100 W xenon arc lamp served as a broadband probe. This was focused onto the sample with a plano-convex lens (75mm focal length). The pump and the

probe were incident upon the cuvette perpendicular to each other. Individual kinetic traces were obtained by selecting the wavelength with a monochromator (resolution of ± 1.7 nm) and measuring the signal from a PMT (Hamamatsu, R928-07) negatively biased at -1000 V with a digital oscilloscope (Picoscope 5444D). Traces were fitted to a single-exponential model and in some cases could have a time-independent offset to account for longer-lived features. In instances where the single-exponential model failed, further modeling was performed as described in the text. All samples were prepared under an atmosphere of argon unless otherwise specified.

NMR Experiments

Standard 1H and 13C NMR Experiments: ^{1}H NMR spectroscopy was performed at 300 MHz in $CDCl_3$ or d^6-DMSO using the residual solvent peak as an internal standard (7.26 and 2.50 ppm respectively). ^{13}C NMR spectroscopy was performed at 75 MHz in $CDCl_3$ or d^6-DMSO using the center line of solvent (77.16 and 39.52 ppm respectively) as an internal standard.

Diffusion Ordered Spectroscopy (DOSY) Experiments: All spectra were acquired on an Agilent INOVA 400 spectrometer with VNMRJ 3.2 software operating at 400.16 MHz equipped with a ASW probe at 293 K on 500 microliter samples at 20 C. 5/3 mm Precision NMR sample glass tube from Wilmad was used to avoid convection. The following are the parameters for the 1H NMR data acquisition; acquisition time 3.0 s, spectral width 6 kHz, pulse width 7.5 microseconds, relaxation delay 30 s, 16 scans. For 2D DOSY 1H NMR experiment, gradient compensated stimulated DOSY (DgcsteSL_cc) pulse sequence was used. The duration of the magnetic field pulse gradients was optimized for each diffusion time (t) in order to obtain 5-10% residual signal with maximum gradient strength. A pulse field gradient length (gt1) of 1.7 milliseconds and a diffusion delay (D) of 40 ms were used. For DOSY-NMR experiment, a series of 30 spectra on 32 K points were collected with the pulse gradients (g) increasing from 9.17 to 53.6 Gcm^{-1} in a linear ramp. All data were processed using Mnova software using Mnova software using the peak heights fit. After a two-dimensional Fourier transformation in magnitude mode, the DOSY dimension was reconstructed on a logarithmic scale using a mono exponential fit to all resonances and the peaks were reconstructed. In the diffusion constant dimension on a

grid of 30 data points. DOSY spectra are presented with chemical shifts on the horizontal axis and diffusion coefficients on the vertical axis expressed in cm^2s^{-1}.

Nuclear Overhauser Effect Spectroscopy (NOESY) Experiments: All spectra were acquired on a Bruker 300 spectrometer with Topspin 2.1 software operating at 300.13 MHz equipped with a BBO smart probe at 293 K on approximately 600 microliter samples at 20 C. The following are the parameters for the 1H NMR data acquisition; acquisition time 0.3 seconds, spectral width 3.125 kHz, pulse width 12.5 microseconds, relaxation delay 2 s, 16 scans. For 2D NOESY, a mixing time of 500 milliseconds was used with 256 points taken. All data were processed using Mnova software.

Spectroscopic Characterization

Steady State Absorption and Emission

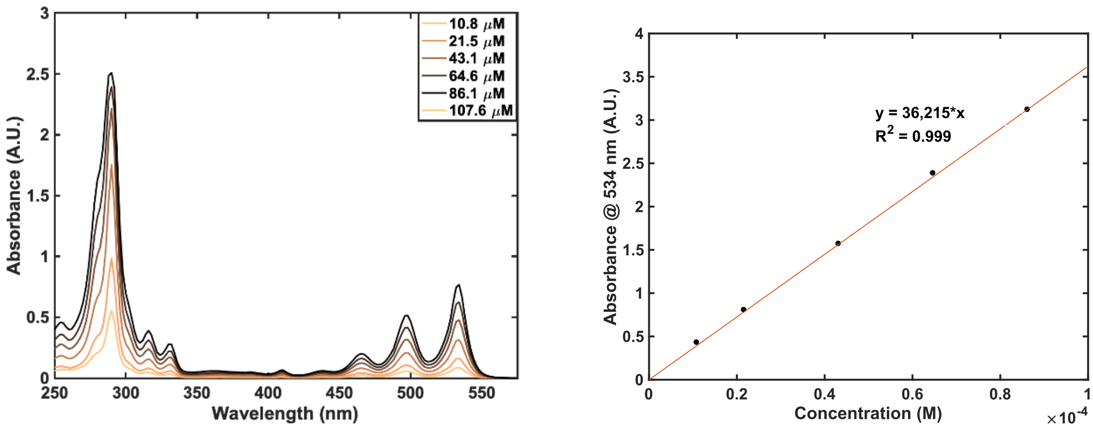

Figure S1. Left) The room temperature steady-state absorbance of **TIPS-Tc** in THF as a function of concentration taken in a 2 mm pathlength quartz cuvette. **Right**) The absorbance, scaled to a 1 cm pathlength, at 534 nm as a function of concentration with a linear fit where the slope corresponds to the molar absorption coefficient.

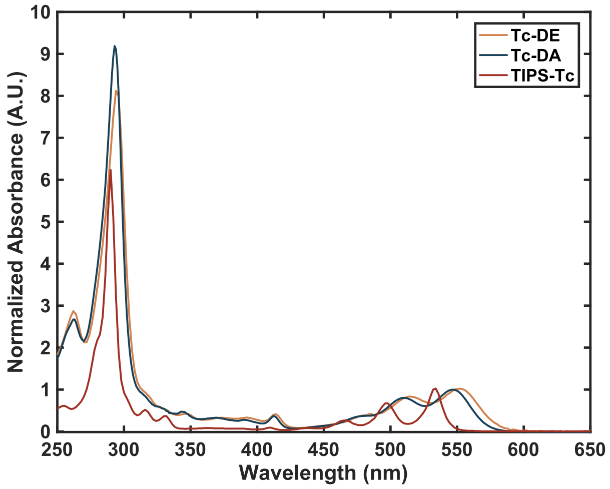

Figure S2. Comparison of the absorbance spectra of 20 μM **TIPS-Tc**, **Tc-DA**, and **Tc-DE** in THF normalized to their corresponding lowest energy 0-0* transition.

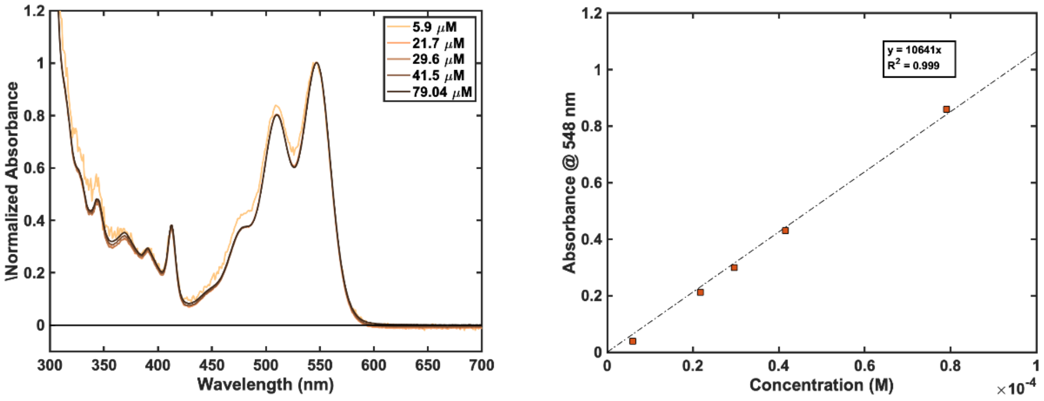

Figure S3. Left) The room temperature steady-state absorbance profile of **Tc-DA** in THF taken in a 1 cm pathlength quartz cuvette. The data has been normalized to the 0-0* transition at each concentration to demonstrate a concentration independent absorbance profile. **Right**) The absorbance at 548 nm as a function of concentration with a linear fit where the slope corresponds to the molar absorption coefficient.

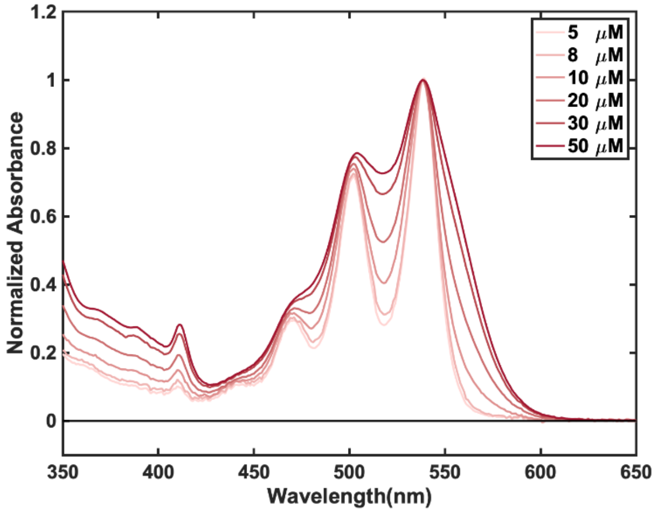

Figure S4. The room temperature steady-state absorbance profile of **Tc-DA** in DMSO taken in a 1 cm pathlength quartz cuvette. The data has been normalized to the 0-0* transition for each concentration to demonstrate the change in absorbance profile as a function of concentration.

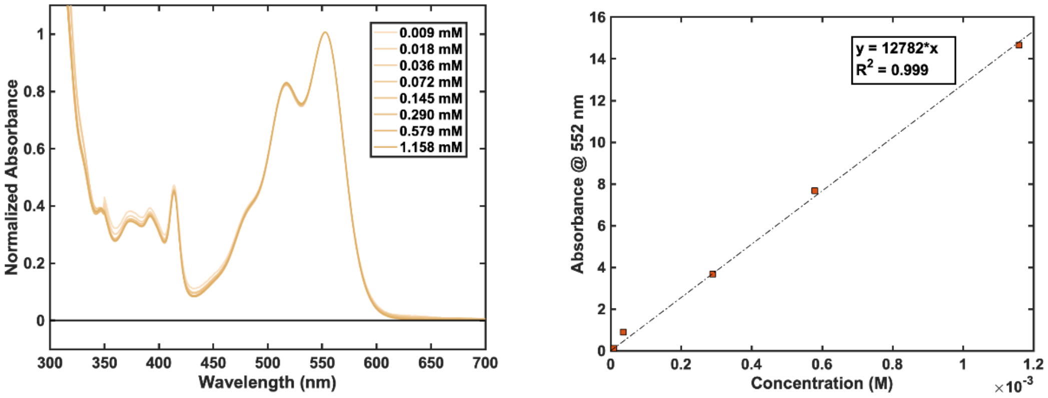

Figure S5. Left) The room temperature steady-state absorbance profile of **Tc-DE** in DMF taken in a 1 mm pathlength quartz cuvette. The data has been normalized to the 0-0* transition for each concentration to demonstrate a concentration independent absorbance profile. **Right**) The absorbance at 552 nm scaled to a 1 cm pathlength as a function of concentration with a linear fit where the slope corresponds to the molar absorption coefficient.

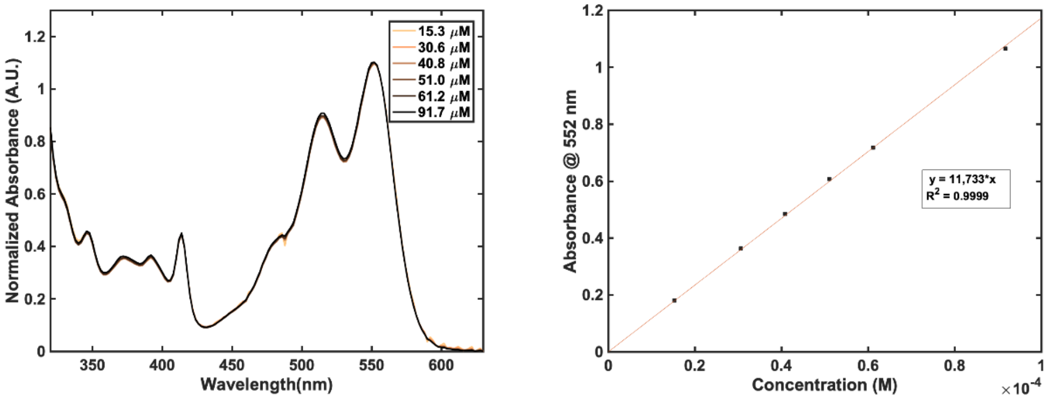

Figure S6. Left) The room temperature steady-state absorption profile of **Tc-DE** in THF taken in a 1 cm pathlength quartz cuvette. The data has been normalized to the 0-0* transition for each concentration to demonstrate the change in absorption profile as concentration is increased. **Right**) The absorbance at 552 nm as a function of concentration with a linear fit where the slope corresponds to the molar absorption coefficient.

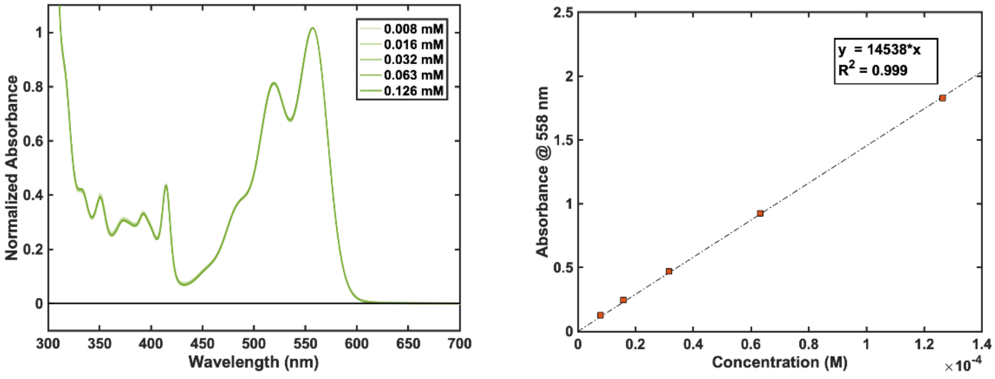

Figure S7. Left) The room temperature steady-state absorbance profile of **Tc-DE** in chloroform taken in a 1 cm pathlength quartz cuvette. The data has been normalized to the 0-0* transition for each concentration to demonstrate the change in absorbance profile as concentration is increased. **Right**) The absorbance at 558 nm as a function of concentration with a linear fit where the slope corresponds to the molar absorption coefficient.

Solvent Dependent Steady State Absorption and Emission

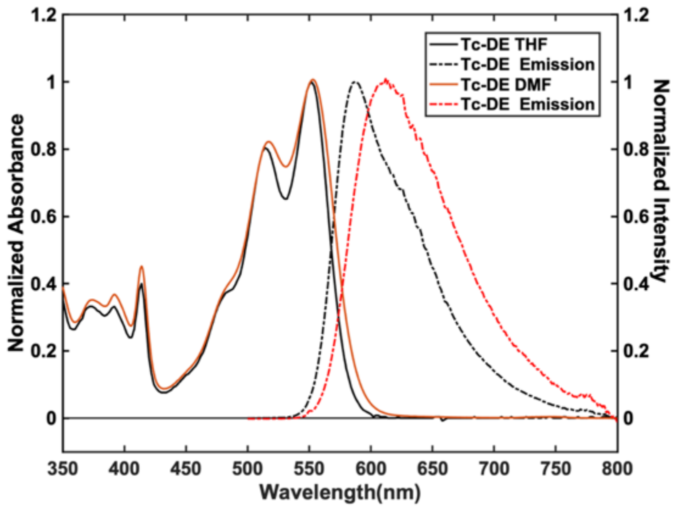

Figure S8. Normalized absorbance and emission spectra of **Tc-DE** in DMF (red) and THF (black). Each sample was prepared under an argon atmosphere then excited with 550 nm light and collected with 0.5 s integration time.

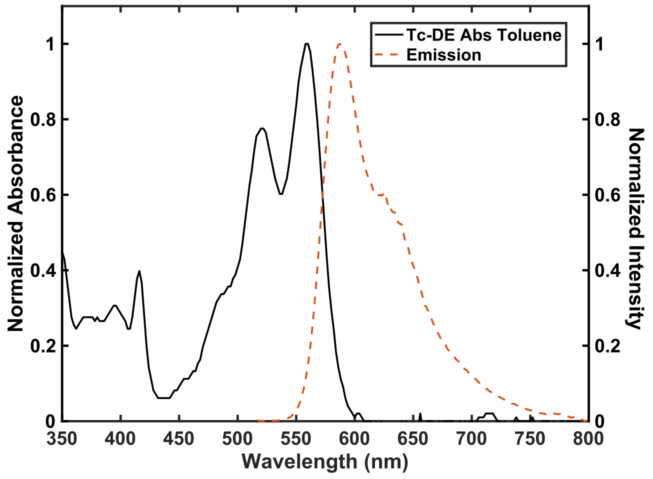

Figure S9. Normalized absorbance (black solid) and emission spectra (red dashed) of **Tc-DE** in toluene. The sample was prepared under an argon atmosphere then excited with 550 nm light and collected with 0.5 s integration time. Tc-DE was found to have an 81% QY and emissive lifetime of approximately 15.1 ns.

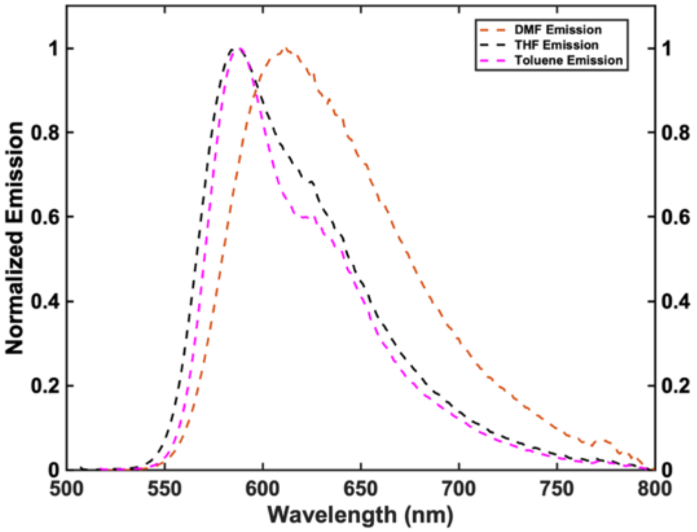

Figure S10. Normalized fluorescence spectra of **Tc-DE** in DMF (red), THF (black), and toluene (magenta). Each sample was prepared under an argon atmosphere then excited with 550 nm light and collected with 0.5 s integration.

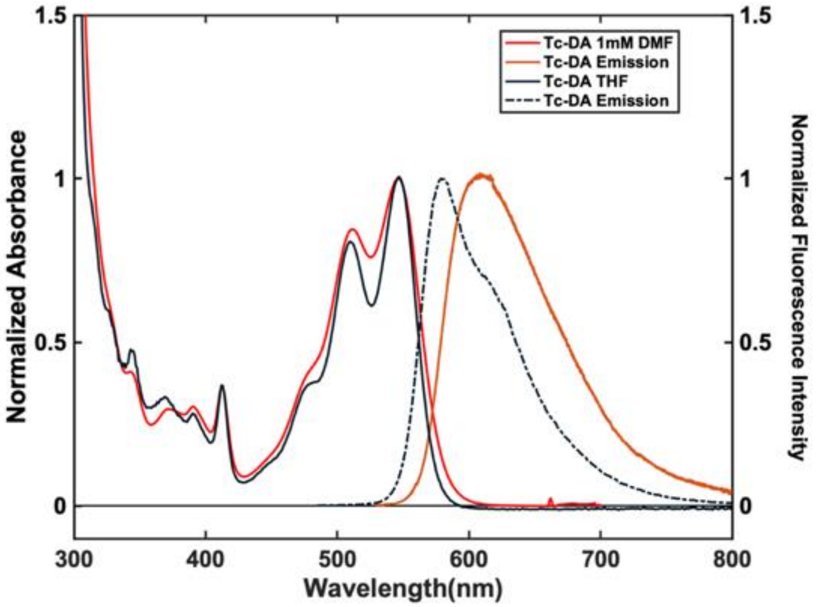

Figure S11. Normalized absorbance and emission spectra of 1 mM **Tc-DA** in DMF (red) and 0.039 mM **Tc-DA** in THF (black). The absorbance profile of **Tc-DA** in THF, which is invariant of concentration (**Figure S3**), matches the 1 mM **Tc-DA** absorbance profile collected in DMF.

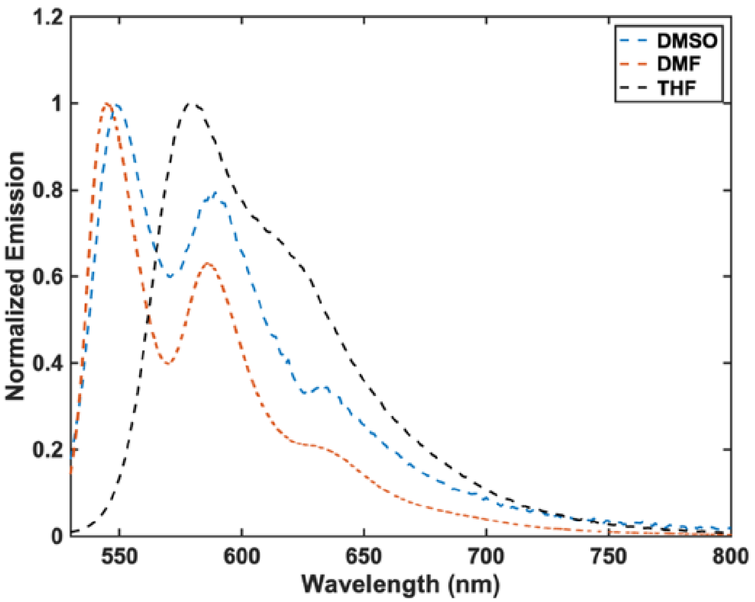

Figure S12. Normalized fluorescence spectra of **Tc-DA:** 10 μM in DMF (red), 35 μM in THF (black), and 15 μM in DMSO (light blue). Each sample was prepared under an argon atmosphere then excited with 500 nm light and collected with 0.5 s integration.

Steady-state absorption global analysis

Turning to concentration-dependent absorption, a commonly used probe of structure in molecular aggregates, it is tempting to assign the significant red shift in the absorption spectrum in DMF to overall stronger coupling, but here it is important to consider the various factors that lead to spectral shifts and change in envelope functions in aggregates. The spectral profiles can be roughly described by a model of Coulomb and charge-transfer coupling.[15] In the purely coulombic Kasha theory of aggregates, we would assign the strongly redshifted species as a J-aggregate. However, although the spectral profile is redshifted, the 0-0* feature is not enhanced relative to the 0-1*. In photoluminescence, the spectral profile adopts a broad, excimer-like structure that is strongly quenched relative to the monomer. Both of these behaviors oppose classifying the species as a J-aggregate. Further, DFT geometry optimizations find that the aggregate species prefer to adopt an almost perfectly π-stacked geometry, suggesting an H-aggregate. The inconsistent spectral markers imply that aggregate behavior in **Tc-DA** is not purely defined by coulombic interactions. Thus, we turn to Spano's expansion of Kasha aggregate theory, which integrates CT and exciton interactions. There, chromophores separated by small intermolecular distances (e.g. 3-4 Å separated π-stacks) can exhibit aggregate behavior highly divergent from classic Kasha aggregates due to substantial wavefunction overlap. The effect is further pronounced in molecules such as tetracene where the S_0->S_1 transition is aligned along the short axis, resulting in weak coulombic coupling. Due to the strong π-stacking in **Tc-DA** aggregates and the mix of J and H spectral signatures observed, we propose that the aggregate species exhibit nearly Hj null behavior.[2] To further extract information about the species present vs. concentration, we begin by simultaneously fitting concentration dependent absorbance spectra of **Tc-DA** to a Franck-Condon vibronic progression (**Figure S12**). Although the lowest and highest concentration spectra are well-described by this model, the intermediate regime (0.257 mM) data are not. We then used a linear combination of the highest and lowest concentration Franck-Condon progression fits as bases in a global analysis which attempted to reproduce each spectrum in the concentration series. This approach again succeeded in matching the monomer and aggregated regimes but failed to describe the intermediate. By plotting the relative amplitudes of the spectral basis functions against concentration, we observe a clear

aggregation isotherm **(Figure 2A, inset).** Manually fitting this isotherm to the nucleation-elongation model allows us to extract a cooperativity coefficient and equilibrium constants.[16,17] From the nucleation-elongation model we confirm that the aggregation of **Tc-DA** in DMF is highly cooperative, agreeing with the onset of aggregation at very low concentration. The failure of the fitting routine to properly describe the data in the intermediate concentration regime implies involvement of a distinct aggregate species. Averaging the residuals of the global fit in the intermediate regime returns a vibronic progression that is distinct from either the low or high concentration absorbance spectra. This slightly red-shifted and broadened profile compared to the monomer roughly matches that of the calculated dimer spectrum in **Figure 7a**. The continued broadening and large red shift indicate further growth or evolution of the aggregates.

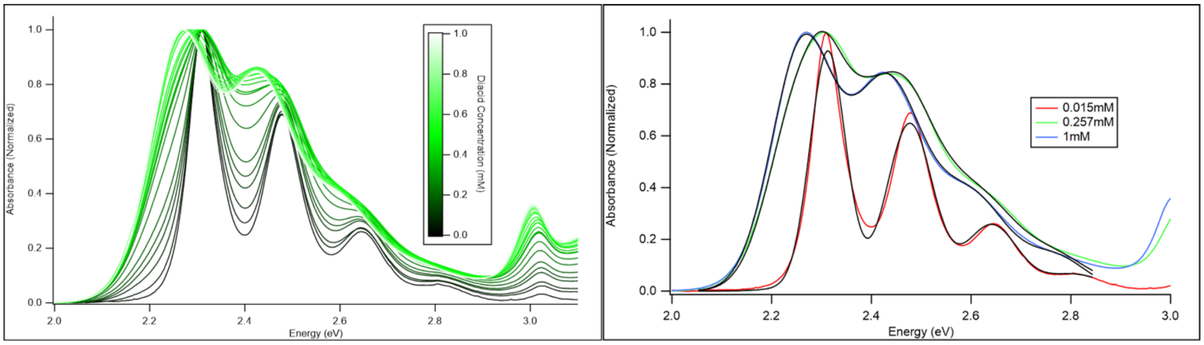

Figure S13. **Left**) Linear absorbance concentration series of **Tc-DA** in DMF. **Right**) Linear absorbance global fit results visualized (black) against representative absorption spectra for each regime.

Time Correlated Single Photon Counting (TCSPC)

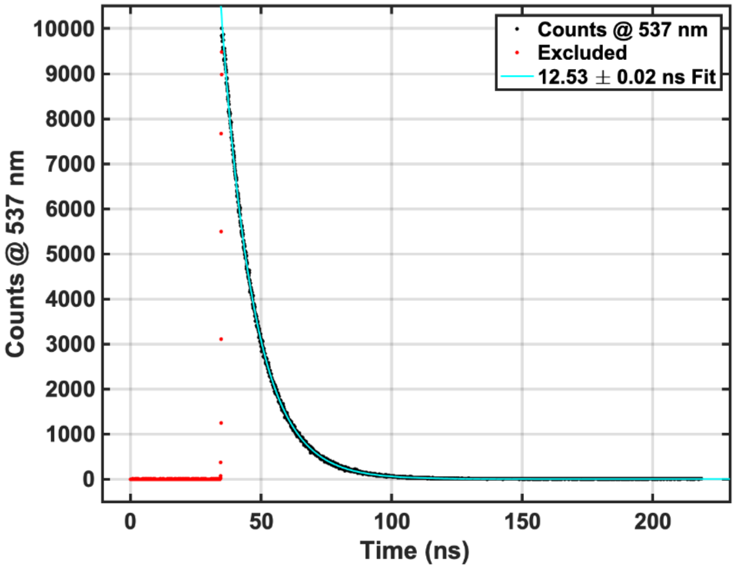

Figure S14. TCSPC decay at 537 nm (black dots) and fit (solid line) for **TIPS-Tc** in THF.

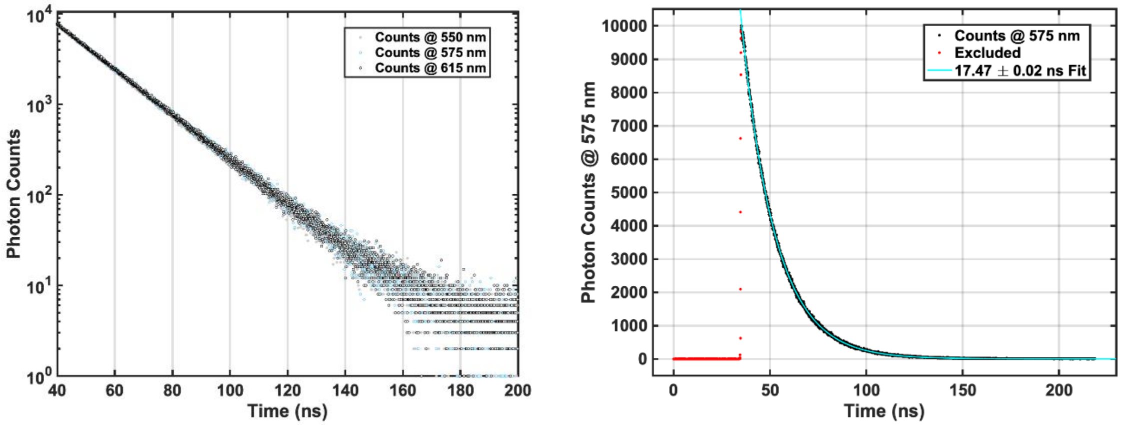

Figure S15. Left) TCSPC decay at multiple wavelengths (see legend) for **Tc-DA** in THF. **Right)** TCSPC decay at 575 nm (black dots) and fit (solid line) for **Tc-DA** in THF.

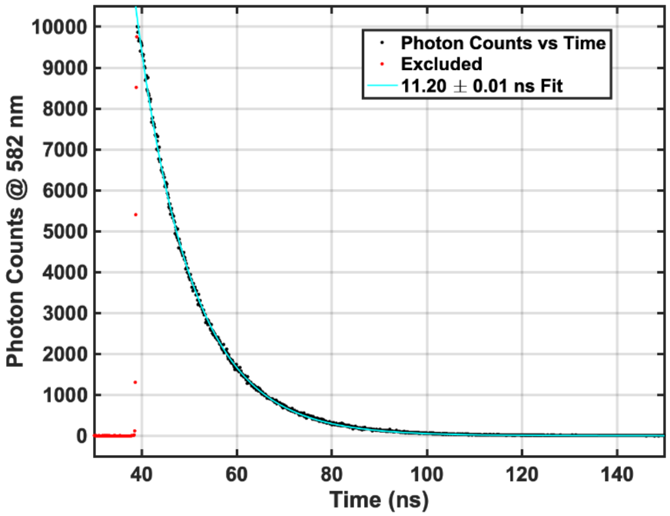

Figure S16. TCSPC decay at 582 nm (black dots) and fit (solid line) for **Tc-DE** in THF.

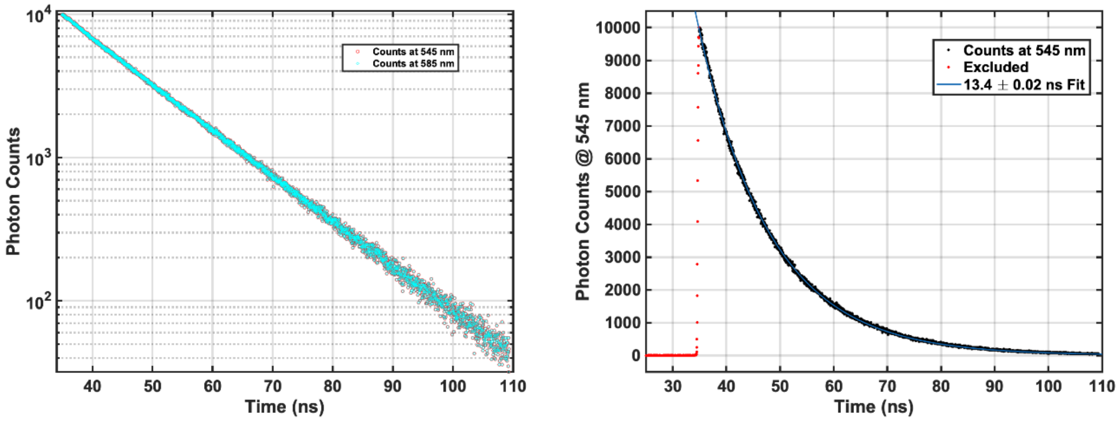

Figure S17. Left) TCSPC decay at 545 and 585 nm for 10 μM **Tc-DA** in DMF. **Right)** TCSPC decay at 545 nm (black dots) and fit (solid line) for 10 μM **Tc-DA** in DMF.

Time Resolved Photoluminescence

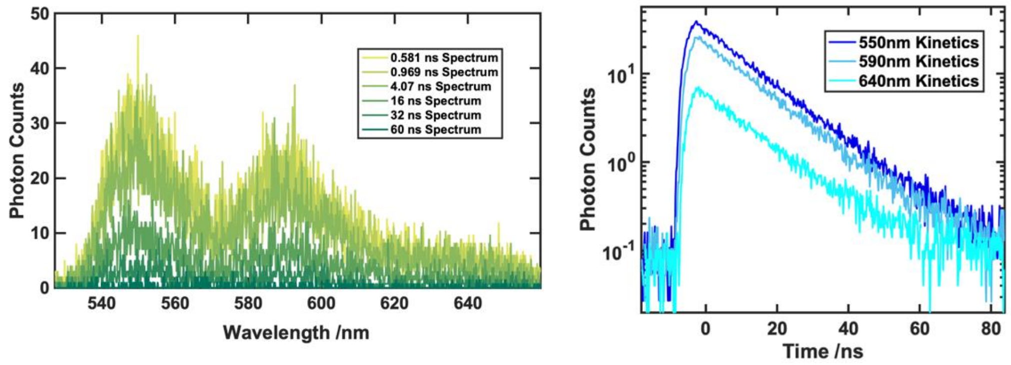

Figure S18. Left) Time-resolved photoluminescence spectral slices and **Right**) select kinetic traces of 0.01 mM **Tc-DA** in DMF after excitation at 505 nm at room temperature.

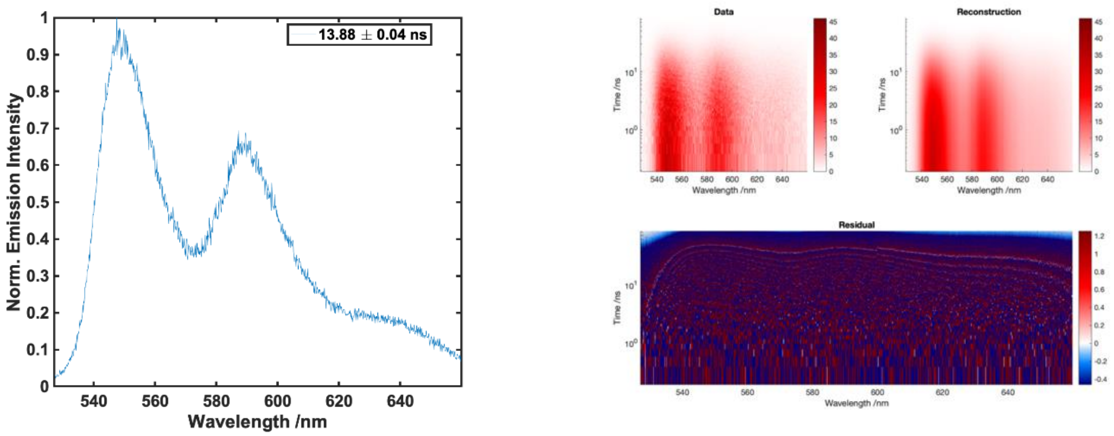

Figure S19. Left) Decay associated spectrum obtained through global analysis of the time-resolved photoluminescence of 0.01 mM **Tc-DA** in DMF shown in **Fig. S18**. A single component with a lifetime of 13.9 ± 0.04 ns was used to fit this data. **Right**) The raw TRPL data (left) collected for 0.01 mM **Tc-DA** in DMF along with a reconstructed model (right) composed of an A $\rightarrow$ 0 process.

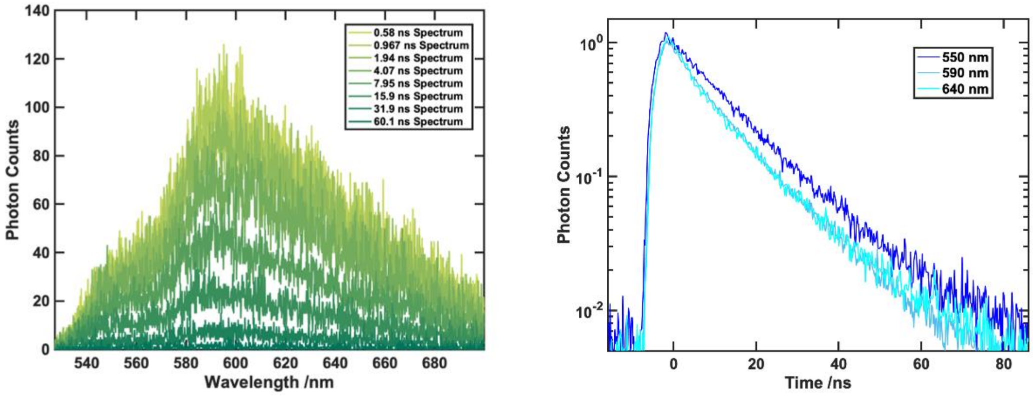

Figure S20. Left) Time-resolved photoluminescence spectral slices and **Right**) select normalized kinetic traces of 0.30 mM **Tc-DA** in DMF after excitation at 505 nm at room temperature.

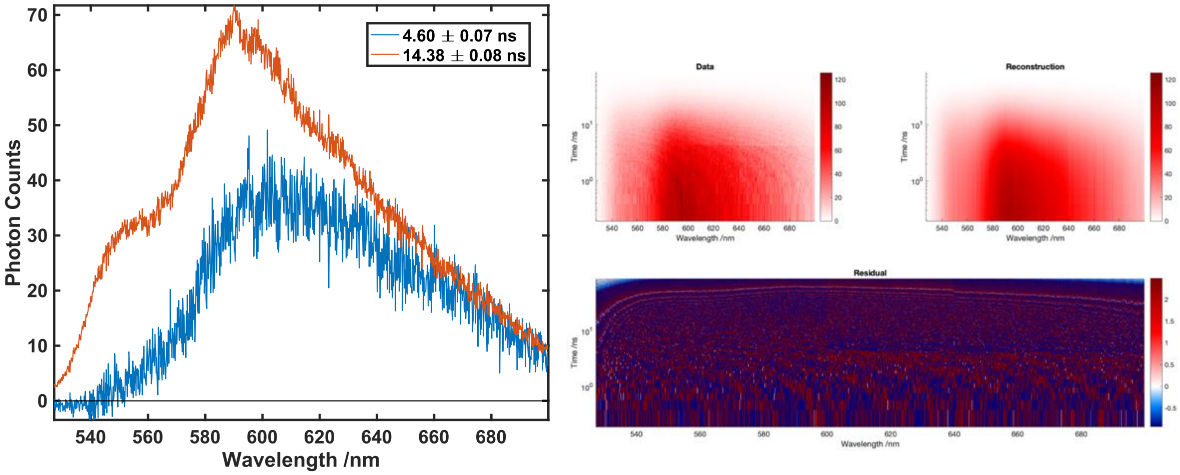

Figure S21. Left) Decay associated spectra obtained through global analysis of the time-resolved photoluminescence of 0.30 mM **Tc-DA** in DMF shown in **Fig. S20**. Two components with a lifetime of 4.60 ± 0.07 ns and 14.4 ± 0.08 ns were used to fit this data. **Right**) The raw TRPL data (left) collected for 0.30 mM **Tc-DA** in DMF along with a reconstructed model (right) composed of an $A \rightarrow 0$, $B \rightarrow 0$ process.

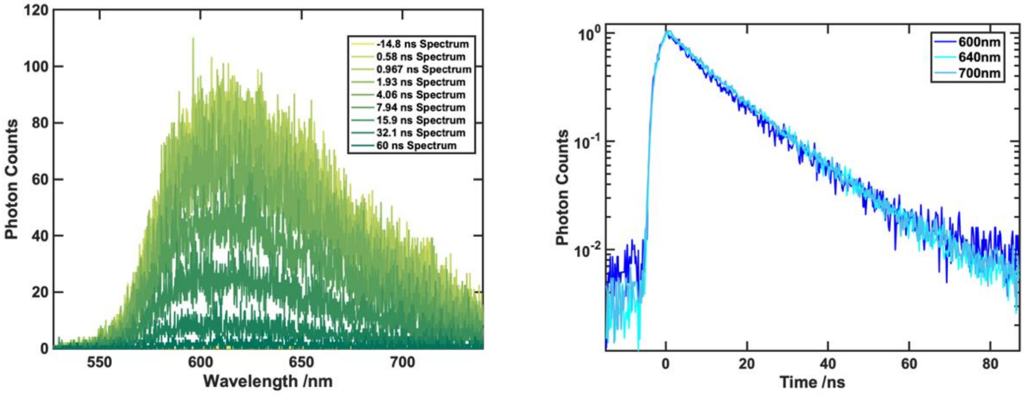

Figure S22. Time-resolved photoluminescence spectral slices and select normalized kinetic traces of 1.0 mM **Tc-DA** in DMF after excitation at 505 nm at room temperature.

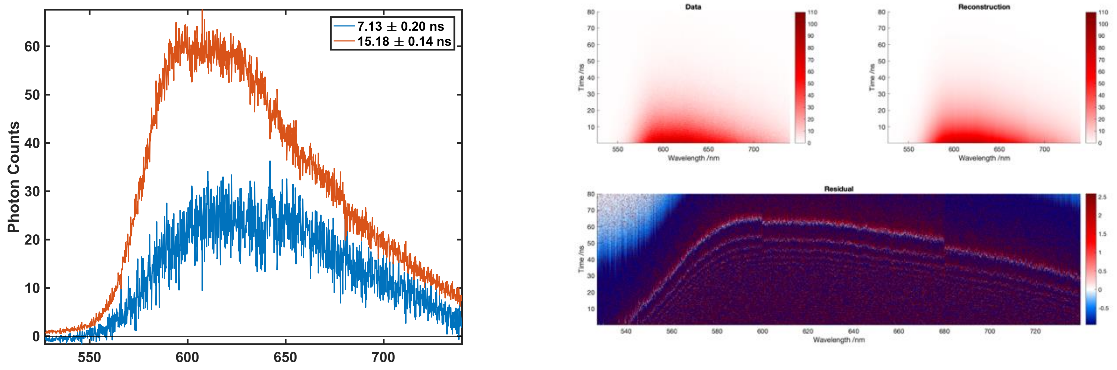

Figure S23. Left) Decay associated spectra obtained through global analysis of the time-resolved photoluminescence of 1.0 mM **Tc-DA** in DMF shown in **Fig. S22**. Two components with a lifetime of 7.1 ± 0.2 ns and 15.2 ± 0.14 ns were used to fit this data. **Right**) The raw TRPL data (left) collected for 1.0 mM **Tc-DA** in DMF along with a reconstructed model (right) composed of an $A \rightarrow 0$, $B \rightarrow 0$ process.

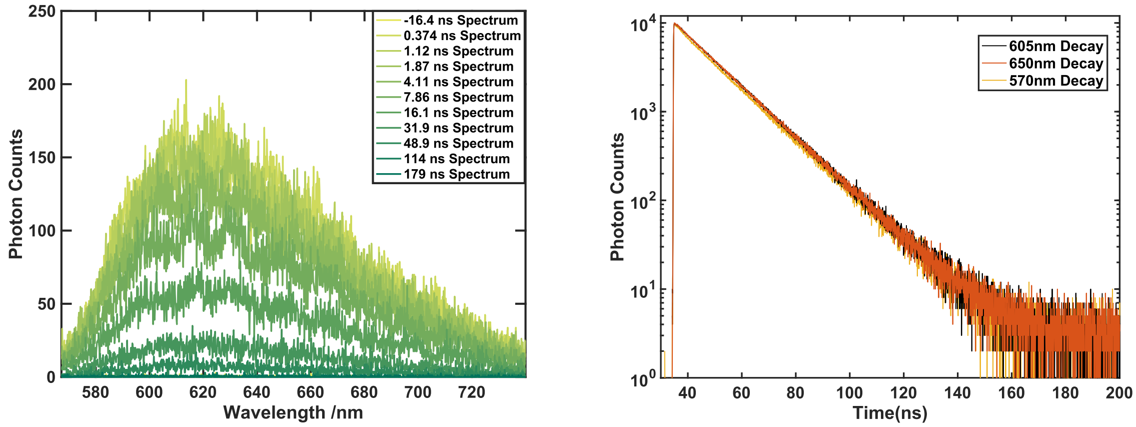

Figure S24. Left) Time-resolved photoluminescence spectral slices and **Right**) select single wavelength decay traces of approximately 20 μM **Tc-DE** in DMF after excitation at 532 nm at room temperature.

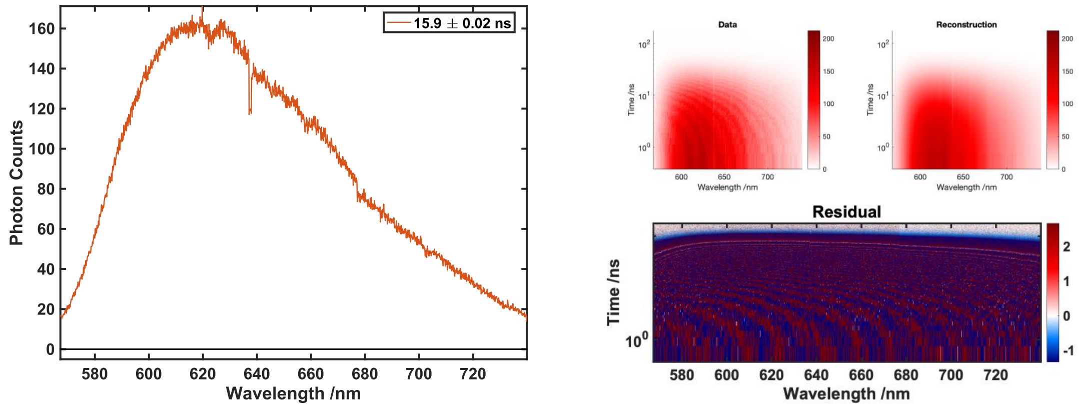

Figure S25. Left) Decay associated spectrum obtained through global analysis of the time-resolved photoluminescence of 20 μM **Tc-DE** in DMF shown in **Fig. S24**. The data is well fit by a single component with a lifetime of 15.9 ± 0.02 ns. **Right**) The raw TRPL data (left) collected for 20 μM **Tc-DE** in DMF along with a reconstructed model (right) composed of an A → 0 process.

Transient Absorption

TIPS-Tc in THF

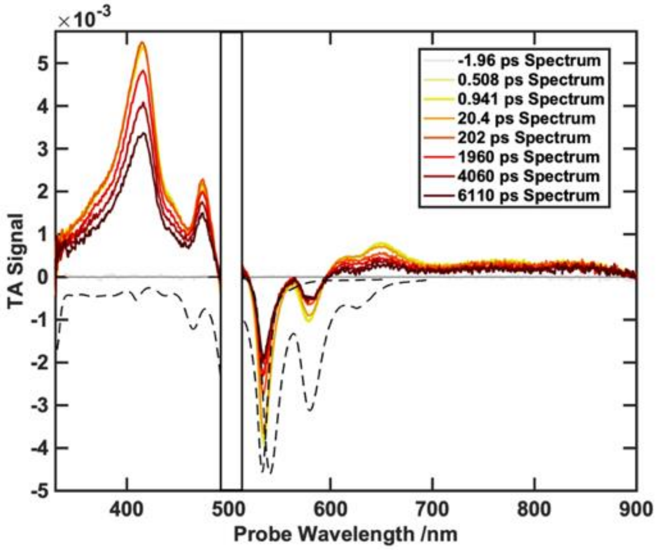

Figure S26. Spectral slices from femtosecond transient absorption experiment of 25 μM **TIPS-Tc** in THF after excitation at 500 nm with 55 μW.

Tc-DA: 0.015 mM in DMF

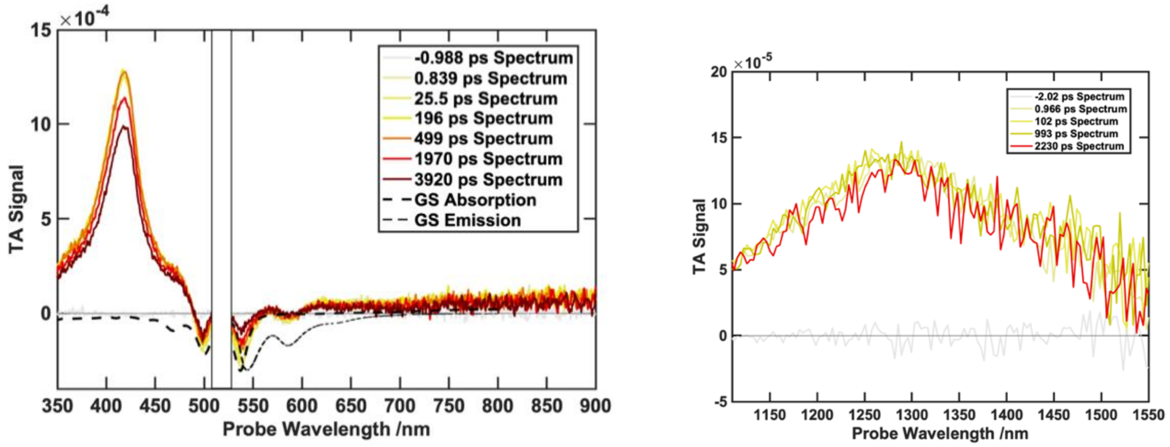

Figure S27. Near-UV, Visible (**Left**) and NIR (**Right**) spectral slices from femtosecond transient absorption performed on 0.015 mM **Tc-DA** in DMF up to 5 ns after excitation at 520 nm with 55 μW.

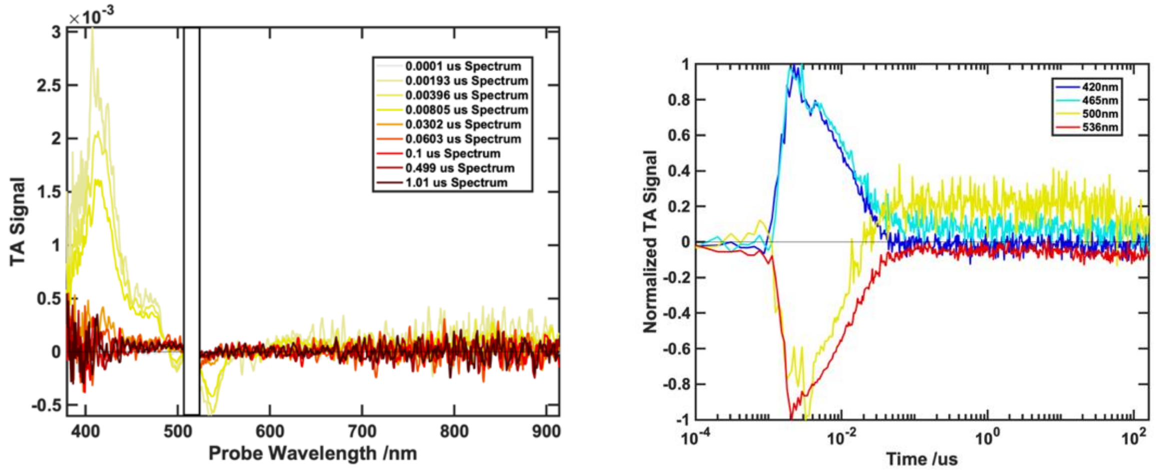

Figure S28. **Left**) Spectral slices from a nanosecond transient absorption experiment performed on 0.015 mM **Tc-DA** in DMF up to 180 μs after excitation at 520 nm with 55 μW. **Right**) Normalized kinetics from the nanosecond transient absorption experiment performed on 0.015 mM **Tc-DA** in DMF.

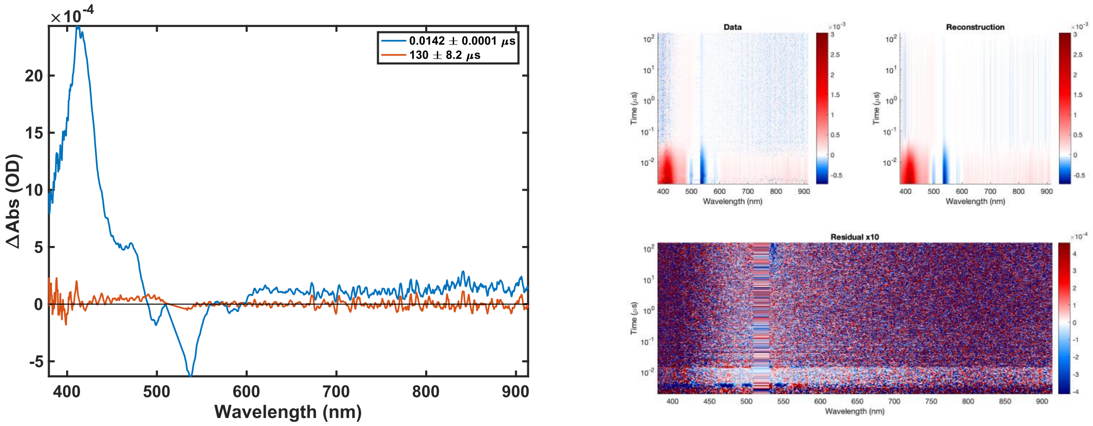

Fig S29. **Left**) Two-state species associated spectra retrieved from global analysis of the nanosecond transient absorption of 0.015 mM **Tc-DA** in DMF. A sequential model of A → B → 0 was applied. The shorter component (14.2 ns) is associated with the singlet excited state (S_1) of **Tc-DA**. The longer component (130 μs) is associated with the first triplet excited state (T_1) of **Tc-DA** that is formed in a small percentage through intersystem crossing from S_1. **Right**) The raw nsTA data (left) collected for 0.015 mM **Tc-DA** in DMF along with a reconstructed model (right) composed of an A → B → 0 process.

Tc-DA: 0.25~~0~~ mM in DMF

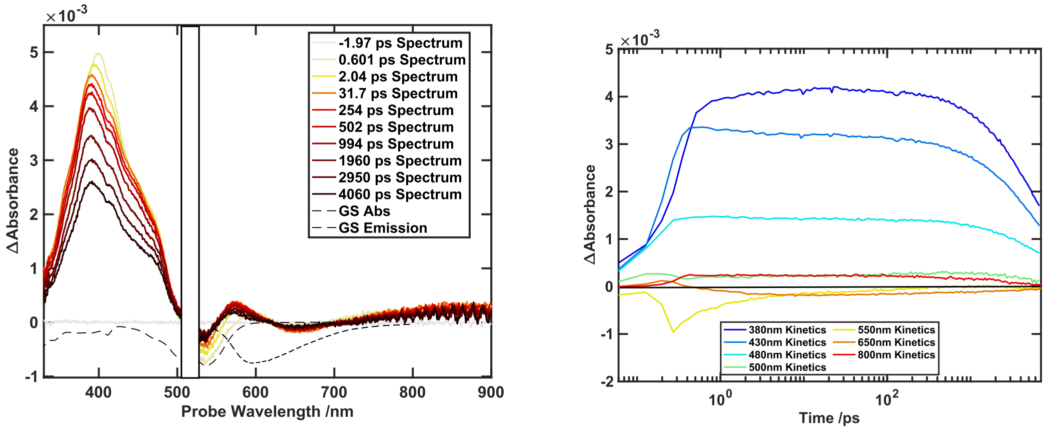

Figure S30. **Left**) Spectral slices and **Right**) select kinetics from a femtosecond transient absorption experiment performed on 0.25 mM **Tc-DA** in DMF up to 5 nanoseconds after 520 nm excitation with 55 μW.

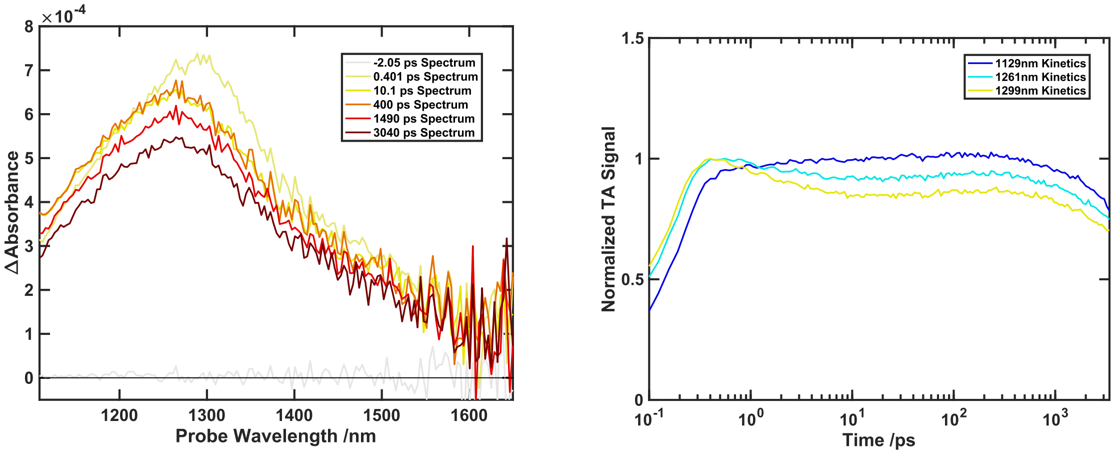

Figure S31. **Left**) NIR transient absorption spectral slices and **Right**) select single wavelength kinetics of 0.25 mM **Tc-DA** in DMF up to 5 ns after excitation at 520 nm with 55 μW.

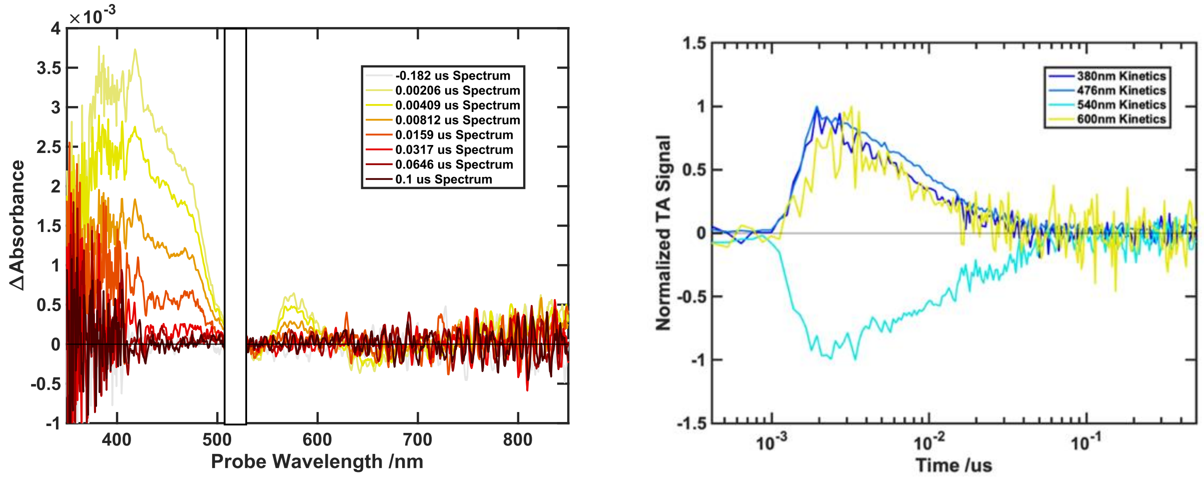

Figure S32. **Left**) Spectral slices from a nanosecond transient absorption experiment performed on 0.25 mM **Tc-DA** in DMF up to 100 ns after excitation at 520 nm with 55 μW. **Right**) Select kinetics from the nanosecond transient absorption experiment performed on 0.25 mM **Tc-DA** in DMF.

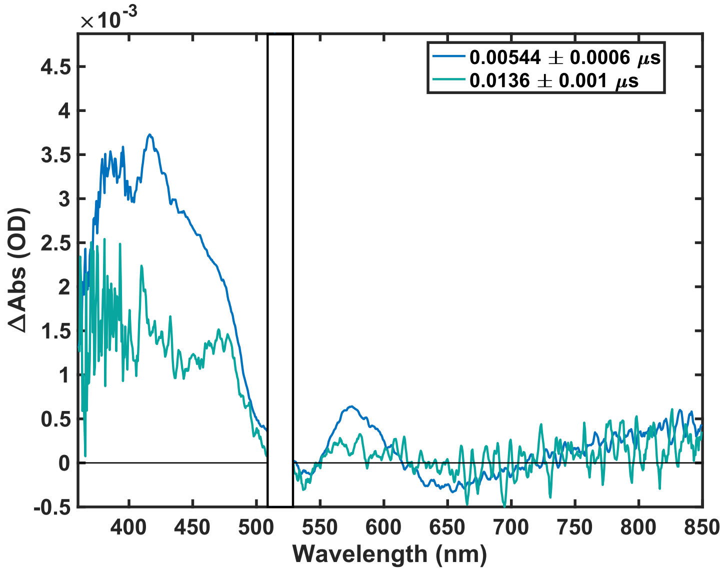

Figure S33. Two-state species associated spectra retrieved from global analysis of the nanosecond transient absorption of 0.25 mM **Tc-DA** in DMF. A sequential model of A → 0, B → 0 was applied.

<u>Tc-DA</u>: 1.0 mM in DMF

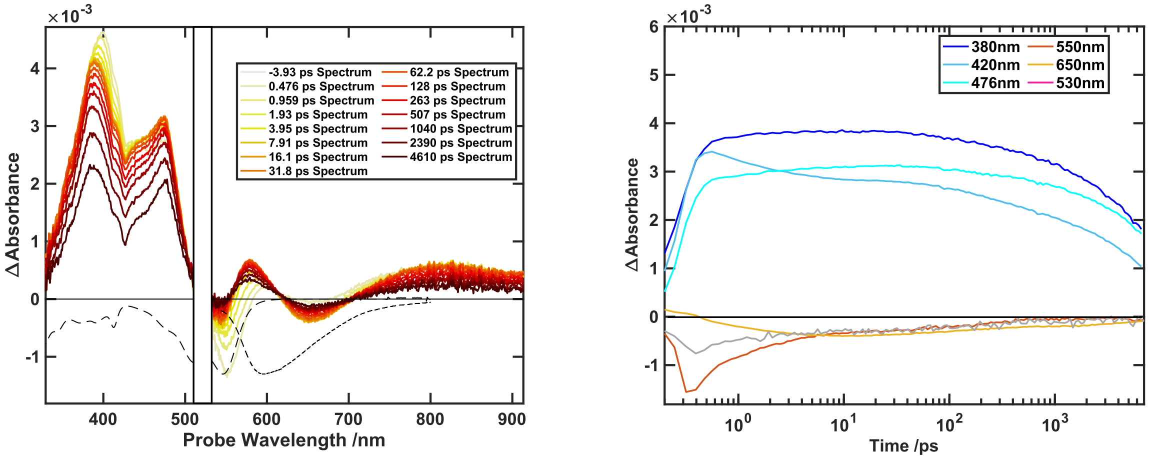

Figure S34. **Left**) Spectral slices from a femtosecond transient absorption experiment performed on 1.0 mM **Tc-DA** in DMF up 5 ns after excitation at 520 nm with 55 μW. **Right**) Select kinetics from the femtosecond transient absorption experiment performed on 1.0 mM **Tc-DA** in DMF.

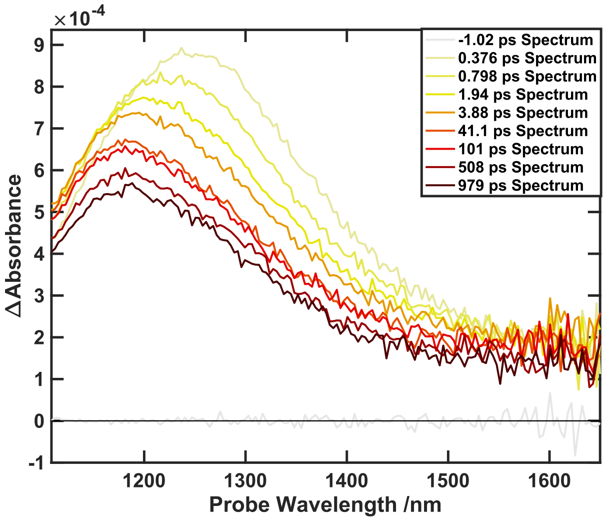

Figure S35. NIR femtosecond transient absorption spectral slices of 1.0 mM **Tc-DA** in DMF after excitation at 520 nm with 55 μW.

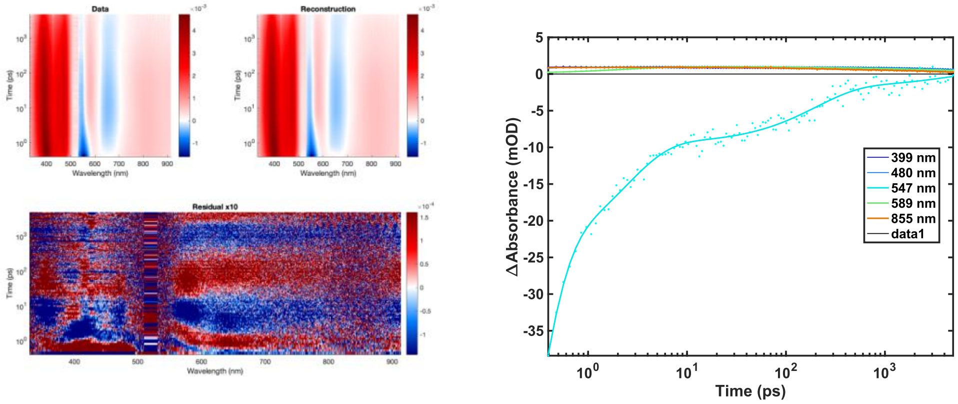

Figure S36. **Left**) The raw fsTA data (upper left) collected for 1.0 mM **Tc-DA** in DMF along with a reconstructed model (upper right) composed of an A → C → D → 0; B → E → 0 process. **Right**) Single wavelength kinetics and their corresponding fits from the applied model.

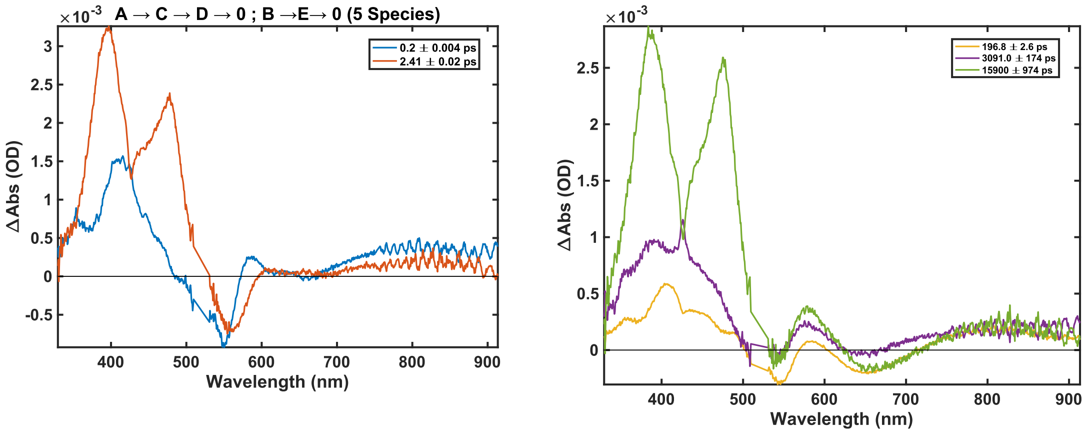

Figure S37. Species associated spectra (SAS) extracted from global analysis of the 1.0 mM **Tc-DA** fsTA experiment. A five state model of A → C → D → 0; B → E → 0 was applied to the components and separated into two figures to reduce visual clutter. **Left**) The two fastest SAS corresponding to A and B which are the initially formed states in two separate aggregate populations. **Right**) The three slower SAS corresponding to C, D and E. C, is the initially formed excimer state from population A that then undergoes a relaxation process with a lifetime of 196.8 ps into D, assigned as the relaxed excimer, which decays back to the ground state with a lifetime of 3091 ps. E, formed in 2.41 ps from the initial excited state population A, is assigned to the TT+CT mixed state which decays with a lifetime of 15900 ps.

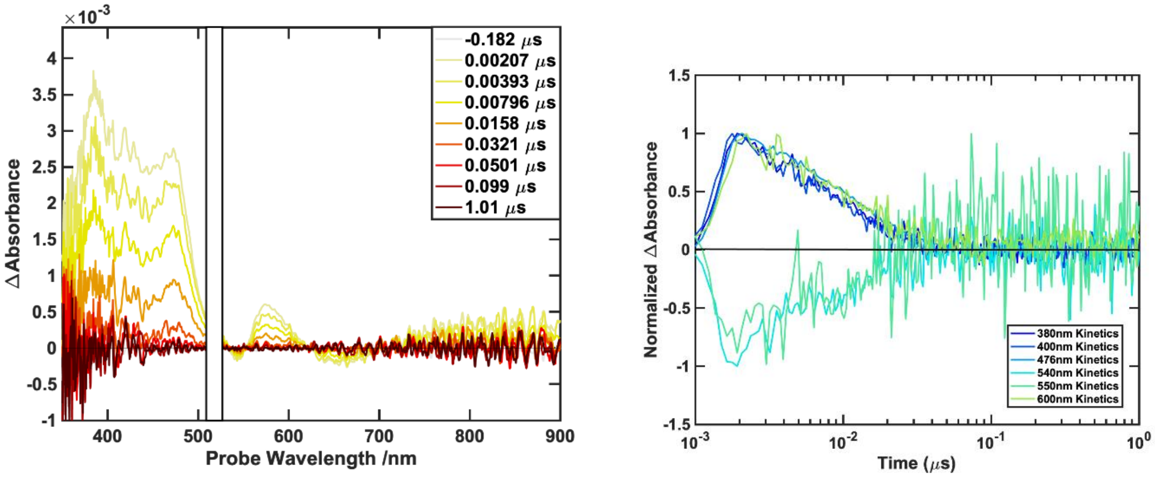

Figure S38. **Left**) Nanosecond transient absorption spectral slices up to 1 μs and **Right**) select normalized kinetics of 1.0 mM **Tc-DA** in DMF after excitation at 520 nm with 55 μW.

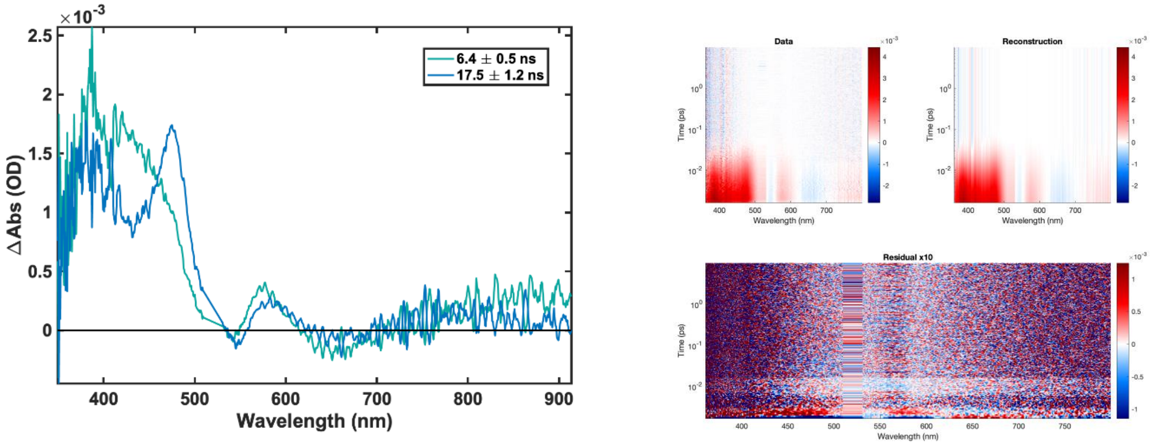

Figure S39. Left) Two-state species associated spectra retrieved from global analysis of the nanosecond transient absorption of 1.0 mM **Tc-DA** in DMF. A parallel model of A → 0; B → 0 was applied. The shorter component is associated with the excimer excited state of **Tc-DA**. The longer component is associated with the mixed CT+TT of **Tc-DA**. **Right**) The raw nsTA data (upper left) collected for 1.0 mM **Tc-DA** in DMF along with a reconstructed model (upper right) composed of an A → 0 ; B → 0 process.

__**Tc-DE**: 0.11 mM in DMF__

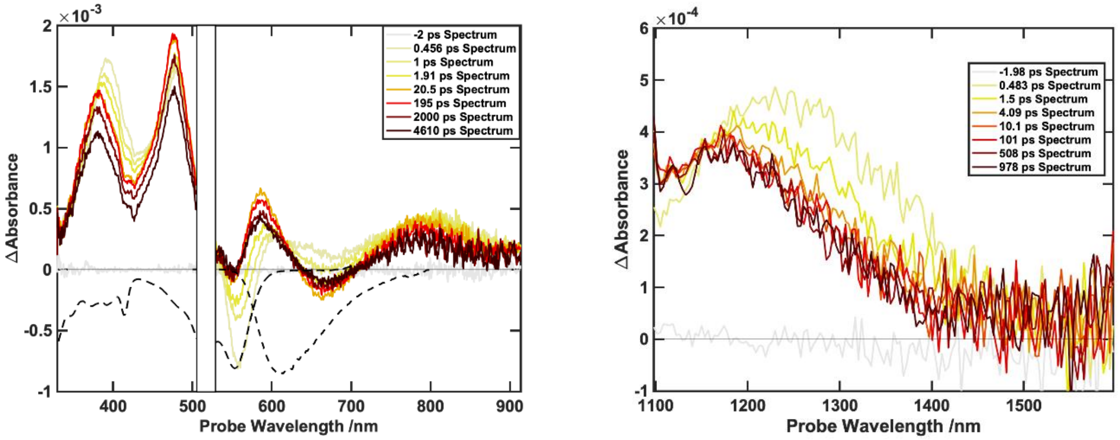

Figure S40. Spectral slices from femtosecond transient absorption experiment performed on 0.11 mM **Tc-DE** in DMF after excitation at 520 nm with 55 μW.

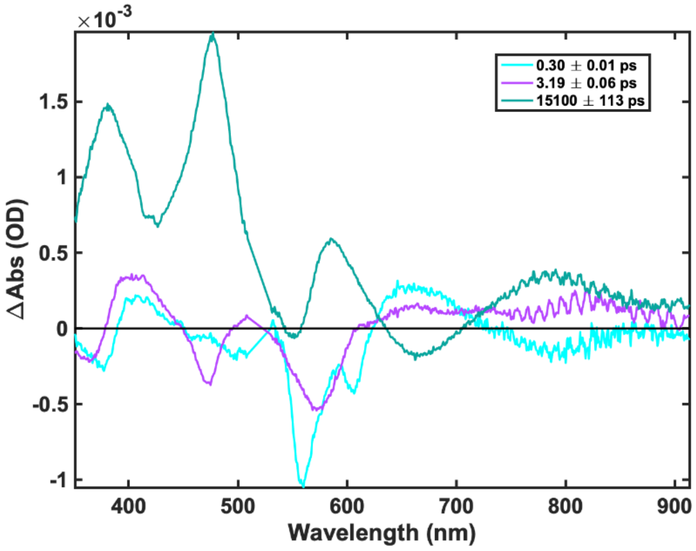

Figure S41. Decay associated spectra extracted through a three-component global analysis from femtosecond transient experiment absorption performed on 0.11 M **Tc-DE** in DMF.

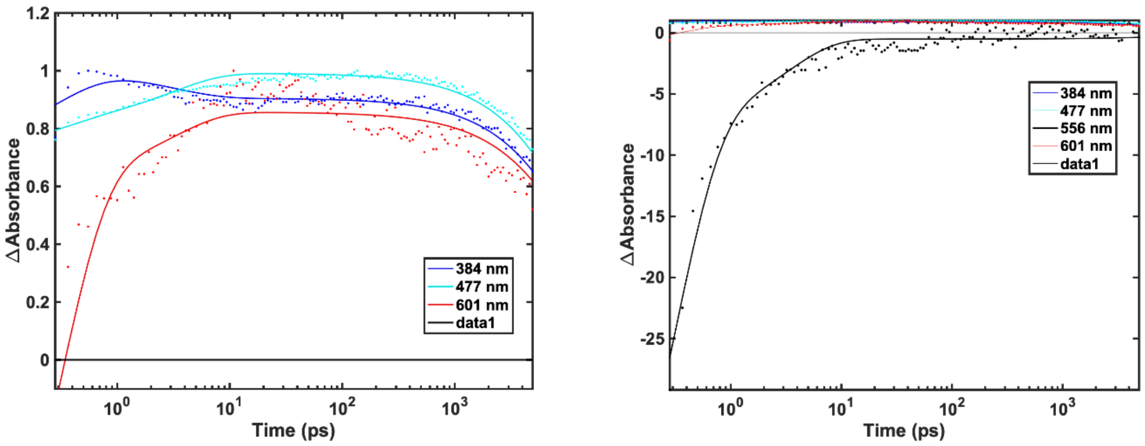

Figure S42. The raw data (dots) and respective fits (solid lines) from a three-component model to select single wavelength kinetics from the femtosecond transient absorption experiment performed on 0.11 mM **Tc-DE** in DMF.

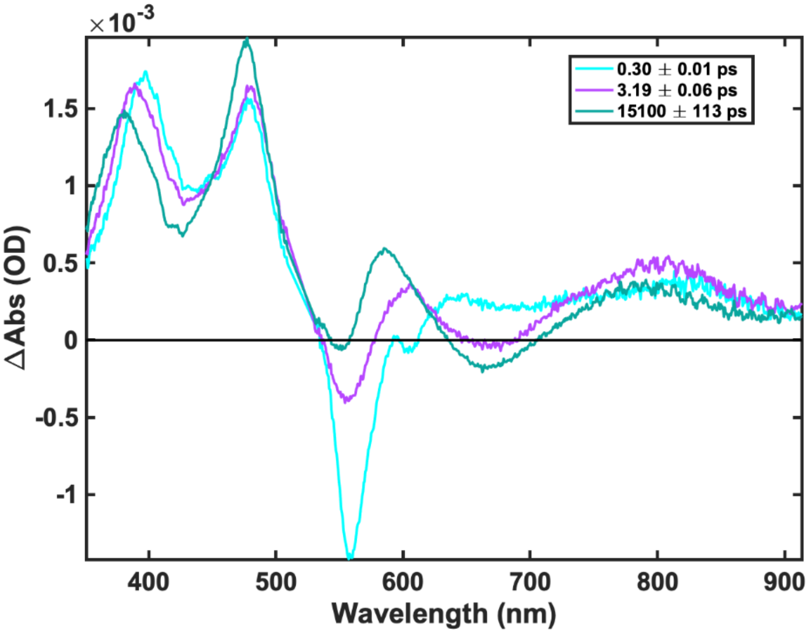

Figure S43. Species associated spectra extracted from global analysis of fsTA performed on 0.11 mM **Tc-DE** in DMF and application of linear three component model A $\rightarrow$ B $\rightarrow$ C $\rightarrow$ 0.

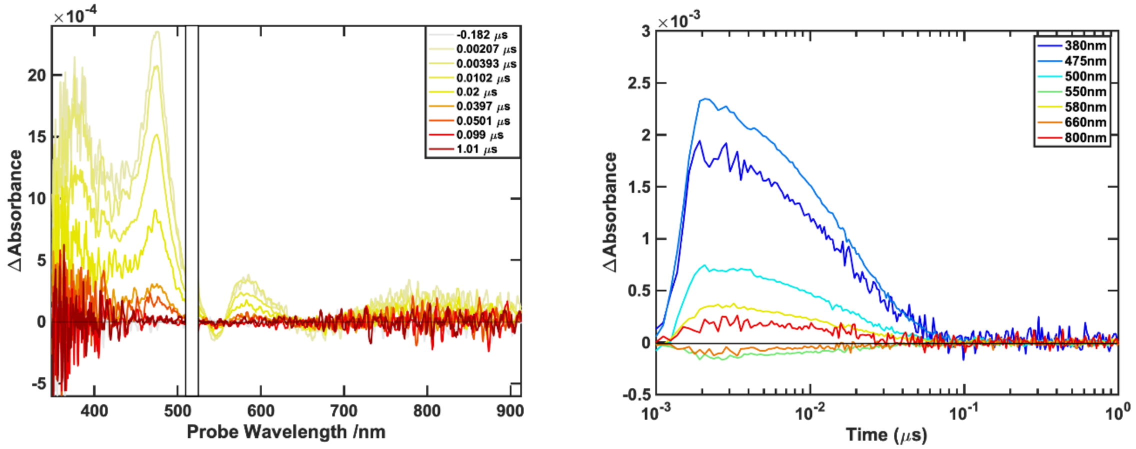

Figure S44. **Left**) Transient absorption spectral slices up to 500 nanoseconds and **Right**) select normalized kinetics of 0.11 mM **Tc-DE** in DMF after excitation at 520 nm with 55 μW.

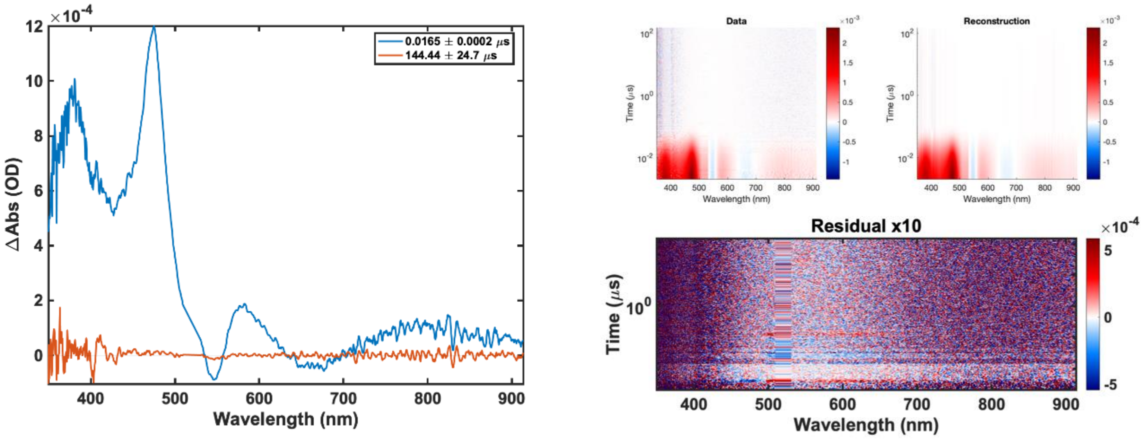

Figure S45. **Left**) Two-state species associated spectra retrieved from global analysis of the nanosecond transient absorption of 0.11 mM **Tc-DE** in DMF. A sequential model of A $\rightarrow$ B $\rightarrow$ 0 was applied. **Right**) The raw nsTA data (upper left) collected for 0.11 mM **Tc-DE** in DMF along with a reconstructed model (upper right) composed of an A $\rightarrow$ B $\rightarrow$ 0 process.

__**Tc-DE**: 1.0 mM in DMF__

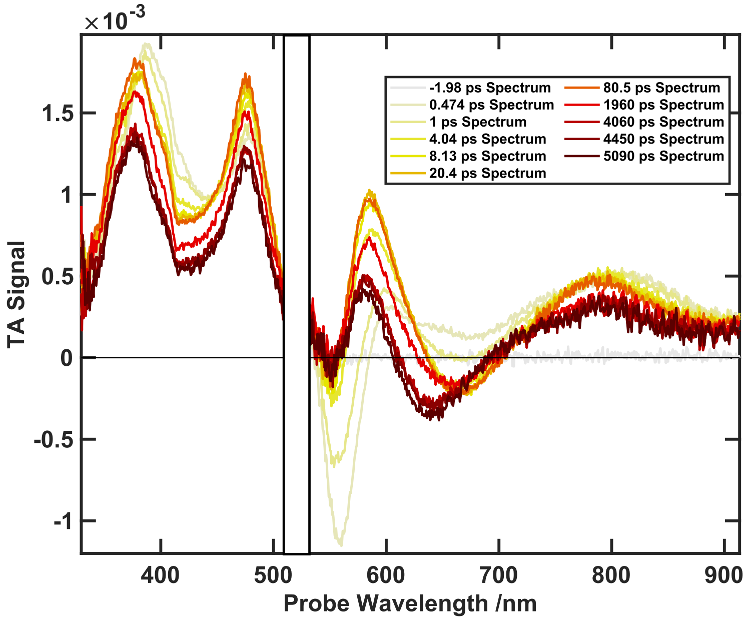

Figure S46. Spectral slices from a femtosecond transient absorption experiment performed on 1.0 mM **Tc-DE** in DMF after excitation at 520 nm with 55 μW.

__**Tc-DE**: 0.1 mM in toluene__

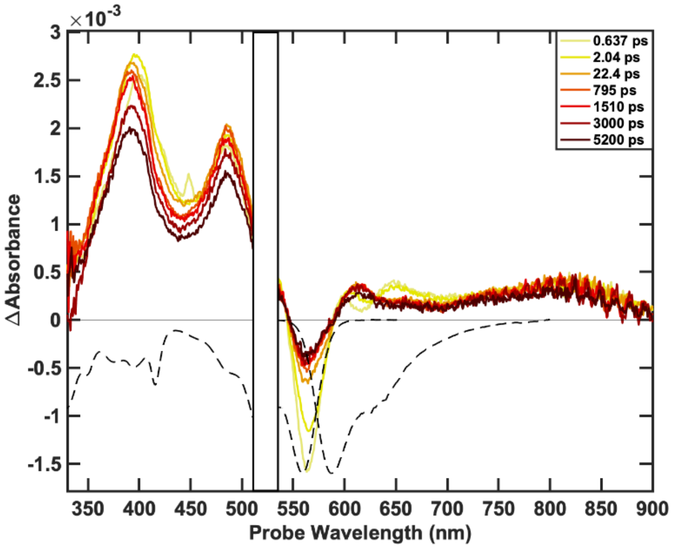

Figure S47. Spectral slices from a femtosecond transient absorption experiment performed on approximately 0.1 mM **Tc-DE** in toluene after excitation at 520 nm with 55 μW.

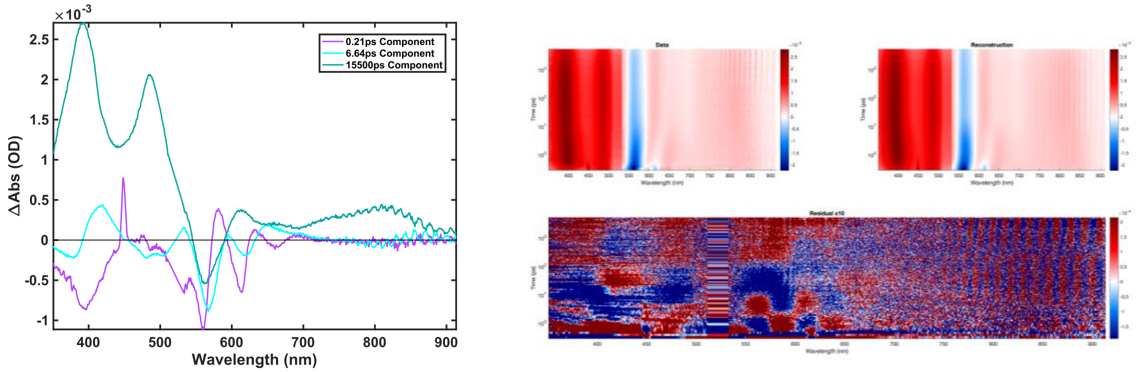

Figure S48. **Left**) Three-state decay associated spectra retrieved from global analysis of the femtosecond transient absorption of 0.1 mM **Tc-DE** in toluene. A sequential model of A → B → C → 0 was applied. **Right**) The raw fsTA data (upper left) collected for 0.1 mM **Tc-DE** in toluene along with a reconstructed model (upper right) composed of an A → B → C → 0 process.

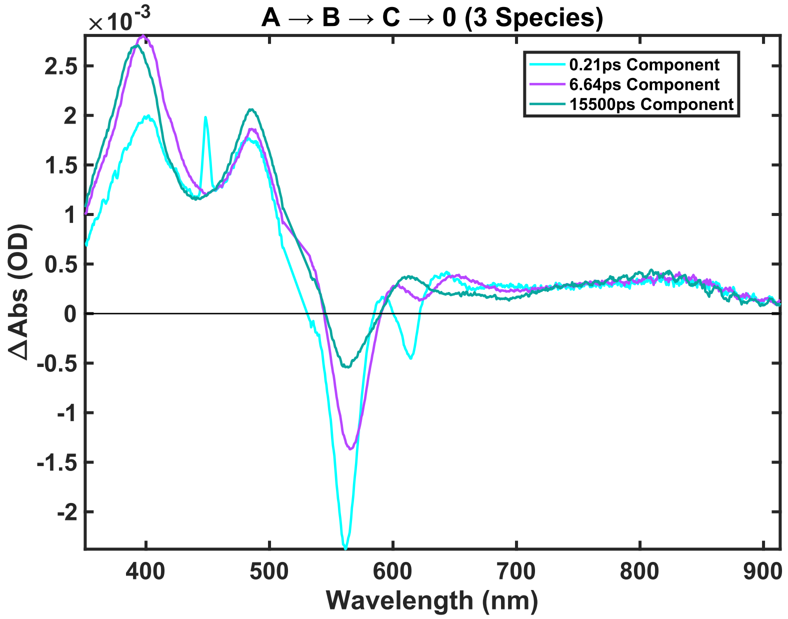

Figure S49. Species associated spectra extracted from global analysis of the femtosecond transient experiment absorption performed on 0.1 mM **Tc-DE** in toluene shown in **Figure S47** and application of linear three component model A $\rightarrow$ B $\rightarrow$ C $\rightarrow$ 0.

<u>**Tc-DE**: 0.1 mM in acetone</u>

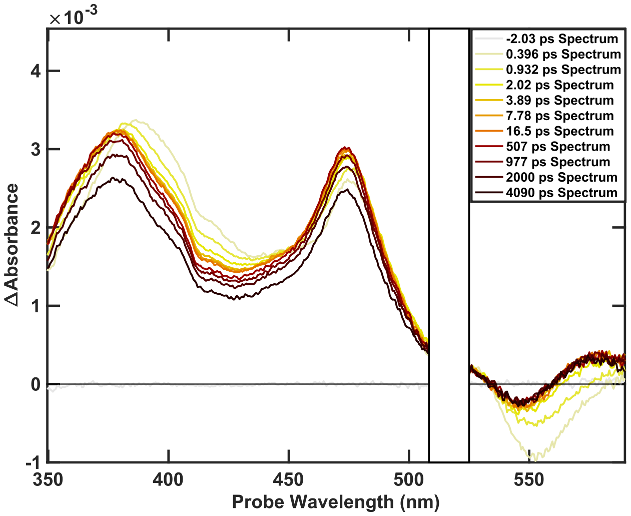

Figure S50. Spectral slices from a femtosecond transient absorption experiment performed on approximately 0.1 mM **Tc-DE** in acetone after excitation at 520 nm with 55 μW.

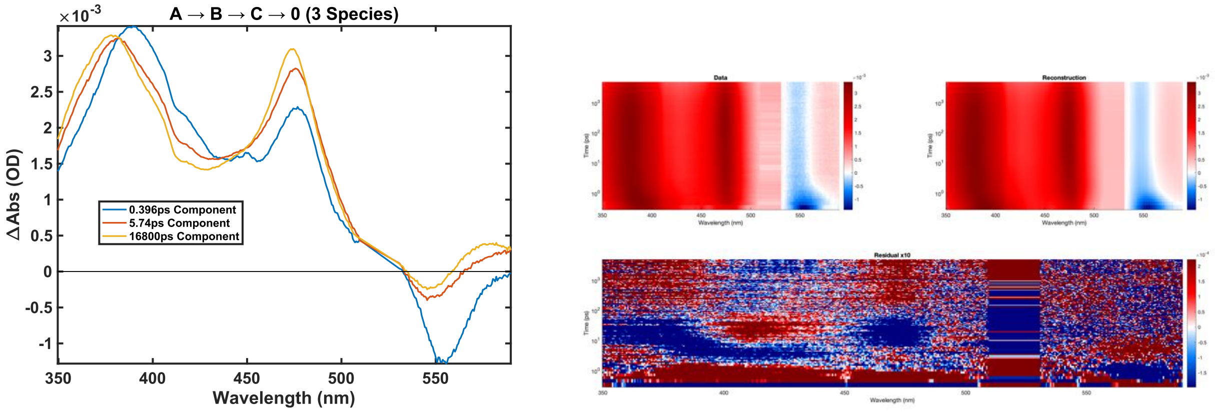

Figure S51. Left) Three-state species associated spectra retrieved from global analysis of the femtosecond transient absorption of 0.1 mM **Tc-DE** in acetone. A sequential model of A → B → C → 0 was applied. **Right**) The raw fsTA data (upper left) collected for 0.11 mM **Tc-DE** in acetone along with a reconstructed model (upper right) composed of an A → B → C → 0 process.

Triplet Sensitization

Triplet Sensitization: **TIPS-Tc** in THF

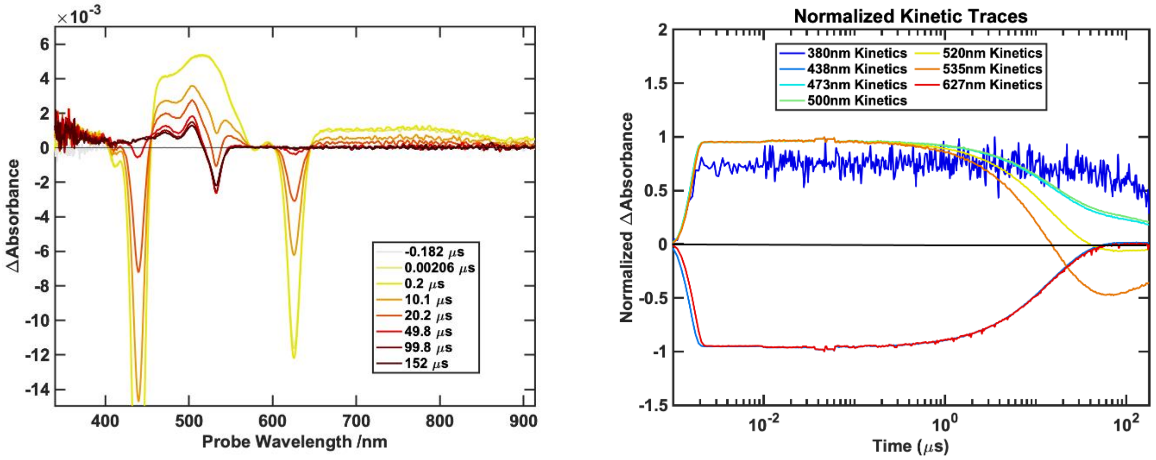

Figure S52. Spectral slices and select normalized kinetics traces from the triplet sensitization of **TIPS-Tc** by **PdPh$_4$TBP** in THF at room temperature after excitation at 625 nm with 36 μW.

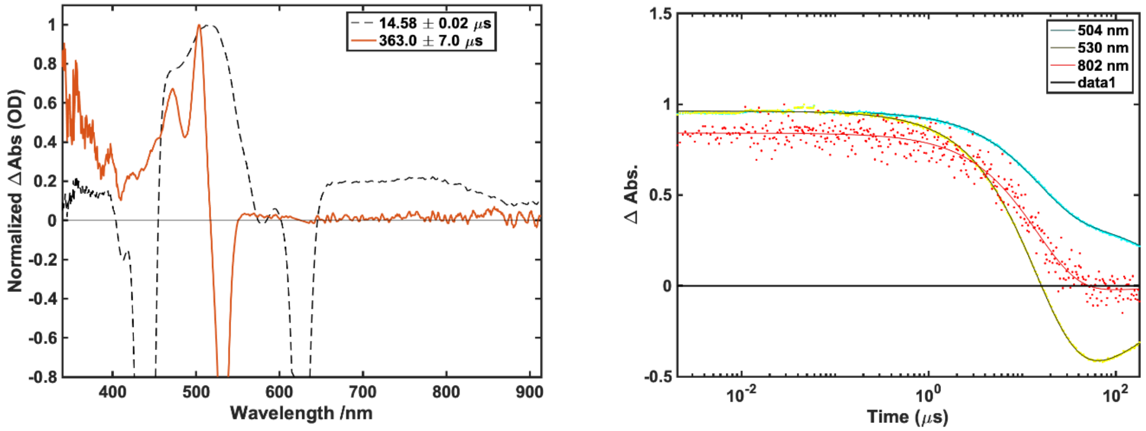

Figure S53. Left) Two-state species associated spectra retrieved from global analysis of the triplet sensitization experiment performed on **TIPS-Tc** in THF shown in **Figure S52**. A sequential model of A → B → 0 was applied. The shorter component (14.6 μs) is assigned to **^{3}PdPh$_4$TBP** and the longer component (363 μs) to **^{3}TIPS-Tc. Right)** Single wavelength kinetics extracted from the data (dots) and the global fit (solid lines).

Triplet Sensitization: **Tc-DE** in THF

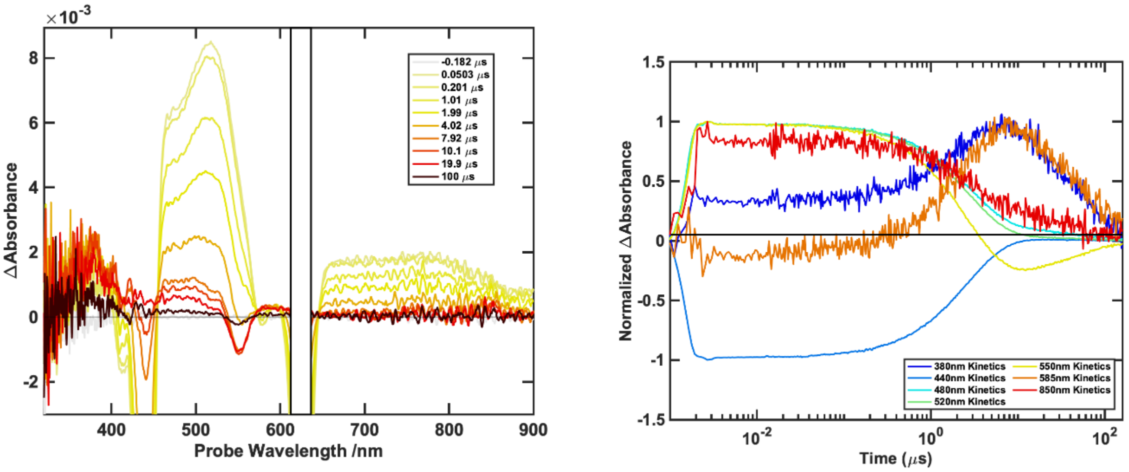

Figure S54. Spectral slices and select normalized kinetics traces from the triplet sensitization of **Tc-DE** by **PdPh$_4$TBP** in THF at room temperature after excitation at 625 nm with 82 μW.

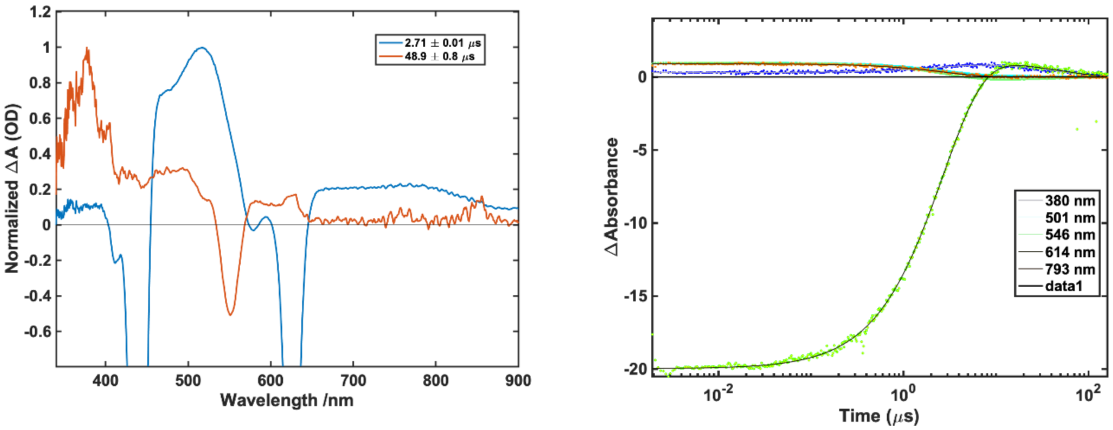

Figure S55. **Left**) Two-state species associated spectra retrieved from global analysis of the triplet sensitization experiment performed on **Tc-DE** in THF shown in **Figure S54**. A sequential model of A → B → 0 was applied. The shorter component (2.71 μs) is assigned to **^{3}PdPh$_4$TBP** and the longer component (48.9 μs) to **^{3}Tc-DE. Right)** Single wavelength kinetics extracted from the data (dots) and the global fit (solid lines).

Triplet Sensitization: 0.1 mM **Tc-DA** in THF

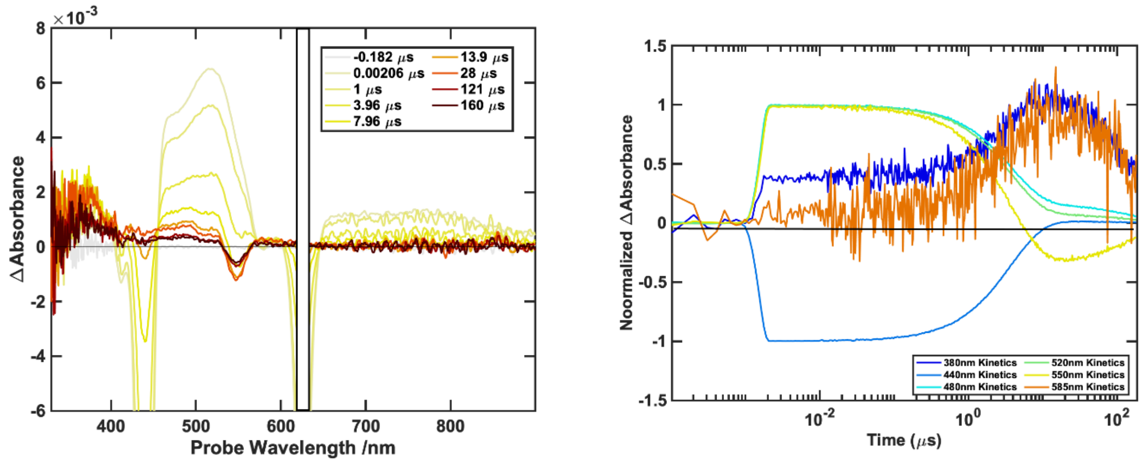

Figure S56. Spectral slices and select normalized kinetics traces from the triplet sensitization of 0.1 mM **Tc-DA** by **PdPh4TBP** in THF at room temperature after excitation at 625 nm with 60 μW.

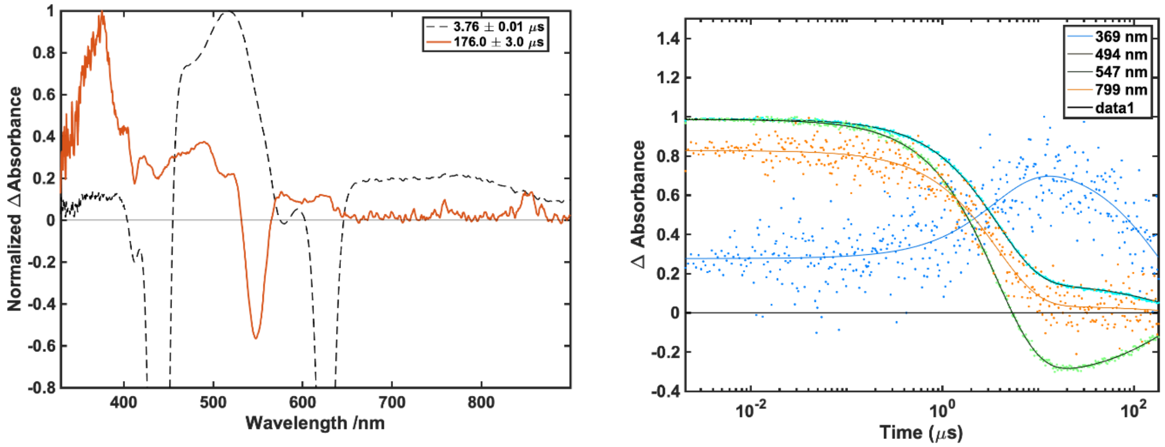

Figure S57. **Left**) Two-state species associated spectra retrieved from global analysis of the triplet sensitization experiment performed on **Tc-DA** in THF shown in **Figure S56**. A sequential model of A → B → 0 was applied. The shorter component (3.76 μs) is assigned to **^{3}PdPh4TBP** and the longer component (176.0 μs) to **^{3}Tc-DA. Right)** Single wavelength kinetics extracted from the data (dots) and the global fit (solid lines).

Triplet Sensitization: 1 mM **Tc-DA** in DMF

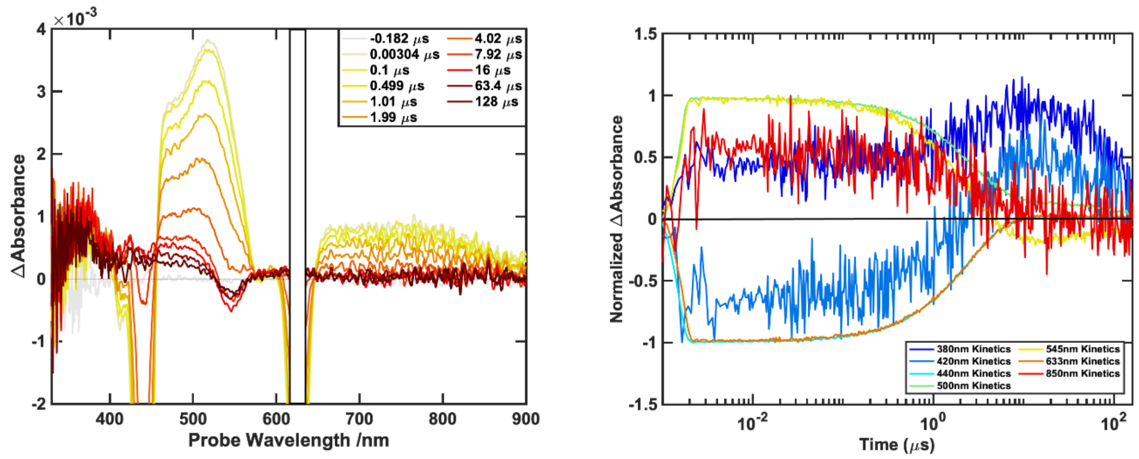

Figure S58. Spectral slices and select normalized kinetics traces from the triplet sensitization of **Tc-DA** (1 mM) by **PdPh$_4$TBP** in DMF at room temperature after excitation at 625 nm with 70 μW.

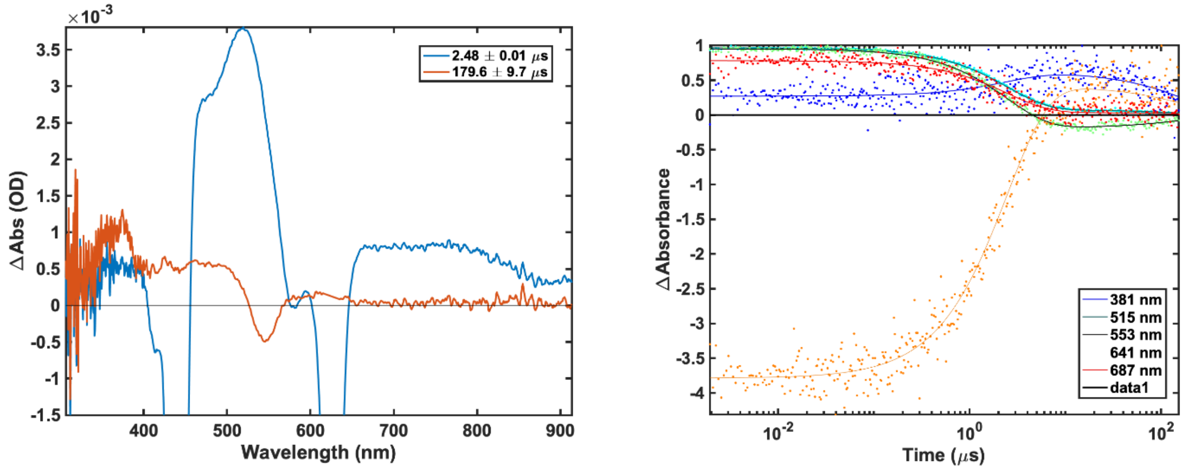

Figure S59. Left) Two-state species associated spectra retrieved from global analysis of the triplet sensitization experiment performed on 1 mM **Tc-DA** in DMF shown in **Figure S58**. A sequential model of A → B → 0 was applied. The shorter component (2.48 μs) is assigned to ^{3}**PdPh$_4$TBP** and the longer component (179.6 μs) to ^{3}**Tc-DA. Right)** Single wavelength kinetics extracted from the data (dots) and the global fit (solid lines).

Triplet Sensitization: 0.020 mM **Tc-DA** in DMF

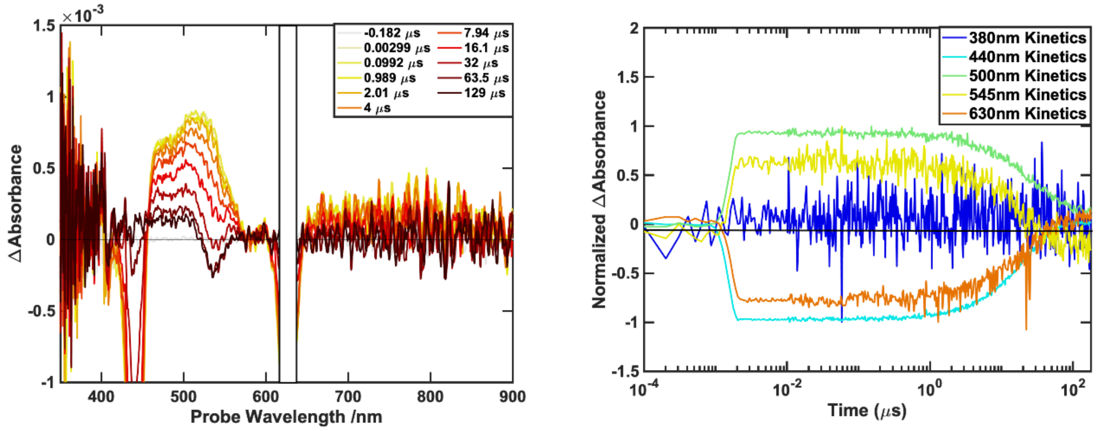

Figure S60. Spectral slices and select normalized kinetics traces from the triplet sensitization of **Tc-DA** (20 μM) by **PdPh₄TBP** in DMF at room temperature after excitation at 625 nm with 36 μW.

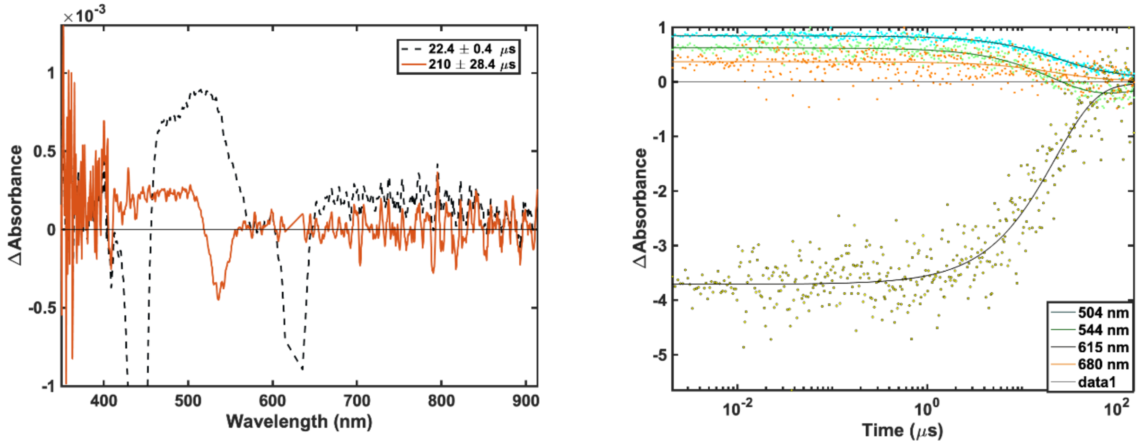

Figure S61. Left) Two-state species associated spectra retrieved from global analysis of the triplet sensitization experiment performed on 20 μM **Tc-DA** in DMF shown in **Figure S60**. A sequential model of A → B → 0 was applied. The shorter component (22.4 μs) is assigned to **^{3}PdPh$_4$TBP** and the longer component (210 μs) to **^{3}Tc-DA. Right)** Single wavelength kinetics extracted from the data (dots) and the global fit (solid lines).

Sandros Plot

A Sandros analysis relates a bimolecular rate constant (triplet energy transfer; k_{TET}) to the driving force of the process (E_D – E_A) (Equation S1).[3] In the limit of a highly exoergic process, the bimolecular rate constant approaches the diffusion limit (k_{dif}). At isoergicity, whether the process occurs in each collision has even odds with it not occurring, such that the bimolecular rate constant approaches half the diffusion limit. If the process is endoergic, then the process becomes increasingly unlikely to occur with larger energy gaps. By varying the donor (thereby altering the energetics of the system), the state energy of the acceptor of interest (E_A) may be determined.

$$k_{TET} = \frac{k_{dif}}{1 + e^{\frac{-(E_D - E_A)}{k_B T}}} \qquad \text{Equation S1}$$

Triplet sensitization of **Tc-DE** by several donor molecules (sensitizers) (**PdOEP** [E_D = 1.87 eV];[4,5] **PdPh$_4$TBP** [E_D = 1.56 eV];[6,7] **ZnPc** [E_D = 1.134 eV];[8] **PdOBuPc** [E_D = 1.13 eV][9]) was performed to generate single-point Stern Volmer quenching constants (Figures S60-63). Via Equation S2, the bimolecular triplet energy transfer rate constant was determined. The quenched triplet lifetime of the sensitizer was assumed to be predominantly due to energy transfer from the sensitizer to the acceptor, without meaningful contribution from the reverse process, given the kinetics fit well to a monoexponential decay model.

$$\frac{\tau_0}{\tau_q} = 1 + k_{TET}\tau_0[TcDE_0] \qquad \text{Equation S2}$$

Quenching Study: [3]**PdOEP** with 93.75 µM **Tc-DE**

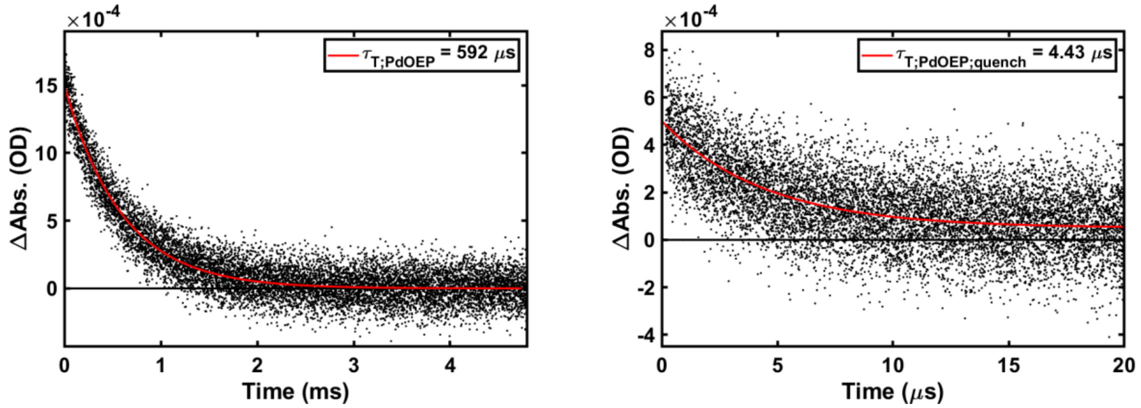

Figure S62. Left) Unquenched [3]**PdOEP** kinetics (dots) collected at 440 nm while exciting with 200 µW of 544 nm laser pulse. Fit (solid line) to a monoexponential decay with no offset. **Right)** Quenched [3]**PdOEP** kinetics (dots) collected at 430 nm while exciting with 400 µW of 544 nm laser pulse. Quencher was 93.75 µM **Tc-DE**. Fit (solid line) to a monoexponential decay with slight offset to account for long-lived ESA of the [3]**Tc-DE**.

Quenching Study: [3]**PdPh$_4$TBP** with 98.87 µM **Tc-DE**

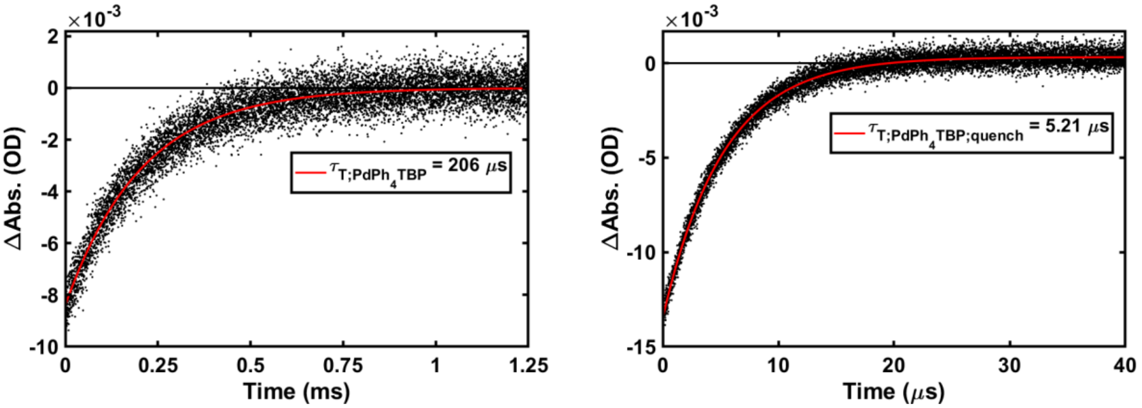

Figure S63. Left) Unquenched [3]**PdPh$_4$TBP** kinetics (dots) collected at 630 nm while exciting with 200 µW of 623 nm laser pulse. Fit (solid line) to a monoexponential decay with no offset. **Right)** Quenched [3]**PdPh$_4$TBP** kinetics (dots) collected at 630 nm while exciting with 200 µW of 623 nm laser pulse. Quencher was 98.87 µM **Tc-DE**. Fit (solid line) to a monoexponential decay with slight offset to account for long-lived ESA of the [3]**Tc-DE**.

Quenching Study: **^{3}ZnPc** with 93.75 µM **Tc-DE**

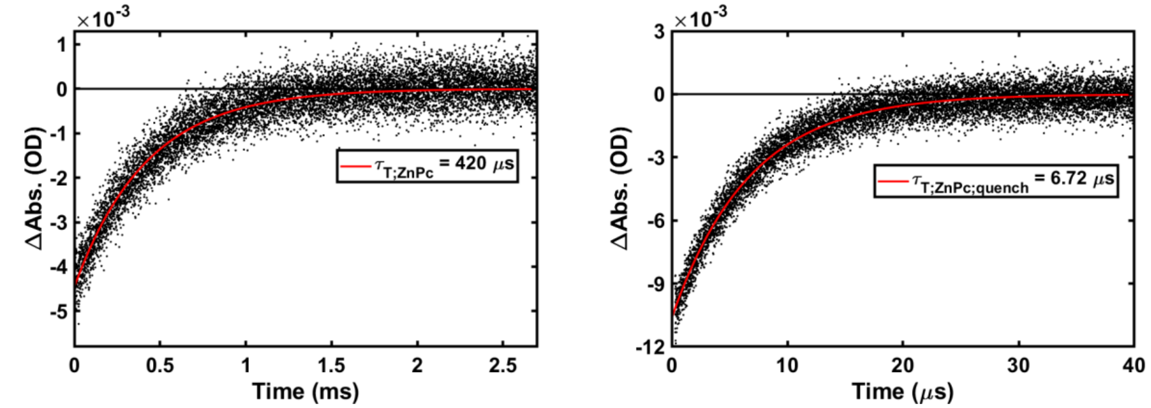

Figure S64. Left) Unquenched **^{3}ZnPc** kinetics (dots) collected at 665 nm while exciting with 310 µW of 640 nm laser pulse. Fit (solid line) to a monoexponential decay with no offset. **Right)** Quenched **^{3}ZnPc** kinetics (dots) collected at 665 nm while exciting with 310 µW of 640 nm laser pulse. Quencher was 93.75 µM **Tc-DE**. Fit (solid line) to a monoexponential decay with no offset.

Quenching Study: **^{3}PdOBuPc** with 100.57 µM **Tc-DE**

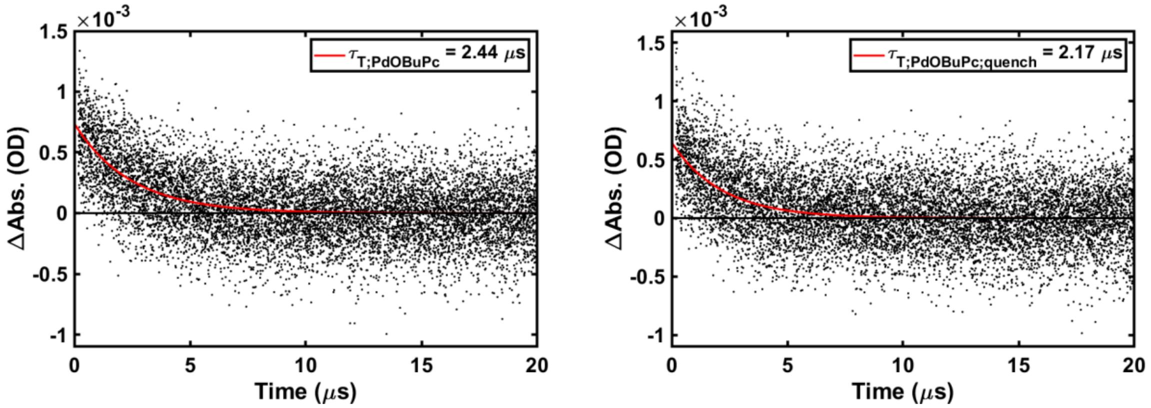

Figure S65. Left) Unquenched **^{3}PdOBuPc** kinetics (dots) collected at 600 nm while exciting with 600 µW of 650 nm laser pulse. Fit (solid line) to a monoexponential decay with no offset. **Right)** Quenched **^{3}PdOBuPc** kinetics (dots) collected at 600 nm while exciting with 600 µW of 650 nm laser pulse. Quencher was 100.57 µM **Tc-DE**. Fit (solid line) to a monoexponential decay with no offset.

In a fifth triplet sensitization utilizing **ZnOBuPc** [E_D = 1.036 eV] (**Figure S64**), the obtained trace required a biexponential fit.[10] Since the long-time component did not fit well to the expected lifetime of **^{3}Tc-DE** (as would be expected given only exoergic energy transfer to **Tc-DE**) further modeling to incorporate significant reverse triplet energy transfer was required. The rate expression is described by Equation S3, and has the analytical solution given by Equation S4.[3]

$$\frac{d[^3S]}{dt} = -k_{T;S}[^3S] - k_{TET}[TcDE_0][^3S] + k_{rTET}[^3TcDE][S_0] \qquad \text{Equation S3}$$

$$[^3S] = C_1e^{-(a-b)t} + C_2e^{-(a+b)t} \qquad \text{Equation S4}$$

$$a = \frac{1}{2}(k_{T;S} + k_{TET}[TcDE_0] + k_{rTET}[S_0] + k_{T;TcDE} \qquad \text{Equation S4a}$$

$$b = \frac{1}{2}[\left(k_{T;S} + k_{TET}[TcDE_0] + k_{rTET}[S_0] + k_{T;TcDE}\right)^2 - 4\left(k_{T;S}k_{rTET}[S_0] + k_{T;S}k_{T;TcDE} + k_{T;TcDE}k_{TET}[TcDE_0]\right)]^{\frac{1}{2}} \qquad \text{Equation S4b}$$

To model the trace, experimental values were substituted into Equation S4 where possible. The initial concentration of **Tc-DE** was calculated utilizing the molar extinction coefficients obtained previously (**Figure S6**). The initial concentration of **ZnOBuPc** was calculated by dividing the peak absorbance of its Q-band (734 nm) by a literature value of the peak molar extinction coefficient (190,000 $M^{-1}cm^{-1}$; though the extinction coefficient was obtained in benzene, order of magnitude is assumed to be consistent in the THF solvent used here).[10] The unimolecular decay rate constants k_T were obtained experimentally (**Figure S53** & **Figure S64**). The reverse triplet energy transfer rate constant k_{rTET} was approximated to be diffusion limited. By baseline

subtracting and normalizing the obtained kinetic trace, the 2nd pre-exponential factor C_2 may be described instead by $1 - C_1$. Utilizing these substitutions, the biexponential expression becomes dependent on two unknowns: the pre-exponential factor C_1 and the forward triplet energy transfer rate constant k_{TET}.

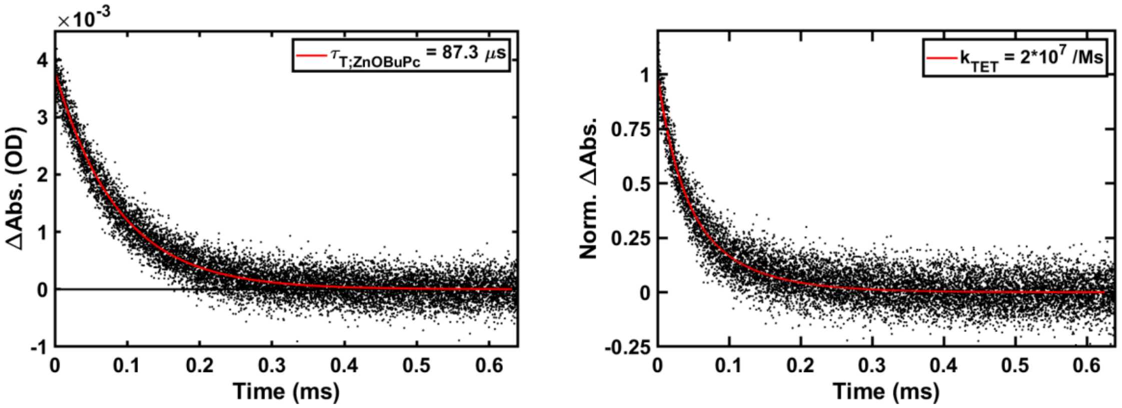

Figure S66. Left) Unquenched **^{3}ZnOBuPc** kinetics (dots) collected at 610 nm while exciting with 160 µW of 645 nm laser pulse. Fit (solid line) to a monoexponential decay with no offset. **Right)** Quenched **^{3}ZnOBuPc** kinetics (dots) collected at 620 nm while exciting with 360 µW of 640 nm laser pulse. Quencher was 98.87 µM **Tc-DE**. Fit (solid line) to a biexponential decay model described in text with no offset. Additional modeling parameters were 5.4 µM ZnOBuPc, $k_{T;ZnOBuPc}$ = 1/(87.3E-6) /s, $k_{T;Tc\text{-}DE}$ = 1/(48.9E-6) /s, k_{rTET} = 2.15E9 /Ms. The "lifetimes" corresponding to the biexponential fit were 30.2 µs and 81.5 µs respectively, with the long lifetime seemingly approaching the natural lifetime of the sensitizer, lending support to the system overall favoring the reverse triplet energy transfer. The retrieved k_{TET} was the value at which the model was unable to improve the fit significantly; fitting differences were negligible below this value, and further had no impact on the outputs from the Sandros analysis (**Figure S65**).

The retrieved k_{TET} of each sensitization were modeled utilizing Equation S1. The energy of **^{3}Tc-DE** (E_A) and the diffusion limited bimolecular rate constant k_{dif} were utilized as fit parameters (**Figure S66**). The diffusion limit was modeled to be 2.15×10^9 /Ms, while E_A was best modeled as 1.14 ± 0.06 eV. There are several ways to improve the application of this analysis. At the cost of more material, varying the concentration of quencher in the independent Stern Volmer analyses could be used to obtain a more robust k_{TET}. Additionally, the triplet energies of the sensitizers used here were pulled from literature, and several were measured under different experimental conditions (solvents and temperature) than subject to here. An inherent assumption in this analysis is that the donor energies are known, and slight variations in these values would lead to varied outputs. Of course, more sensitizers could also be utilized to obtain a better sampling of both exo- and endoergicity. However, this methodology is highly useful for experimentally benchmarking triplet energies that would otherwise be immeasurable in experimental conditions and becomes even more useful if the interest is in comparing different acceptors at constant conditions.

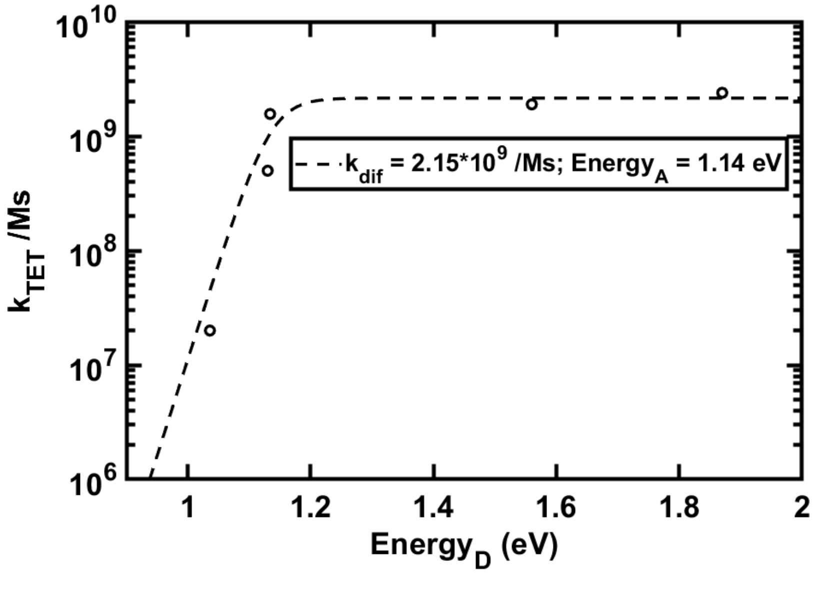

Figure S67. Sandros plot depicting the parameters necessary to describe the diffusional and energetic constraints of triplet energy transfer from a sensitizer to **Tc-DE**.

Table S1. Summary of relevant electronic states and their decay lifetimes for **Tc-DA** and **Tc-DE** across different solvents and concentrations.

System	Electronic State	Decay Lifetime
**TIPS-Tc**	S_1	12.5 ns
	Sensitized T_1	363 ± 7 μs
0.010 mM **Tc-DA** in DMF	S_1	14.2 ± 0.1 ns
	T_1	130 ± 8 μs
0.020 mM **Tc-DA** in DMF	Sensitized T_1	210 ± 28 μs
0.25 mM **Tc-DA** in DMF	Excimer	5.4 ± 0.6 ns
	CT/TT	13.6 ± 1.0 ns
1.0 mM **Tc-DA** in DMF	$S_1 \rightarrow$ Excimer	0.20 ± 0.01 ps
	Excimer	6.4 ± 0.5 ns
	$S_1 \rightarrow$ CT/TTT	2.40 ± 0.02 ps
	CT/TT	17.5 ± 1.2 ns
	Sensitized T_1	180 ± 10 μs
0.1 mM **Tc-DA** in THF	Sensitized T_1	176 ± 3 μs
0.11 mM **Tc-DE** in THF	Sensitized T_1	49 ± 1 μs
0.11 mM **Tc-DE** in DMF	$S_1 \rightarrow$ CT/TT	3.2 ± 0.1 ps
	CT/TT	16.5 ± 0.2 ns
0.11 mM **Tc-DE** in Acetone	$S_1 \rightarrow$ CT/TT	5.7 ± 0.2 ps
0.11 mM **Tc-DE** in Toluene	$S_1 \rightarrow$ CT/TT	6.6 ± 0.1 ps

Electrochemical Characterization

Cyclic voltammetry (CV) and spectroelectrochemistry experiments were performed in a glovebox under an inert atmosphere of argon. For cyclic voltammetry, either anhydrous tetrahydrofuran (THF) or anhydrous dimethylformamide (DMF) was used to perform the experiment and is defined for each experiment within the text. Cyclic voltammetry was performed with an electrochemical analyzer (CH Instruments 601C) using a 3-electrode setup consisting of: a freshly polished platinum disk working electrode (3 mm diameter), a 0.5 mm platinum wire counter-electrode, and a freshly prepared 0.01 M $Ag/AgNO_3$ reference electrode dissolved in either anhydrous DMF or anhydrous acetonitrile (for those experiments run in THF) with a 0.1 M tetrabutylammonium hexafluorophosphate ($TBAPF_6$) supporting electrolyte separated from solution with a porous frit. Cyclic voltammograms were scanned in the negative direction for all cyclic voltammetry experiments. After all cyclic voltammetry measurements were performed, ferrocene was spiked into solution to be used as an internal standard. A final cyclic voltammogram was collected containing the ferrocenium/ferrocene couple at the scan rate defined within the text. During post data collection analysis, the ferrocenium/ferrocene half-wave potential was used as the electrochemical reference, which is to say the ferrocenium/ferrocene half-wave was corrected so as to occur at 0 V, and all electrochemical experiments are reported relative to this reference unless specified otherwise. For the scan rate experiments, data were collected from the slowest scan rate to the fastest scan rate. Before starting a new CV experiment, the solution was mixed via pipette to ensure homogeneity and allowed to rest for 1 minute before the next experiment was run.

Spectroelectrochemical measurements were performed in anhydrous THF using an electrochemical analyzer (CH Instruments 601C) in a 3-electrode setup with a constant applied potential at values indicated in the text. Experiments were performed within a 2 mm pathlength cuvette (1 cm wide and ~ 2 cm tall) which was equipped with a platinum mesh (0.6 cm x 2 cm) working electrode, 0.5 mm platinum wire counter electrode, and a 0.01 M Ag/AgNO3 reference electrode within a 0.1 M $TBAPF_6$ MeCN solution separated from solution with a porous frit. Optical data collection was performed using an Avantes Avalight DHc white light source coupled with an Ocean Insight P600-2-VIS-NIR fiber optic cable which was passed into the

glove box through an Avantes vacuum feedthrough (FC-VFT UVIR600) integrated into a custom milled KF flange. White light passed through the sample and coupled to a separate fiber optic cable back out through the glovebox in the same manner where it was collected with an Avantes Starlight Avaspec 3648 spectrometer then processed using Avasoft software. Absorption spectra, after applying a baseline correction and appropriate solvent (including electrolyte) blank to the software, were collected as a function of time at a constant applied potential.

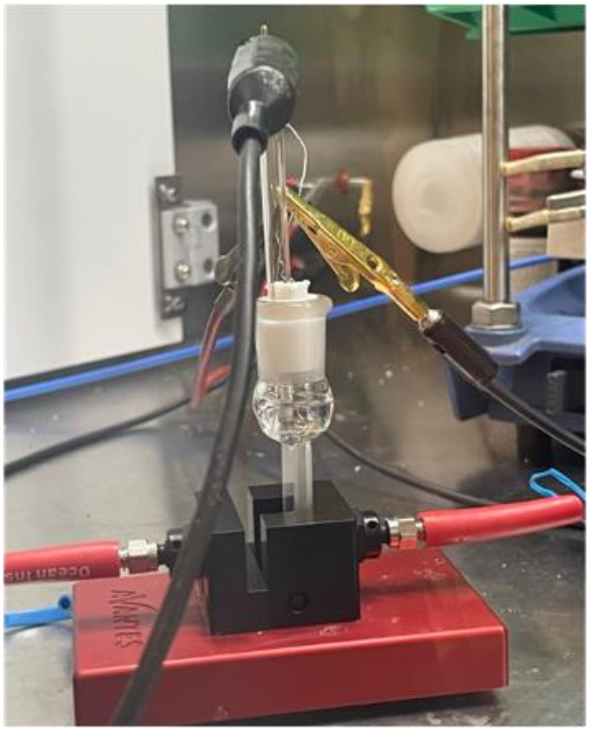

Figure S68. The three electrode spectroelecrochemical setup using a 2 mm cuvette equipped with a platinum mesh (0.6 cm x 2 cm) working electrode, 0.5 mm platinum wire counter electrode, and a 0.01 M Ag/$AgNO_3$ reference electrode in MeCN with 0.1 M $TBAPF_6$ supporting electrolyte separated from experimental solution via a porous frit.

Cyclic Voltammetry

To determine the energies of the HOMO and LUMO levels of **Tc-DE** and accordingly assess the viability of accessing a CT state, cyclic voltammetry was performed in THF (**Figures S71-72**). The first reduction of **Tc-DE** (**Tc-DE/Tc-DE**$^{\cdot-}$) appears reversible and occurs with a half-wave potential of -1.33 V vs Fc^+/Fc. The oxidation of **Tc-DE** (**Tc-DE**$^{\cdot+}$**/Tc-DE**), however, appears as an irreversible feature. A large change in current occurs at +0.8 V vs Fc^+/Fc and continues to increase up to +0.98 V vs Fc^+/Fc, however, when reversing back to more negative potentials an equivalent return current was not observed. A proxy for the energy of an intermolecular CT state consisting of two transiently associated **Tc-DE** molecules (neglecting coulombic stabilization) can be taken as the combined gap (E_{gap}) between the one electron reduction and one electron oxidation events and is computed to be 2.31 V which is approximately 150 meV above the optical gap of the **Tc-DE** S_1. For comparison, **TIPS-Tc** in THF exhibits a reversible redox event with a half-wave potential of -1.69 V vs Fc^+/Fc associated with a one-electron reduction and a quasi-reversible redox oxidation event with a half-wave potential of +0.73 V vs Fc^+/Fc resulting in an E_{gap}= 2.42 V.

Cyclic Voltammetry: 1.0 mM **TIPS-Tc + Ferrocene**

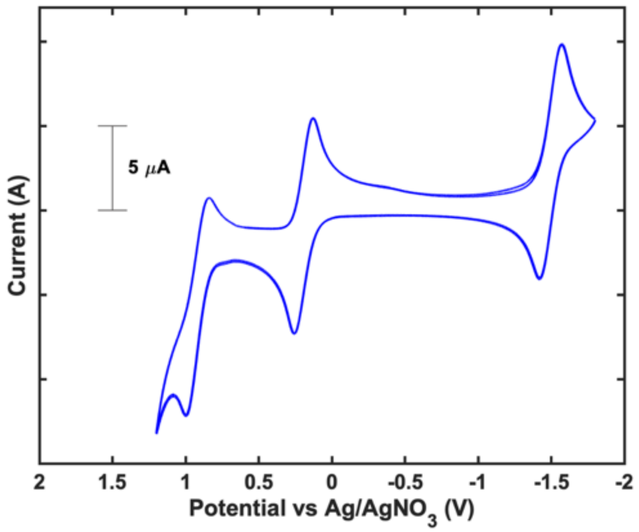

Figure S69. Cyclic voltammogram recorded at 100 mV/s for **TIPS-Tc** (1 mM) in a 0.1 M $TBAPF_6$ THF solution scanning from +1.2 to –1.8 V and back vs $Ag/AgNO_3$. Ferrocene is included within this voltammogram as an internal standard and the Fc^+/Fc appears within the experiment with a half-wave potential of +0.19 V vs. $Ag/AgNO_3$.

Cyclic Voltammetry: 1.0 mM **TIPS-Tc**

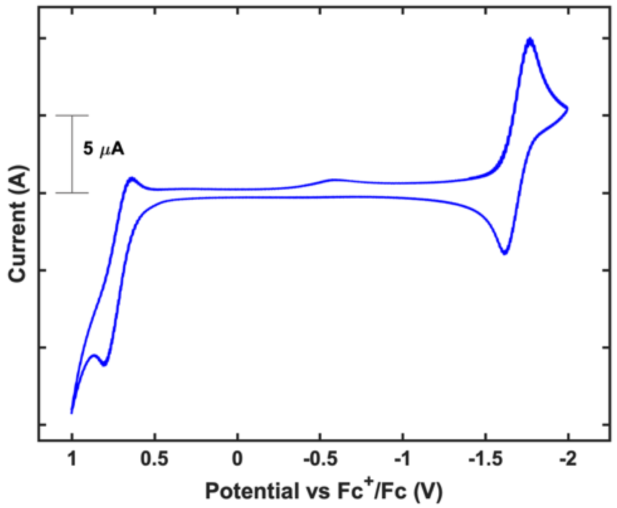

Figure S70. Cyclic voltammogram recorded at 100 mV/s for **TIPS-Tc** (1 mM) in a 0.1 M $TBAPF_6$ THF solution scanning from +1.0 to –2 V and back vs Fc^+/Fc.

Cyclic Voltammetry: 1.0 mM **Tc-DE**

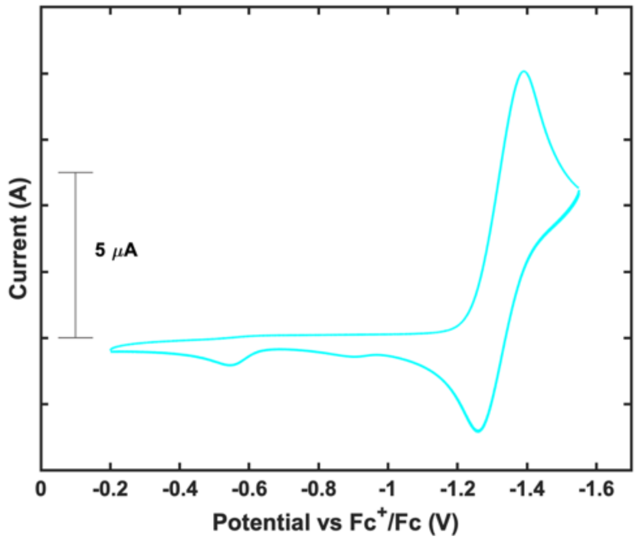

Figure S71. Cyclic voltammogram recorded at 100 mV/s for **Tc-DE** (1 mM) in a 0.1 M $TBAPF_6$ THF solution scanning from -0.2 to –1.55 V and back vs Fc^+/Fc.

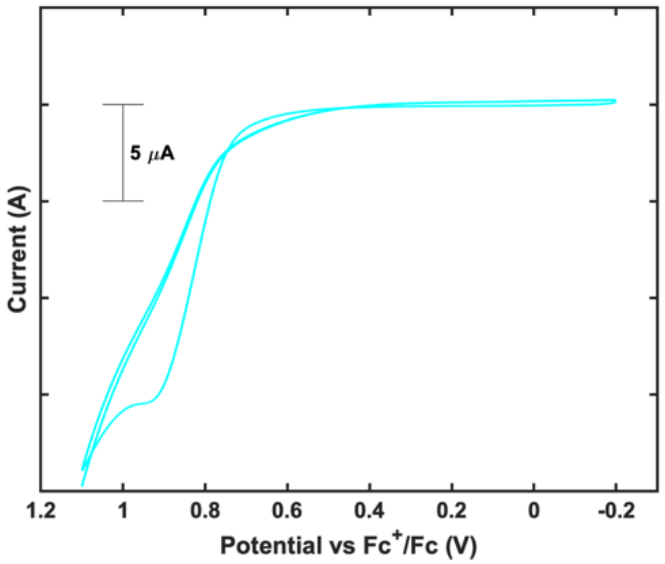

Figure S72. Cyclic voltammogram recorded at 100 mV/s for **Tc-DE** (1 mM) in a 0.1 M $TBAPF_6$ THF solution scanning from +1.1 to –0.2 V and back vs Fc^+/Fc.

Spectroelectrochemistry

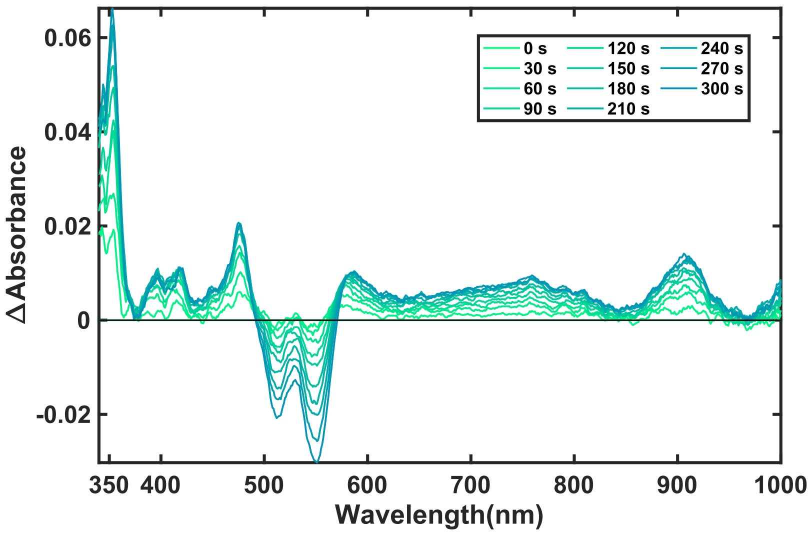

Figure S73. Spectroelectrochemical study of **Tc-DE** in 0.1 M $TBAPF_6$ THF solution with the Pt mesh working electrode held just past the $1e^-$ reducing potential at –1.4 V vs Fc^+/Fc.

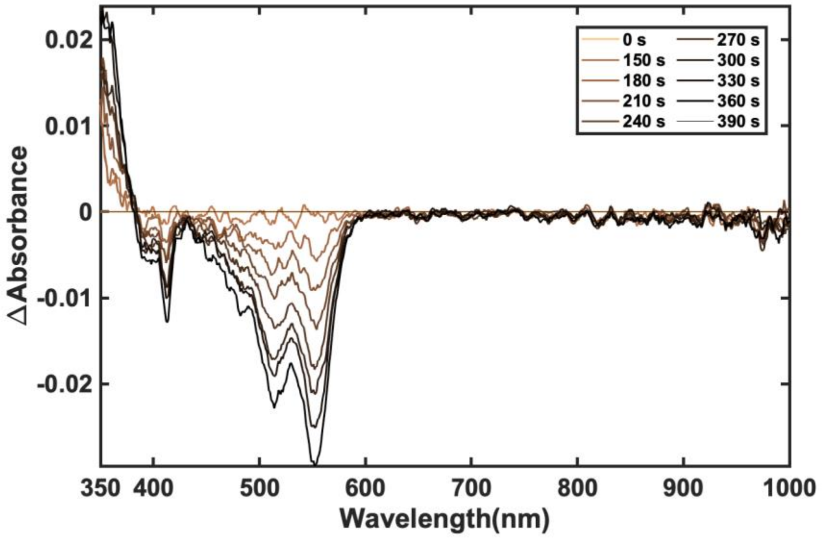

Figure S74. Spectroelectrochemical study of **Tc-DE** in 0.1 M $TBAPF_6$ THF solution with the Pt mesh working electrode held just past the $1e^-$ oxidation potential at +1.2 V vs Fc^+/Fc.

Chemical Oxidation of Tc-DE with $NOBF_4$

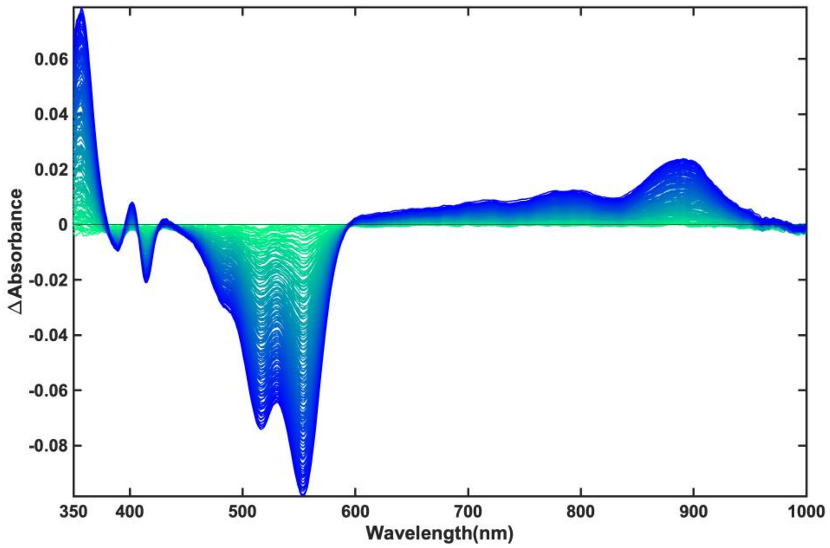

Figure S75. Delta absorption spectrum showing the change in absorbance across 25 minutes after addition of NOBF4 to a solution of **Tc-DE** in anhydrous DCM under an inert atmosphere. The resulting spectrum characterizes the **Tc-DE**$^{\cdot+}$ which is produced through chemical oxidation of **Tc-DE** by $NOBF_4$. This experiment highlights that the delta absorbance spectrum produced by electrochemically oxidizing **Tc-DE** is likely a degradation product.

Synthesis

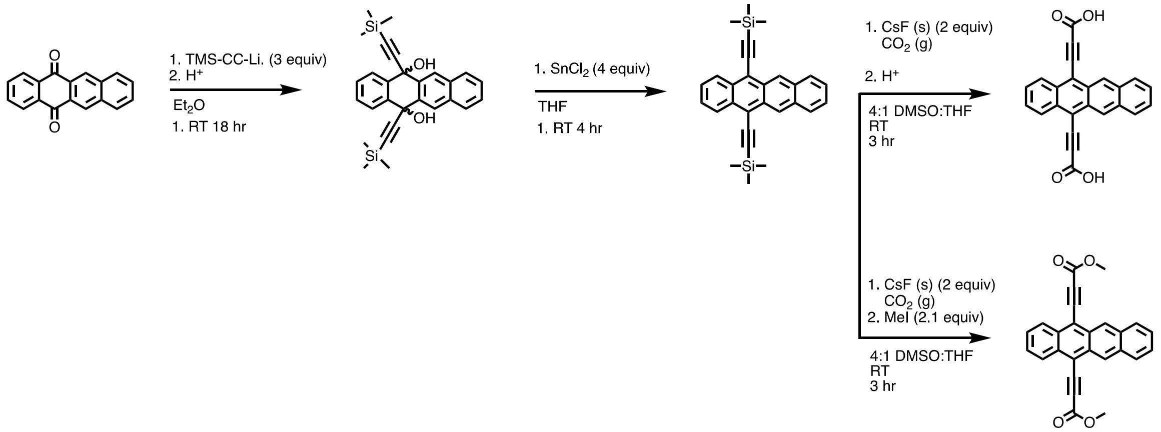

Scheme S1. Synthesis of **Tc-DA** and **Tc-DE** starting from 5,12-napthacenequinone.

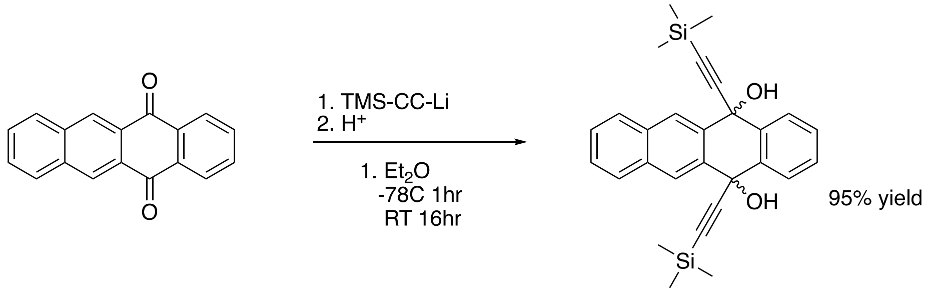

Synthesis of 5,12-Bis[(trimethylsilyl)ethynyl]-tetracene-5,12-diol

To a 100 mL round bottom was added 5,12-napthacenequinone (1g, 3.84 mmol) and placed under high vacuum for 1 hour to make the reagent as moisture free as possible. While this is under vacuum, a separate 50 mL pear shaped flask equipped with a Teflon magnetic stir bar was capped with a septum and then cycled through N_2/high vacuum three times. Approximately 25 mL of freshly distilled diethyl ether was cannulated into the pear shaped flask. This was followed by the addition of trimethylsilyl acetylene (1.132 g, 11.5*10^-2 mmol) via a vacuum dried 1 mL gas-tight glass syringe. The pear flask was then placed in a dry ice/IPA bath and allowed to cool to -78 C. Once cooled, n-butyl lithium (4.38 mL from a 2.5 M solution in hexanes, 11.5 mmol) was added via a vacuum dried 10mL gas-tight glass syringe dropwise. The reaction was allowed to stir at -78 C for 10 minutes before being moved to brine-ice bath to stir at -10 C for an additional 40 minutes. The n-butyl lithium trimethyl silyl acetylene reaction was then cannulated over the 5,12-napthacenequinone under nitrogen dropwise. The reaction is left to stir in the 100 mL round bottom overnight at room temperature covered in foil.

The next day the solution is clear and ruby red in color (sometimes the reaction maintains the forest green color overnight). The reaction was quenched with approximately 25 mL of a saturated ammonium chloride solution. The organics were extracted with DCM, washed with 30 mL of water two times, then 30 mL of a brine solution, and finally dried with $MgSO_4$. The solvent was then removed in vacuo to produce a relatively pure mixture of the cis and trans isomers as green oil. These isomers can then be isolated via 1:1 DCM:Hexanes to produce an off white solid. 1.63 grams (95%) ^{1}HNMR (300 MHz, ppm, $CDCl_3$): 0.19 (m, 18 H), 3.44 (s, 2 H), 7.47-7.51 (m, 2 H), 7.53-7.56(m, 2 H), 7.91-7.95 (m, 2), 8.11-8.14 (m, 2 H), 8.56 (s, 1 H).

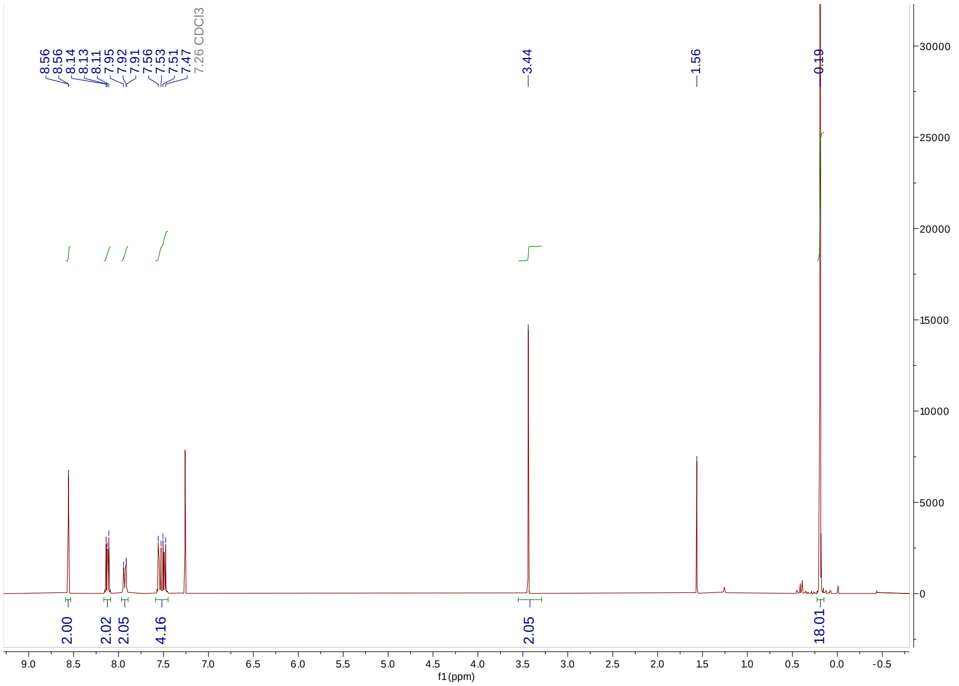

Figure S76. ^{1}H-NMR (300 MHz, $CDCl_3$) of trans-5,12-Bis[(trimethylsilyl)ethynyl]-tetracene-5,12-diol.

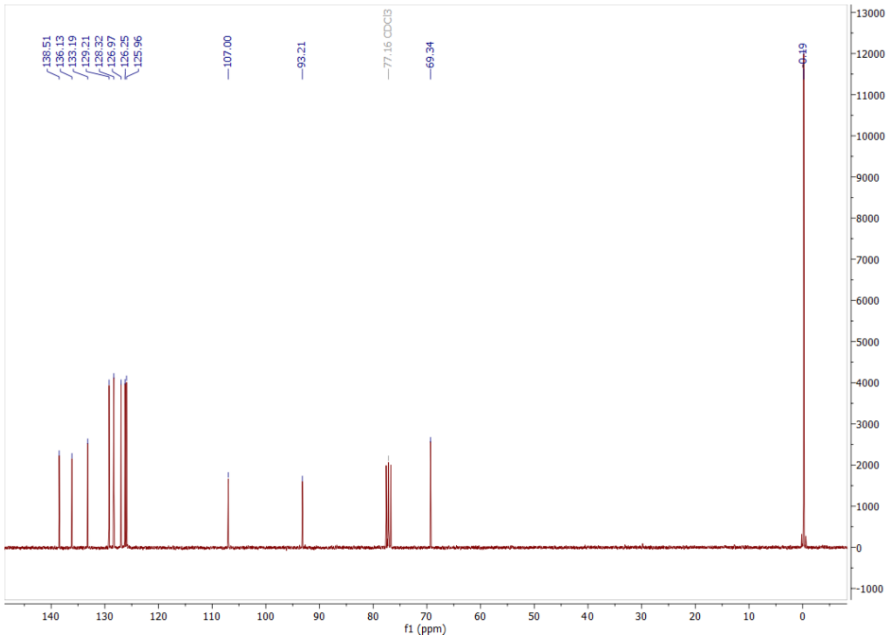

Figure S77. ^{13}C-NMR (75 MHz, $CDCl_3$) of trans-5,12-Bis[(trimethylsilyl)ethynyl]-tetracene-5,12-diol.

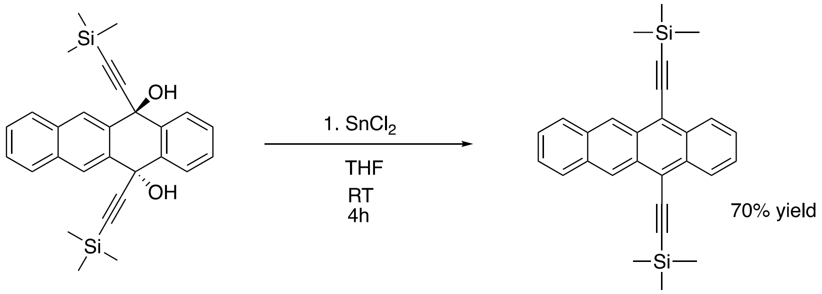

Synthesis of 5,12-Bis[(trimethylsilyl)ethynyl]tetracene

5,12-Bis[(trimethylsilyl)ethynyl]-tetracene-5,12-diol (0.718g, 1.58 mmol) was transferred to an oven dried 25mL round bottom flask equipped with a medium stir bar and dissolved in approximately 15 mL of freshly distilled THF. The solution was then sparged with N_2 for five minutes before adding tin(II) chloride (1.20g, 6.32 mmol) as a solid in one portion. This changes the color of the solution from a clear tan to red almost instantaneously. The reaction flask is covered in foil and allowed to stir in the dark at room temperature for 4 hours. After 4 hours the THF is removed in vacuo. The red solid is then redissolved in chloroform and pushed through a small silica plug with chloroform to remove residual tin(II) chloride. The chloroform is then removed in vacuo. This red solid can then be purified via a silica column with n-hexanes mobile phase to produce TMS-tetracene as a ruby red colored solid. This material is recrystallized from chloroform layered with methanol (1:4) to yield red, needle like crystals. 464 mg. (70% yield). ^{1}HNMR (300 MHz, ppm, $CDCl_3$) 0.48 (m, 18 H), 7.46-7.49(m, 2 H), 7.53-7.56 (m, 2H), 8.06-8.10 (m, 2), 8.55-8.59 (m, 2 H), 9.20 (s, 1 H).

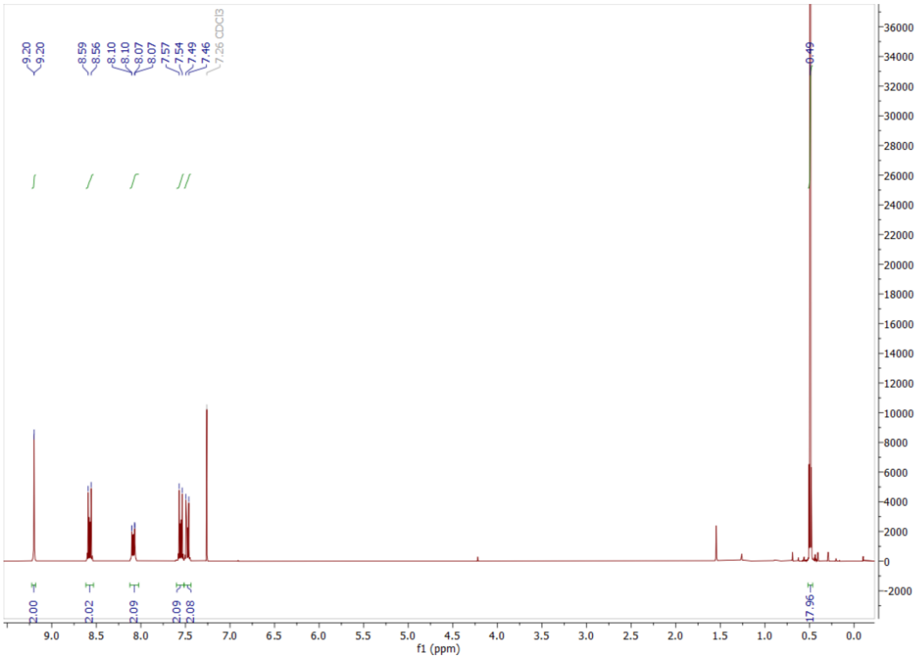

Figure S78. ^{1}H-NMR (300 MHz, $CDCl_3$) of **TMS-Tc**.

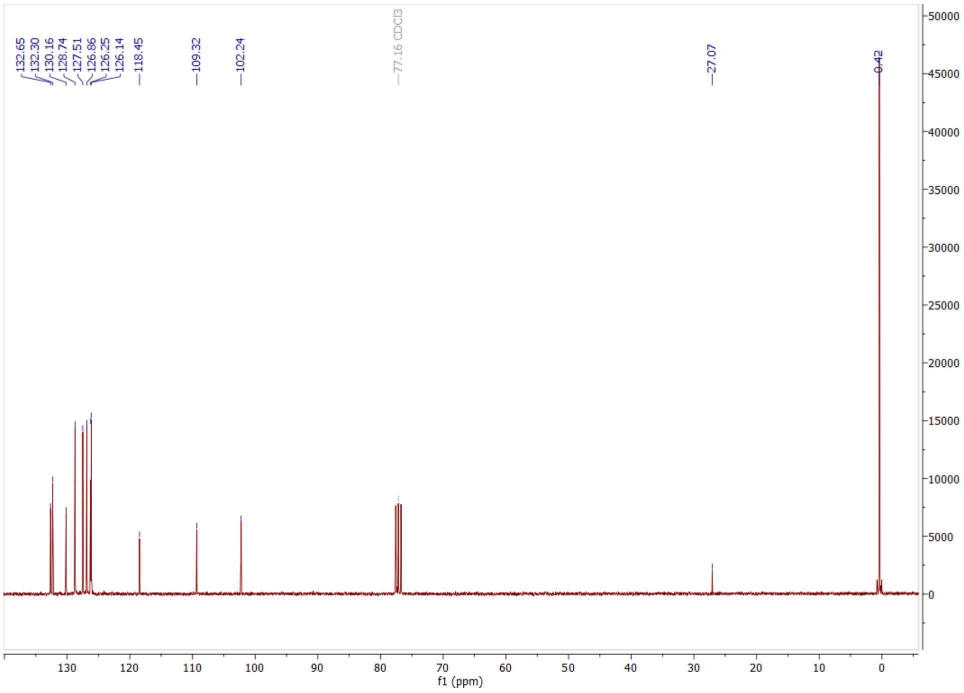

Figure S79. ^{13}C-NMR (75 MHz, $CDCl_3$) of **TMS-Tc**.

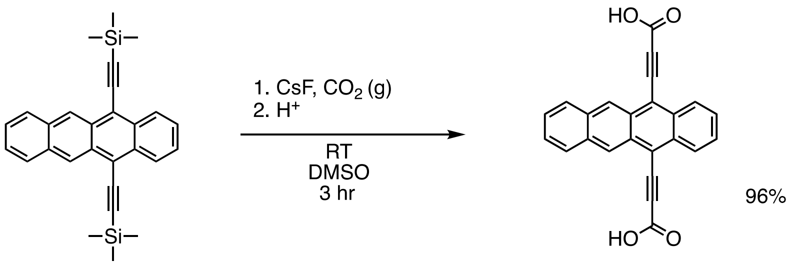

<u>Synthesis of 5,12-Bis[(carboxylic acid)ethynyl]tetracene (**Tc-DA**)</u>

To an oven dried 10 mL round bottom flask equipped with small stir bar was added cesium fluoride (0.087 g, 0.570 mmol, 2 equiv) within a glovebox under an N_2 atmosphere. The round bottom was capped with a septum and pumped out of the glovebox and immediately placed under high vacuum. It was then cycled through N_2/High vacuum three times before 2.5 mL of anhydrous DMSO was added via 2.5 mL vacuum dried glass syringe. The opaque solution was then sparged with CO_2 for 30 minutes before uncapping and adding TMS-tetracene (0.120g, 0.285 mmol, 1 equiv) quickly as a solid in one portion. The round bottom was quickly recapped, and the solution continued sparging with CO_2. The solution quickly took on a wine-red solution. After 4 hours, approximately 5 mL of a 5% KOH(aq) solution was added dropwise to the reaction which turns the solution dark red in color. The crude solution was then poured into a separatory funnel containing 10mL of 5% KOH solution and 10 mL of ethyl acetate. The biphasic solution is swirled, and the aqueous layer is collected and the organics removed. The aqueous layer is washed with ethyl acetate until the organic layers are no longer colored. The collective aqueous layers are acidified with a 1 M HCl (aq) solution dropwise until a blue precipitate forms (pH ~ 2). Diethyl ether is then added to the separatory funnel to dissolve the blue precipitate forming a bright red organic layer. The organic layer is then collected, washed with brine, and dried with $MgSO_4$. The diethyl ether is removed in vacuo, leaving behind **Tc-DA** as a blue solid (100 mg, 96% yield).

^{1}H-NMR (300 MHz, ppm, d^6-DMSO) 7.61-7.64(m, 2 H), 7.77-7.81 (m, 2H), 8.22-8.25 (m, 2), 8.46-8.49 (m, 2 H), 9.13 (s, 1 H). ESI-TOF: m/z [M+H]+ calcd. for $C_{24}H_{12}O_4$: 363.0657, found 363.0659.

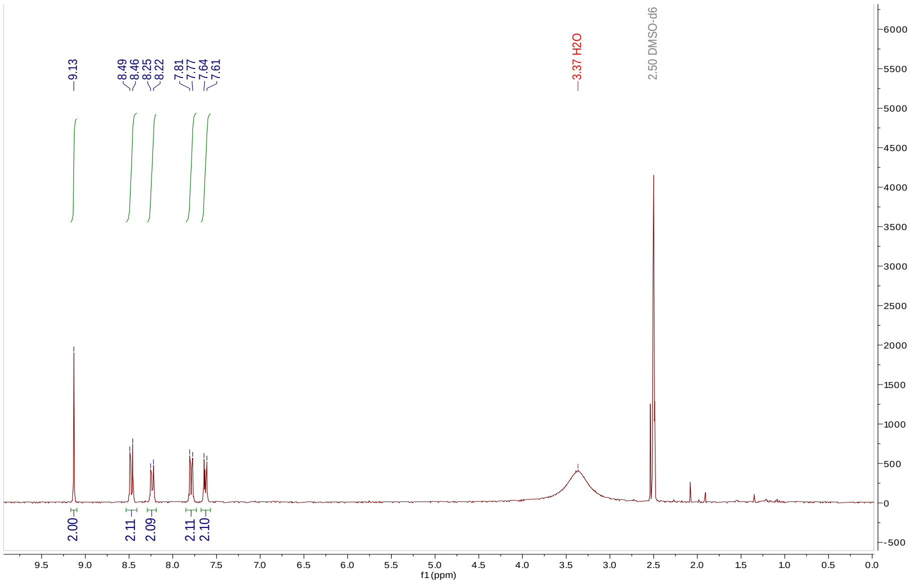

Figure S80. ^{1}H-NMR (300 MHz, d6-DMSO) of **Tc-DA**.

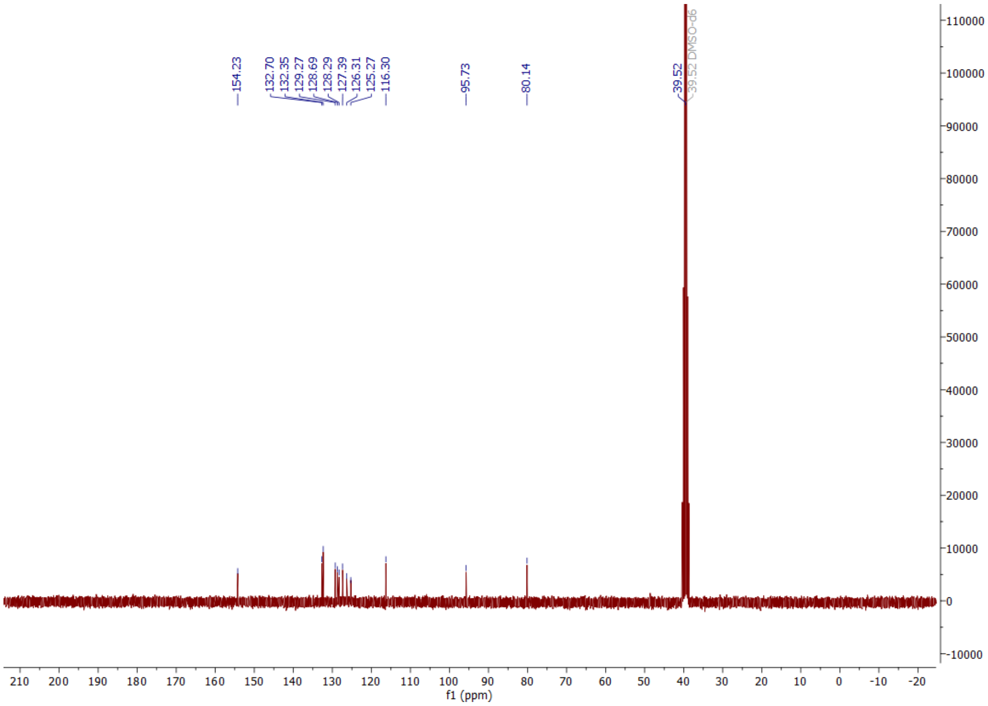

Figure S81. ^{13}C-NMR (75 MHz, d6-DMSO) of **Tc-DA**.

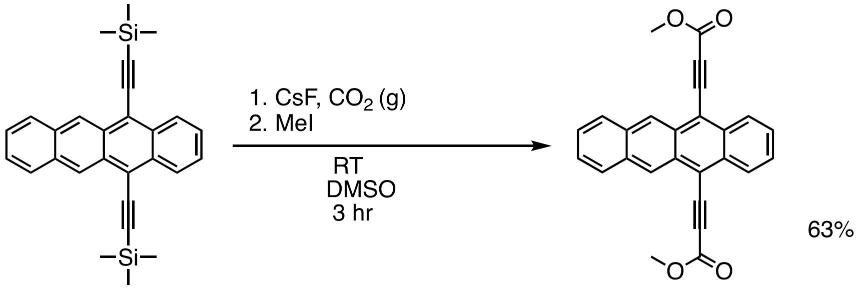

Synthesis of 5,12-methyl-tetracenepropynoate

To an oven dried 5 mL round bottom flask equipped with small stir bar was added cesium fluoride (0.087 g, 0.570 mmol, 2 equiv) within a glovebox under an N_2 atmosphere. The round bottom was capped with a septum and pumped out of the glovebox and immediately placed under high vacuum. It was then cycled through N_2/High vacuum three times before 2.5 mL of anhydrous DMSO was added via 2.5 mL vacuum dried glass syringe. The opaque solution was then sparged with CO_2 for 30 minutes before uncapping and adding TMS-tetracene (0.120 g, 0.285 mmol, 1 equiv) quickly as a solid in one portion. The round bottom was quickly recapped, and the solution was continued to be sparged with CO_2. The solution quickly took on a wine-red solution. After 2 hours, methyl iodide (0.085 g, 0.037 mL, 0.599 mmol, 2.1 equiv) is added dropwise via vacuum dried syringe. CO_2 is shut off and the reaction is kept under positive N_2 pressure. After 1 hour, the reaction is quenched with water and extracted with ethyl acetate. This seems to form an emulsion that is difficult to separate. The aqueous layer is extracted three times with ethyl acetate. The collective organics are washed with brine and dried with magnesium sulfate. Solvent is removed in vacuo to produce a blue solid. The crude solid is purified via silica column chromatography with a 3:1 chloroform:hexanes mobile phase. **Tc-DE** is collected as a purple solid, approximately ~70mg. (63% yield). (Rf = 0.2 in 3:1 Chloroform:Hexanes). ^{1}H-NMR (300 MHz, ppm, CDCl3) 4.02(s, 6 H), 7.47-7.50 (m, 2H), 7.52-7.56 (m, 2H), 8.00-8.04 (m, 2), 8.38-8.41 (m, 2 H), 8.94 (s, 1 H). ESI-TOF: m/z [M+H]+ calcd. for $C_{26}H_{16}O4$: 393.1127, found 393.1121, calcd. for [2*[C26H16O4]+H]+ : 785.2175, found 785.2167.

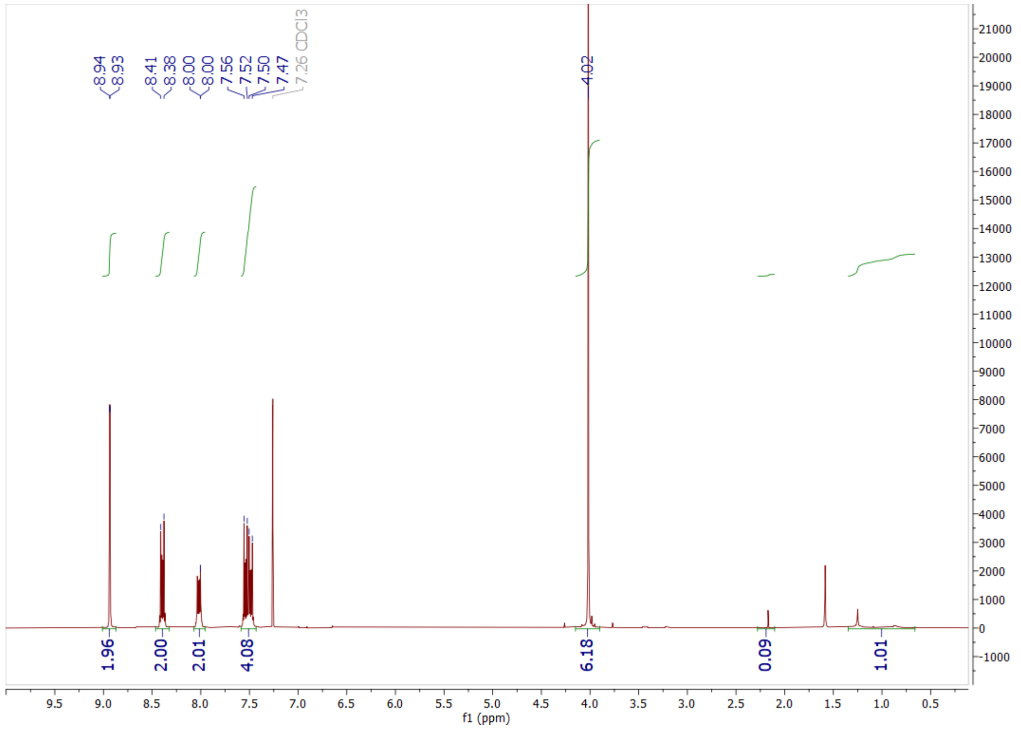

Figure S82. ^{1}H-NMR (300 MHz, $CDCl_3$) of **Tc-DE**.

NMR Experiments

Concentration Dependent ^{1}H-NMR

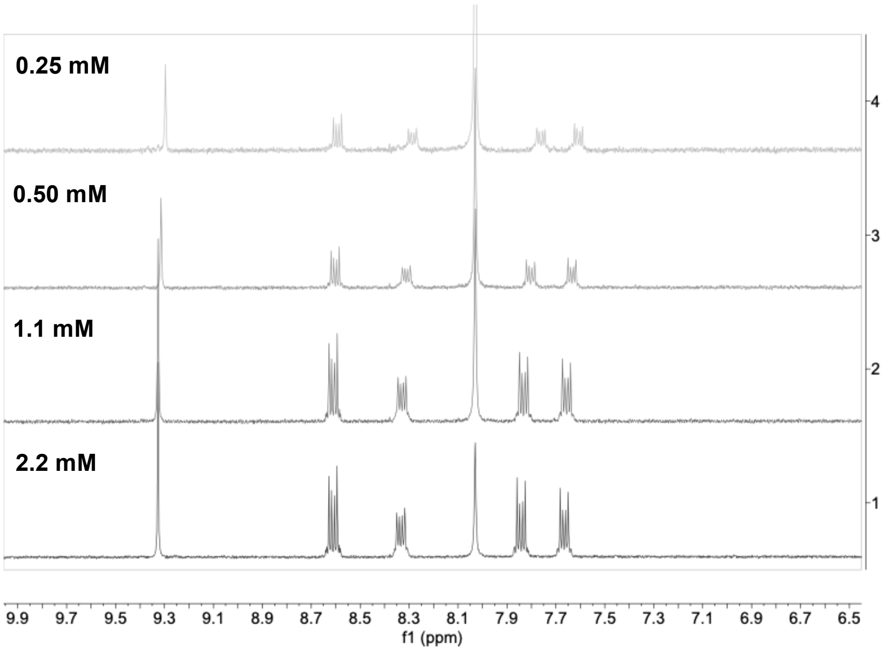

Figure S83. 300 MHz ^{1}H-NMR stacked spectra of **Tc-DA** dissolved in d^7-DMF as a function of concentration. There is a very slight downfield shift of the aromatic proton resonances as concentration is increased.

NOESY

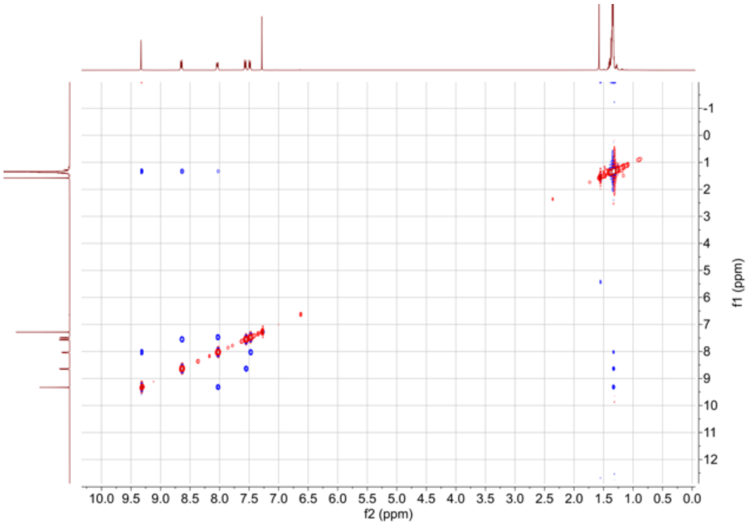

Figure S84. 2D NOESY spectrum of **TIPS-Tc** dissolved in $CDCl_3$.

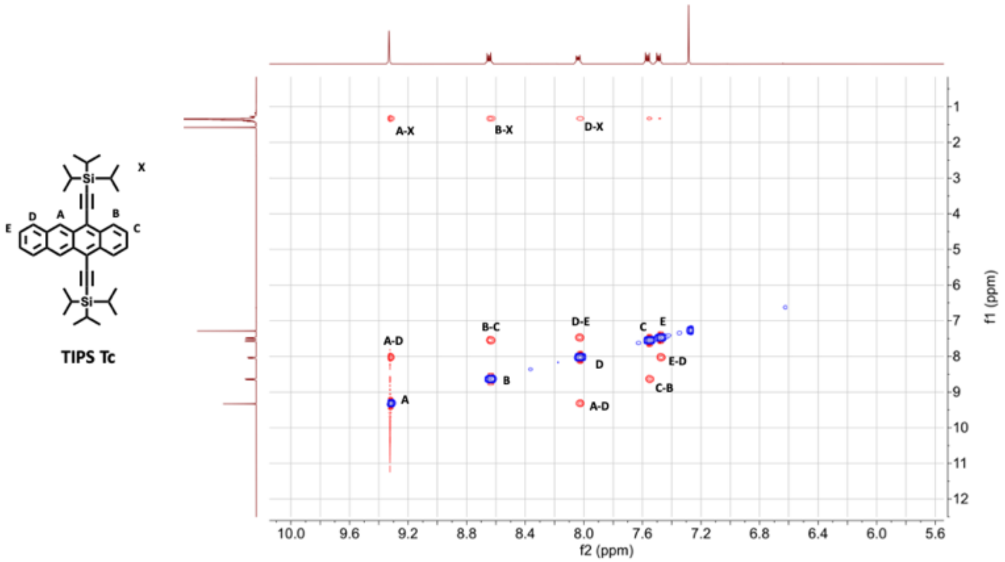

Figure S85. 2D NOESY spectrum of **TIPS-Tc** dissolved in $CDCl_3$ highlighting the aromatic proton resonance region. ^1H resonances are assigned to the TIPS-Tc structure on the left along with their associated NOE couplings observed as cross peaks on the 2D spectrum.

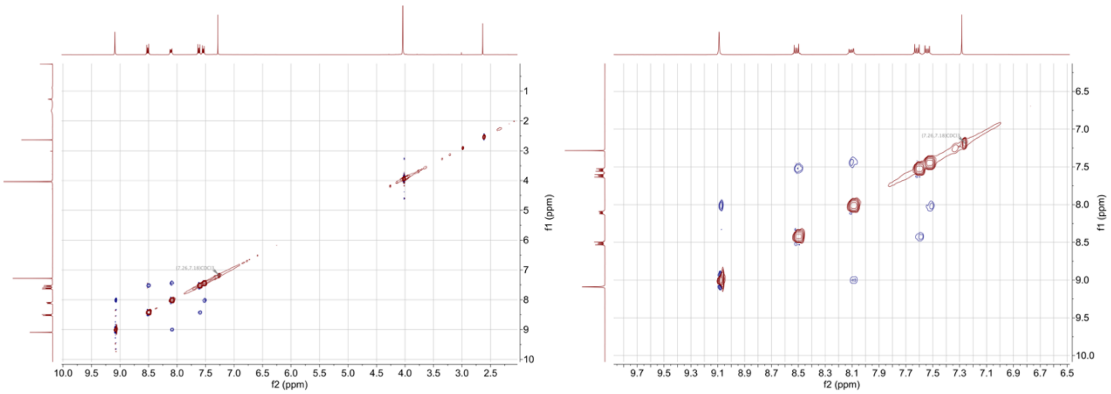

Figure S86. **Left**) Full 2D NOESY spectrum of 20 mM **Tc-DE** dissolved in $CDCl_3$. **Right**) Highlighting the cross peaks found in the aromatic region which resemble those found for **TIPS-Tc** in **Figure S85**.

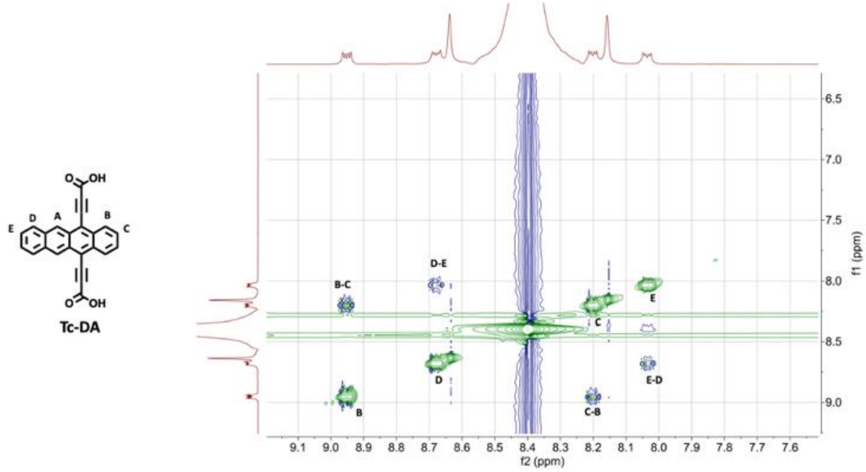

Figure S87. 2D NOESY spectrum of 3 mM **Tc-DA** dissolved in d^7-DMF highlighting the cross peaks found in the aromatic region.

DOSY

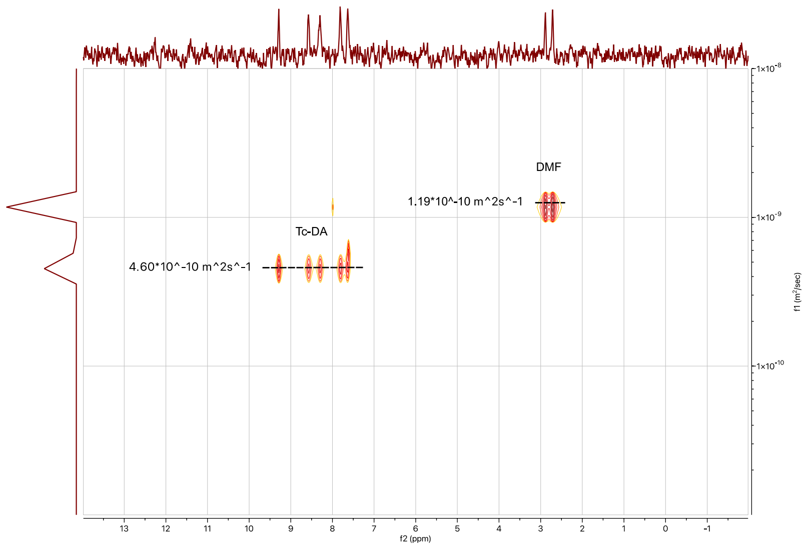

Figure S88. ^{1}H-DOSY NMR spectrum of 3 mM **Tc-DA** dissolved in d^{7}-DMF. Through the Stokes-Einstein law for diffusion, the solvent viscosity of DMF (η = 0.78 mPa*s), temperature of 293.15 K, and diffusion coefficient of 4.60*10^{-10} m^{2}*s^{-1} the hydrodynamic radius was calculated to be 0.58 nm.

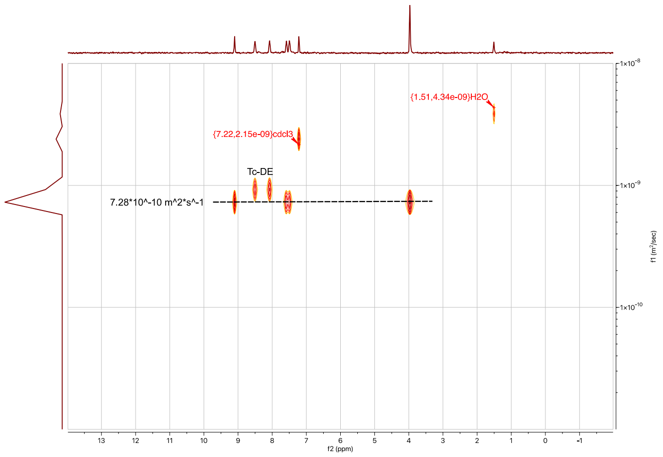

Figure S89. ^{1}H-DOSY NMR spectrum of 6.6 mM **Tc-DE** dissolved in $CDCl_3$. **Tc-DE** is not sufficiently soluble in DMF and so $CDCl_3$ was used as the solvent for the NMR experiment. Through the Stokes-Einstein law for diffusion, the solvent viscosity of chloroform (η = 0.528 mPa*s), temperature of 293.15 K, and diffusion coefficient of 7.28*10^{-10} m^{2}*s^{-1} the hydrodynamic radius was calculated to be 0.52 nm.

Electronic Structure Theory

Ground state geometry optimizations were performed with density functional theory (DFT) using the range-separated and dispersion corrected functional ωB97xD and the triple-zeta def2-TZVP basis set using the Gaussian 16 software package[11]. Frequencies at the ground state geometries were calculated using the same method; no imaginary frequencies were found. Excited state energies and the Forces on nuclei, or gradients, were calculated for the first ten (twenty) excited states at the optimized ground state monomer (dimer) geometries using TD-DFT in Gaussian 16. Triplet energies were calculated using DFT and the same level of theory; they are from the lowest energy triplet state. Interaction energies were calculated from the ground state energy of an optimized dimer and the single point energies of the single monomer units fixed at the dimer geometries. When explicitly noted, the PCM solvation model was applied for the given solvent—DMF or THF; otherwise, the calculation was performed in the gas phase. The double-zeta basis set def2-SVP was used for initial geometry optimizations. Calculations for the Tc-DA monomer and some dimer geometries were also performed using the quadruple-zeta basis set def2-QZVP and showed no appreciable difference. The M062x functional also gave similar results though the ωB97xD was chosen because it better reproduced the scale of the spectra. The initial choices for functionals and basis sets were based on literature precedence for similar systems[12–14].

The vibrationally resolved electronic spectra were calculated using the Vertical Gradient (VG) approximation for the bright excited states (with electronic oscillator strength > 0.01) in the ^1L_a and ^1L_b spectral range using the software package FCClasses3.[15] The nuclear dependence of electronic coupling terms was calculated with the Franck-Condon approximation and intensities are from the time-dependent method which ensures that spectra are fully converged so that intensities of different transitions may be compared. Calculations were performed at room temperature (298.15 K) and the spectra were convolved with Voigt line shapes with 0.024 eV Gaussian and 0.0025 eV Lorentzian broadening. All transitions are shown at their vertical, bottom of the well, energies and are shifted by 21 nm, which is the difference between the vertical transition energy of the calculated $S0_{Tc\text{-}DA} \rightarrow S1_{Tc\text{-}DA}$ transition (516 nm) and the ^1L_a peak

in the 15 μM spectrum (537 nm, Fig. 2A). For each system, a normalization factor is applied to all calculated transitions that take the most intense transition for that system to one.

The fragment electron transition dipole moments and percent interfragment charge transfer were calculated using Multiwfn 3.8.[16] Input geometries were the optimized ground state geometries and the excited state data are from the TD-DFT calculations, as described above. Fragments were defined as single chromophores.

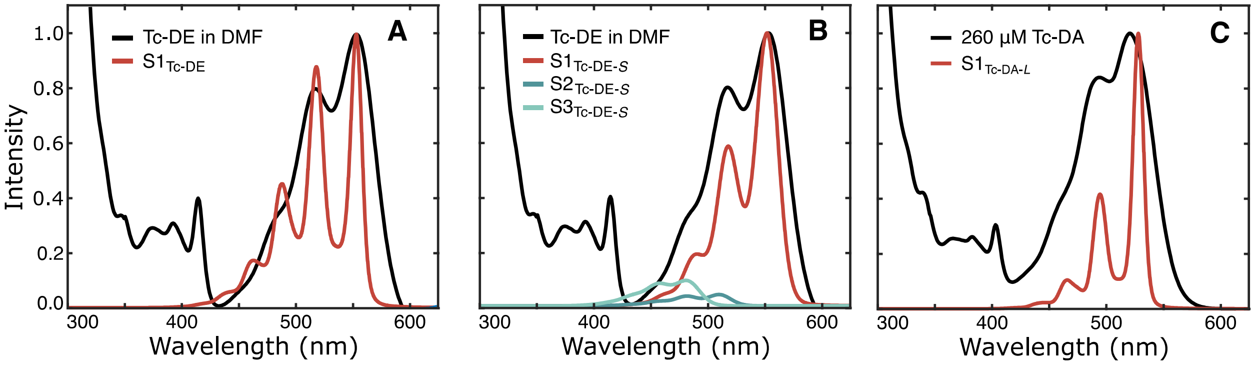

Figure S90. Colored lines show calculated spectra for the **A) Tc-DE** monomer and **B)** stacked dimer and the **C) Tc-DA** linked dimer in DMF. Black lines are observed spectra.

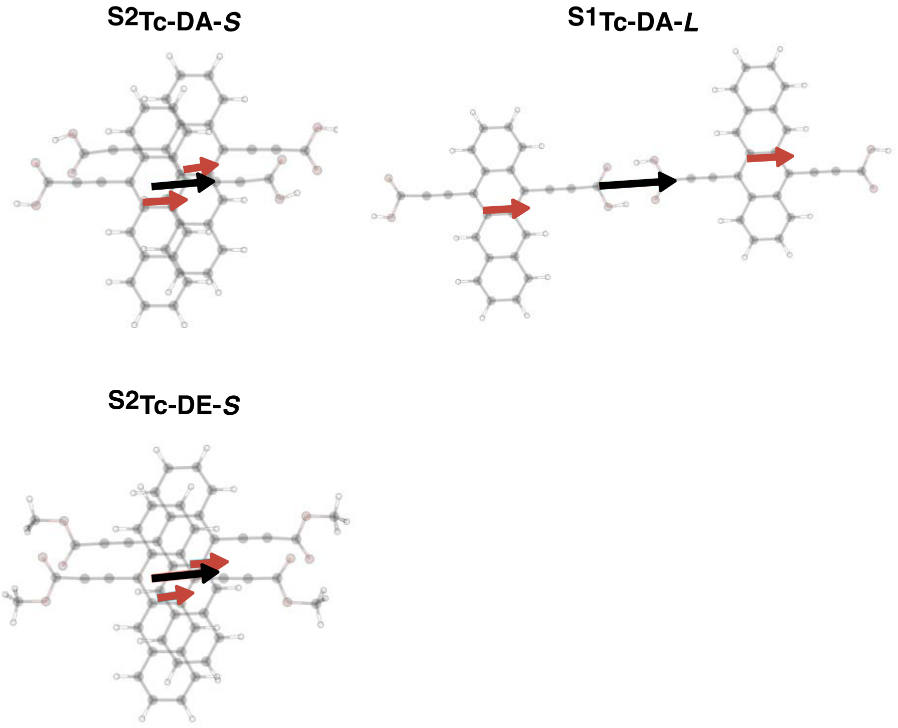

Figure S91. Single-chromophore electron transition dipole moments (red arrows) for the first bright transition couple to give the total transition dipole moments (black arrow) for the indicated **Tc-DA** and **Tc-DE** dimer geometries. The relative orientations of the monomer transition dipole moments for the linked dimer are arranged like a J-aggregate, while those for the stacked dimers resemble an H-aggregate though they are not parallel.

Table. S2 Relaxed triplet T_1 and excited singlet state S_1 energies are given relative to the ground state S_0. The difference in energy between the sum of the ground and first excited singlet state energies (**S_0S_1**) and twice the triplet energy (**T_1T_1**) (ωB97xD/def2-TZVP) is provided in the last column.

	**T_1 (eV)**	**S_1 (eV)**	**S_0S_1-T_1T_1 (eV)**
**Tc gas**	1.28	2.62	0.06
**Tc-DA gas**	0.93	2.19	0.33
**Tc-DA DMF**	0.91	1.95	0.13

Table. S3 Dimer interaction energies taken as the difference between the optimized dimer ground state geometry and the single point energy of each monomer in the dimer at this geometry. A negative value corresponds to stabilization.

	**Interaction Energy (kcal/mol)**	
	DMF	THF
**Tc-DA-S**	-33.19	-32.45
**Tc-DA-L**	-14.47	-15.40
**Tc-DE-S**	-26.79	-27.23

Table. S4 Solvent stabilization energies are computed as the difference between the energy of the system at its optimized ground state geometry (Figure 3) in the gas phase and in the specified solvent using the PCM solvation model.

	**Solvation Stabilization Energy**			
	DMF		THF	
	eV	kcal/mol	eV	kcal/mol
**Tc-DA**	-0.43	-9.98	-0.36	-8.35
**Tc-DA-S**	-0.64	-14.68	-0.53	-12.20
**Tc-DA-L**	-0.64	-14.71	-0.53	-12.23
**Tc-DE**	-0.36	-8.40	-0.30	-6.98
**Tc-DE-S**	-0.58	-13.40	-0.47	-10.94

Table S5 Dominant transitions (DT) for the frontier molecular orbitals with respect to the HOMO (H) and LUMO (L) and their contributions (%), electronic oscillator strength (*f*), TD-DFT computed vertical transition energy (nm), and the percent charge transfer between chromophores in the dimers for the indicated ground to excited state transitions for the **Tc-DA** and **Tc-DE** geometries.

System	State	DT		%	f	nm	CT (%)
Tc-DA	S1	H	L	98	0.291	515	
Tc-DA-*S*	S2	H-1	L	9	0.310	524	0
		H-1	L+1	11			
		H	L	77			
	S3	H-1	L	10	0.031	456	74
		H-1	L+1	12			
		H	L	5			
		H	L+1	70			
	S4	H-1	L	10	0.054	440	77
		H-1	L+1	73			
		H	L	5			
		H	L+1	8			
Tc-DA-*L*	S1	H-1	L	48	0.662	524	0
		H	L+1	50			
Tc-DE	S1	H	L	98	0.274	516	
Tc-DE-*S*	S2	H-1	L+1	10	0.352	515	0
		H	L	88			
	S3	H-1	L	43	0.026	468	48
		H-1	L+1	14			
		H	L+1	40			
	S4	H-1	L	8	0.067	453	49
		H-1	L+1	73			
		H	L	9			
		H	L+1	6			

Optimized geometries

Tc-DA, ωB97xD/def2-TZVP:

C -0.7163000000 -2.0093000000 0.0000000000

C -1.4058000000 -0.7931000000 0.0000000000

C -0.7160000000 0.4479000000 0.0000000000

C 0.7160000000 0.4479000000 0.0000000000

C 1.4058000000 -0.7931000000 0.0000000000

C 0.7163000000 -2.0093000000 0.0000000000

C -1.3947000000 1.6728000000 0.0000000000

C 1.3947000000 1.6728000000 0.0000000000

C 0.7170000000 2.8768000000 0.0000000000

C -0.7170000000 2.8768000000 0.0000000000

C -1.4028000000 4.1322000000 0.0000000000

H -2.4861000000 4.1287000000 0.0000000000

C -0.7136000000 5.2968000000 0.0000000000

C 0.7136000000 5.2968000000 0.0000000000

C 1.4028000000 4.1322000000 0.0000000000

H -2.4775000000 1.6740000000 0.0000000000

H 2.4775000000 1.6740000000 0.0000000000

H -1.2420000000 6.2416000000 0.0000000000

H 1.2420000000 6.2416000000 0.0000000000

H 2.4861000000 4.1287000000 0.0000000000

C -1.3999000000 -3.2638000000 0.0000000000

C -0.7113000000 -4.4293000000 0.0000000000

C 0.7113000000 -4.4293000000 0.0000000000

C 1.3999000000 -3.2638000000 0.0000000000

C 2.8255000000 -0.7953000000 0.0000000000

C 4.0276000000 -0.7943000000 0.0000000000

C -2.8255000000 -0.7953000000 0.0000000000

C -4.0276000000 -0.7943000000 0.0000000000

H 2.4816000000 -3.2645000000 0.0000000000
H 1.2440000000 -5.3714000000 0.0000000000
H -1.2440000000 -5.3714000000 0.0000000000
H -2.4816000000 -3.2645000000 0.0000000000
C 5.4661000000 -0.8717000000 0.0000000000
O 6.0891000000 -1.8978000000 0.0000000000
O 6.0336000000 0.3437000000 0.0000000000
H 6.9893000000 0.2052000000 0.0000000000
C -5.4661000000 -0.8717000000 0.0000000000
O -6.0891000000 -1.8978000000 0.0000000000
O -6.0336000000 0.3437000000 0.0000000000
H -6.9893000000 0.2052000000 0.0000000000

Tc-DA-L, ωB97xD/def2-TZVP:
H 3.8548000000 8.5834000000 0.0000000000
C 6.3923000000 -0.5784000000 0.0000000000
C 9.0776000000 0.2137000000 0.0000000000
C 9.7463000000 2.9844000000 0.0000000000
C 4.2969000000 6.5147000000 0.0000000000
C 8.0668000000 1.2234000000 0.0000000000
C 7.3714000000 3.5805000000 0.0000000000
C 8.3838000000 2.5851000000 0.0000000000
C 7.6761000000 4.9473000000 0.0000000000
C 5.3100000000 5.5047000000 0.0000000000
C 6.6856000000 5.9103000000 0.0000000000
C 4.6289000000 7.8267000000 0.0000000000
C 6.9884000000 7.3085000000 0.0000000000
C 5.9977000000 8.2303000000 0.0000000000
C 5.9977000000 3.1755000000 0.0000000000
C 7.3827000000 -1.5014000000 0.0000000000

C 5.6876000000 1.7898000000 0.0000000000

C 5.0002000000 4.1583000000 0.0000000000

C 6.6929000000 0.8181000000 0.0000000000

C 3.1720000000 1.0528000000 0.0000000000

C 8.7471000000 -1.0991000000 0.0000000000

C 4.3267000000 1.3871000000 0.0000000000

C 10.8992000000 3.3249000000 0.0000000000

H 5.3550000000 -0.8852000000 0.0000000000

H 8.0284000000 7.6115000000 0.0000000000

H 10.1153000000 0.5193000000 0.0000000000

H 7.1384000000 -2.5558000000 0.0000000000

H 9.5246000000 -1.8519000000 0.0000000000

H 3.2589000000 6.2049000000 0.0000000000

H 3.9612000000 3.8537000000 0.0000000000

H 8.7143000000 5.2546000000 0.0000000000

H 6.2372000000 9.2861000000 0.0000000000

O 12.5017000000 4.9831000000 0.0000000000

O 0.9341000000 1.5797000000 0.0000000000

O 1.5251000000 -0.5844000000 0.0000000000

O 13.1883000000 2.8487000000 0.0000000000

C 12.3011000000 3.6571000000 0.0000000000

C 1.8051000000 0.6030000000 0.0000000000

H 0.0070000000 1.2113000000 0.0000000000

H 13.4576000000 5.1208000000 0.0000000000

C -4.2969000000 -6.5147000000 0.0000000000

C -8.0668000000 -1.2234000000 0.0000000000

C -7.3714000000 -3.5805000000 0.0000000000

C -8.3838000000 -2.5851000000 0.0000000000

C -7.6761000000 -4.9473000000 0.0000000000

C -5.3100000000 -5.5047000000 0.0000000000

C -6.6856000000 -5.9103000000 0.0000000000

C -4.6289000000 -7.8267000000 0.0000000000

C -6.9884000000 -7.3085000000 0.0000000000

C -5.9977000000 -8.2303000000 0.0000000000

C -6.3923000000 0.5784000000 0.0000000000

C -5.9977000000 -3.1755000000 0.0000000000

C -7.3827000000 1.5014000000 0.0000000000

C -5.6876000000 -1.7898000000 0.0000000000

C -5.0002000000 -4.1583000000 0.0000000000

C -6.6929000000 -0.8181000000 0.0000000000

C -3.1720000000 -1.0528000000 0.0000000000

C -8.7471000000 1.0991000000 0.0000000000

C -4.3267000000 -1.3871000000 0.0000000000

C -9.0776000000 -0.2137000000 0.0000000000

C -10.8992000000 -3.3249000000 0.0000000000

C -9.7463000000 -2.9844000000 0.0000000000

H -5.3550000000 0.8852000000 0.0000000000

H -8.0284000000 -7.6115000000 0.0000000000

H -10.1153000000 -0.5193000000 0.0000000000

H -7.1384000000 2.5558000000 0.0000000000

H -9.5246000000 1.8519000000 0.0000000000

H -3.2589000000 -6.2049000000 0.0000000000

H -3.9612000000 -3.8537000000 0.0000000000

H -8.7143000000 -5.2546000000 0.0000000000

H -6.2372000000 -9.2861000000 0.0000000000

H -3.8548000000 -8.5834000000 0.0000000000

O -12.5017000000 -4.9831000000 0.0000000000

O -0.9341000000 -1.5797000000 0.0000000000

O -1.5251000000 0.5844000000 0.0000000000

O -13.1883000000 -2.8487000000 0.0000000000

C -12.3011000000 -3.6571000000 0.0000000000
C -1.8051000000 -0.6030000000 0.0000000000
H -0.0070000000 -1.2113000000 0.0000000000
H -13.4576000000 -5.1208000000 0.0000000000

Tc-DA-S, ωB97xD/def2-TZVP:
C -3.4102000000 4.3867000000 -0.3086000000
C -0.0333000000 -0.9226000000 -1.9071000000
C -0.5933000000 1.4227000000 -1.4473000000
C 0.3441000000 0.4174000000 -1.7918000000
C -0.2193000000 2.7667000000 -1.3274000000
C -2.4886000000 3.3692000000 -0.7095000000
C -1.1299000000 3.7412000000 -0.9738000000
C -3.0098000000 5.6742000000 -0.1889000000
C -0.7551000000 5.1149000000 -0.8457000000
C -1.6603000000 6.0463000000 -0.4672000000
C -1.7463000000 -2.6766000000 -1.7470000000
C -1.9541000000 1.0532000000 -1.2048000000
C -0.8195000000 -3.6097000000 -2.0682000000
C -2.3287000000 -0.3116000000 -1.3242000000
C -2.8684000000 2.0470000000 -0.8353000000
C -1.3931000000 -1.2961000000 -1.6585000000
C -4.8405000000 -0.9501000000 -0.9272000000
C 0.5278000000 -3.2374000000 -2.3293000000
C -3.6830000000 -0.6717000000 -1.1041000000
C 0.9085000000 -1.9416000000 -2.2474000000
C 2.8737000000 1.0596000000 -2.0181000000
C 1.7090000000 0.7721000000 -1.9554000000
H -2.7651000000 -2.9684000000 -1.5298000000
H 0.2730000000 5.3887000000 -1.0481000000

H 1.9354000000 -1.6565000000 -2.4329000000

H -1.1016000000 -4.6534000000 -2.1176000000

H 1.2543000000 -4.0001000000 -2.5769000000

H -4.4355000000 4.1036000000 -0.1030000000

H -3.8964000000 1.7708000000 -0.6373000000

H 0.8125000000 3.0426000000 -1.5040000000

H -1.3670000000 7.0839000000 -0.3704000000

H -3.7160000000 6.4364000000 0.1155000000

O 4.6305000000 2.1806000000 -1.0476000000

O -7.0532000000 -0.3327000000 -0.7583000000

O -6.5388000000 -2.4722000000 -0.3852000000

O 5.0948000000 0.5419000000 -2.5147000000

C 4.3067000000 1.2259000000 -1.9193000000

C -6.1940000000 -1.3461000000 -0.6629000000

H -7.9280000000 -0.6795000000 -0.5395000000

H 5.5663000000 2.0501000000 -0.7948000000

C -1.1363000000 2.7549000000 2.3920000000

C 2.3143000000 -2.5400000000 0.8959000000

C 1.7444000000 -0.1951000000 1.3592000000

C 2.6904000000 -1.1989000000 1.0200000000

C 2.0941000000 1.1569000000 1.4451000000

C -0.1906000000 1.7406000000 2.0425000000

C 1.1643000000 2.1245000000 1.7745000000

C -0.7609000000 4.0521000000 2.4694000000

C 1.5126000000 3.5092000000 1.8548000000

C 0.5852000000 4.4349000000 2.1933000000

C 0.5857000000 -4.2886000000 0.9855000000

C 0.3861000000 -0.5759000000 1.6051000000

C 1.5000000000 -5.2189000000 0.6249000000

C 0.0190000000 -1.9404000000 1.4793000000

C -0.5484000000 0.4106000000 1.9452000000

C 0.9535000000 -2.9164000000 1.1298000000

C -2.5136000000 -2.5608000000 1.7307000000

C 2.8510000000 -4.8442000000 0.3846000000

C -1.3445000000 -2.2969000000 1.6509000000

C 3.2446000000 -3.5553000000 0.5180000000

C 5.1984000000 -0.5314000000 0.6456000000

C 4.0449000000 -0.8333000000 0.8090000000

H -0.4451000000 -4.5659000000 1.1602000000

H 2.5342000000 3.7968000000 1.6378000000

H 4.2730000000 -3.2743000000 0.3354000000

H 1.2061000000 -6.2548000000 0.5160000000

H 3.5697000000 -5.6002000000 0.0951000000

H -2.1608000000 2.4608000000 2.5844000000

H -1.5749000000 0.1217000000 2.1328000000

H 3.1121000000 1.4553000000 1.2288000000

H 0.8570000000 5.4813000000 2.2498000000

H -1.4864000000 4.8140000000 2.7226000000

O 7.4252000000 -1.1019000000 0.4916000000

O -4.3101000000 -3.6522000000 0.8001000000

O -4.7134000000 -1.9772000000 2.2443000000

O 6.8706000000 1.0321000000 0.1447000000

C 6.5467000000 -0.1052000000 0.4015000000

C -3.9516000000 -2.6929000000 1.6511000000

H -5.2456000000 -3.5044000000 0.5567000000

H 8.2951000000 -0.7350000000 0.2865000000

Tc-DE, ωB97xD/def2-TZVP:

C 0.0000000000 1.4030000000 4.2800000000

C 0.0000000000 -0.7160000000 -1.8600000000

C 0.0000000000 -0.7160000000 0.5960000000

C 0.0000000000 -1.4070000000 -0.6440000000

C 0.0000000000 -1.3950000000 1.8210000000

C 0.0000000000 0.7170000000 3.0250000000

C 0.0000000000 -0.7170000000 3.0250000000

C 0.0000000000 0.7140000000 5.4450000000

C 0.0000000000 -1.4030000000 4.2800000000

C 0.0000000000 -0.7140000000 5.4450000000

C 0.0000000000 1.3990000000 -3.1150000000

C 0.0000000000 0.7160000000 0.5960000000

C 0.0000000000 0.7110000000 -4.2810000000

C 0.0000000000 1.4070000000 -0.6440000000

C 0.0000000000 1.3950000000 1.8210000000

C 0.0000000000 0.7160000000 -1.8600000000

C 0.0000000000 4.0290000000 -0.6220000000

C 0.0000000000 -0.7110000000 -4.2810000000

C 0.0000000000 2.8270000000 -0.6410000000

C 0.0000000000 -1.3990000000 -3.1150000000

C 0.0000000000 -4.0290000000 -0.6220000000

C 0.0000000000 -2.8270000000 -0.6410000000

H 0.0000000000 2.4810000000 -3.1140000000

H 0.0000000000 -2.4870000000 4.2760000000

H 0.0000000000 -2.4810000000 -3.1140000000

H 0.0000000000 1.2440000000 -5.2230000000

H 0.0000000000 -1.2440000000 -5.2230000000

H 0.0000000000 2.4870000000 4.2760000000

H 0.0000000000 2.4780000000 1.8230000000

H 0.0000000000 -2.4780000000 1.8230000000

H 0.0000000000 -1.2420000000 6.3890000000

H 0.0000000000 1.2420000000 6.3890000000

O 0.0000000000 -6.0430000000 -1.7220000000

O 0.0000000000 6.0430000000 -1.7220000000

O 0.0000000000 6.0650000000 0.5260000000

O 0.0000000000 -6.0650000000 0.5260000000

C 0.0000000000 -5.4680000000 -0.5170000000

C 0.0000000000 5.4680000000 -0.5170000000

C 0.0000000000 -7.4700000000 -1.7190000000

H 0.0000000000 -7.7640000000 -2.7650000000

H 0.8880000000 -7.8510000000 -1.2150000000

H -0.8880000000 -7.8510000000 -1.2150000000

C 0.0000000000 7.4700000000 -1.7190000000

H 0.0000000000 7.7640000000 -2.7650000000

H -0.8880000000 7.8510000000 -1.2150000000

H 0.8880000000 7.8510000000 -1.2150000000

Tc-DE-S, ωB97xD/def2-TZVP:

C -2.1837000000 4.9774000000 -0.4956000000

C 0.2867000000 -0.8492000000 -1.9466000000

C 0.1429000000 1.5402000000 -1.4017000000

C 0.9040000000 0.3774000000 -1.6867000000

C 0.7480000000 2.7778000000 -1.1493000000

C -1.4276000000 3.8055000000 -0.8130000000

C 0.0015000000 3.9037000000 -0.8654000000

C -1.5642000000 6.1565000000 -0.2559000000

C 0.6118000000 5.1713000000 -0.6101000000

C -0.1418000000 6.2563000000 -0.3176000000

C -1.7510000000 -2.2070000000 -2.1729000000

C -1.2843000000 1.4454000000 -1.3617000000

C -0.9954000000 -3.2963000000 -2.4468000000

C -1.9005000000 0.1937000000 -1.6188000000

C -2.0332000000 2.5898000000 -1.0604000000

C -1.1418000000 -0.9421000000 -1.9138000000

C -4.5125000000 0.0381000000 -1.4781000000

C 0.4230000000 -3.2053000000 -2.4785000000

C -3.3146000000 0.0987000000 -1.5539000000

C 1.0419000000 -2.0265000000 -2.2345000000

C 3.5188000000 0.5587000000 -1.6664000000

C 2.3207000000 0.4667000000 -1.6874000000

H -2.8298000000 -2.2784000000 -2.1371000000

H 1.6921000000 5.2391000000 -0.6482000000

H 2.1217000000 -1.9588000000 -2.2445000000

H -1.4711000000 -4.2502000000 -2.6339000000

H 1.0096000000 -4.0905000000 -2.6857000000

H -3.2634000000 4.8988000000 -0.4521000000

H -3.1125000000 2.5153000000 -1.0186000000

H 1.8280000000 2.8488000000 -1.1713000000

H 0.3302000000 7.2114000000 -0.1251000000

H -2.1461000000 7.0379000000 -0.0176000000

O 5.3762000000 1.8155000000 -1.2132000000

O -6.5007000000 0.9989000000 -0.8722000000

O -6.5206000000 -1.1559000000 -1.5097000000

O 5.6823000000 -0.2876000000 -1.9465000000

C 4.9599000000 0.6230000000 -1.6333000000

C -5.9337000000 -0.1246000000 -1.3024000000

C -0.9259000000 3.0486000000 2.4353000000

C 1.2930000000 -2.8867000000 1.0195000000

C 1.2506000000 -0.4922000000 1.5566000000

C 1.9610000000 -1.6815000000 1.2497000000

C 1.9088000000 0.7202000000 1.7938000000

C -0.2209000000 1.8398000000 2.1400000000

C 1.2114000000 1.8755000000 2.0859000000

C -0.2565000000 4.2012000000 2.6653000000

C 1.8766000000 3.1176000000 2.3310000000

C 1.1687000000 4.2356000000 2.6129000000

C -0.7956000000 -4.1711000000 0.8483000000

C -0.1796000000 -0.5293000000 1.6120000000

C -0.0889000000 -5.2896000000 0.5621000000

C -0.8472000000 -1.7575000000 1.3693000000

C -0.8790000000 0.6497000000 1.8993000000

C -0.1360000000 -2.9269000000 1.0859000000

C -3.4669000000 -1.8360000000 1.4230000000

C 1.3308000000 -5.2471000000 0.4873000000

C -2.2659000000 -1.7958000000 1.4042000000

C 1.9970000000 -4.0894000000 0.7083000000

C 4.5736000000 -1.5820000000 1.0675000000

C 3.3763000000 -1.6342000000 1.1580000000

H -1.8758000000 -4.1991000000 0.8964000000

H 2.9587000000 3.1405000000 2.2843000000

H 3.0766000000 -4.0549000000 0.6500000000

H -0.6045000000 -6.2248000000 0.3854000000

H 1.8795000000 -6.1505000000 0.2534000000

H -2.0083000000 3.0210000000 2.4606000000

H -1.9613000000 0.6254000000 1.9288000000

H 2.9897000000 0.7513000000 1.7408000000

H 1.6801000000 5.1724000000 2.7934000000

H -0.8003000000 5.1127000000 2.8761000000

O 6.5560000000 -2.4785000000 0.3475000000

O -5.3825000000 -3.0631000000 1.1843000000

O -5.5878000000 -0.8754000000 1.6596000000

O 6.5856000000 -0.4081000000 1.2203000000

C 5.9951000000 -1.4087000000 0.9017000000

C -4.9101000000 -1.8461000000 1.4400000000

C 6.7935000000 1.9774000000 -1.1122000000

H 7.2642000000 1.8038000000 -2.0798000000

H 6.9446000000 3.0054000000 -0.7952000000

H 7.1950000000 1.2853000000 -0.3733000000

C 7.9358000000 -2.3425000000 0.0102000000

H 8.2282000000 -3.2993000000 -0.4128000000

H 8.0579000000 -1.5479000000 -0.7256000000

H 8.5288000000 -2.1176000000 0.8963000000

C -6.8067000000 -3.1803000000 1.1406000000

H -7.0044000000 -4.2344000000 0.9680000000

H -7.2427000000 -2.8602000000 2.0869000000

H -7.2037000000 -2.5781000000 0.3245000000

C -7.8806000000 0.8954000000 -0.5255000000

H -8.1794000000 1.8927000000 -0.2155000000

H -8.4700000000 0.5674000000 -1.3813000000

H -8.0016000000 0.1899000000 0.2965000000

References

(1) Rurack, K.; Spieles, M. Fluorescence Quantum Yields of a Series of Red and Near-Infrared Dyes Emitting at 600−1000 Nm. Anal. Chem. **2011**, 83 (4), 1232–1242. https://doi.org/10.1021/ac101329h.

(2) Spano, F. C. The Spectral Signatures of Frenkel Polarons in H- and J-Aggregates. Acc. Chem. Res. **2010**, 43 (3), 429–439. https://doi.org/10.1021/ar900233v.

(3) Sandros, K.; Haglid, F.; Ryhage, R.; Stevens, R. Transfer of Triplet State Energy in Fluid Solutions. III. Reversible Energy Transfer. Acta Chem. Scand. **1964**, 18, 2355–2374.

(4) Singh, A.; Johnson, L. W. Phosphorescence Spectra and Triplet State Lifetimes of Palladium Octaethylporphyrin, Palladium Octaethylchlorin and Palladium 2, 3-Dimethyloctaethylisobacteriochlorin at 77 K. Spectrochim. Acta. A. Mol. Biomol. Spectrosc. **2003**, 59 (5), 905–908.

(5) Gray, V.; Dzebo, D.; Lundin, A.; Alborzpour, J.; Abrahamsson, M.; Albinsson, B.; Moth-Poulsen, K. Photophysical Characterization of the 9, 10-Disubstituted Anthracene Chromophore and Its Applications in Triplet–Triplet Annihilation Photon Upconversion. J. Mater. Chem. C **2015**, 3 (42), 11111–11121.

(6) Hoseinkhani, S.; Tubino, R.; Meinardi, F.; Monguzzi, A. Achieving the Photon Up-Conversion Thermodynamic Yield Upper Limit by Sensitized Triplet–Triplet Annihilation. Phys. Chem. Chem. Phys. **2015**, 17 (6), 4020–4024.

(7) O'shea, R.; Gao, C.; Bradley, S.; Owyong, T. C.; Wu, N.; White, J. M.; Ghiggino, K. P.; Wong, W. W. H. Limitations of Conjugated Polymers as Emitters in Triplet–Triplet Annihilation Upconversion. Mater Adv **2021**, 2 (23), 7751–7758. https://doi.org/10.1039/D1MA00684C.

(8) Vincett, P.; Voigt, E.; Rieckhoff, K. Phosphorescence and Fluorescence of Phthalocyanines. J. Chem. Phys. **1971**, 55 (8), 4131–4140.

(9) Rihter, B. D.; Kenney, M. E.; Ford, W. E.; Rodgers, M. A. Synthesis and Photoproperties of Diamagnetic Octabutoxyphthalocyanines with Deep Red Optical Absorbance. J. Am. Chem. Soc. **1990**, 112 (22), 8064–8070.

(10) Ford, W.; Rihter, B.; Kenney, M.; Rodgers, M. Photoproperties of Alkoxy-Substituted Phthalocyanines with Deep-Red Optical Absorbance. Photochem. Photobiol. **1989**, 50 (3), 277–282.

(11) Frisch, M. J.; Trucks, G. W.; Schlegel, H. B.; Scuseria, G. E.; Robb, M. A.; Cheeseman, J. R.; Scalmani, G.; Barone, V.; Petersson, G. A.; Nakatsuji, H.; Li, X.; Caricato, M.; Marenich, A. V.; Bloino, J.; Janesko, B. G.; Gomperts, R.; Mennucci, B.; Hratchian, H. P.; Ortiz, J. V.; Izmaylov, A. F.; Sonnenberg, J. L.; Williams; Ding, F.; Lipparini, F.; Egidi, F.; Goings, J.; Peng, B.; Petrone, A.; Henderson, T.; Ranasinghe, D.; Zakrzewski, V. G.; Gao, J.; Rega, N.; Zheng, G.; Liang, W.; Hada, M.; Ehara, M.; Toyota, K.; Fukuda, R.; Hasegawa, J.; Ishida, M.; Nakajima, T.; Honda, Y.; Kitao, O.; Nakai, H.; Vreven, T.; Throssell, K.; Montgomery Jr., J. A.; Peralta, J. E.; Ogliaro, F.; Bearpark, M. J.; Heyd, J. J.; Brothers, E. N.; Kudin, K. N.; Staroverov, V. N.; Keith, T. A.; Kobayashi, R.; Normand, J.; Raghavachari, K.; Rendell, A. P.; Burant, J. C.; Iyengar, S. S.; Tomasi, J.; Cossi, M.; Millam, J. M.; Klene, M.; Adamo, C.; Cammi, R.; Ochterski, J. W.; Martin, R. L.; Morokuma, K.; Farkas, O.; Foresman, J. B.; Fox, D. J. Gaussian 16 Rev. C.01, 2016.

(12) C. A. Valente, D.; Do Casal, M. T.; Barbatti, M.; Niehaus, T. A.; Aquino, A. J. A.; Lischka, H.; Cardozo, T. M. Excitonic and Charge Transfer Interactions in Tetracene Stacked and T-Shaped Dimers. J. Chem. Phys. **2021**, 154 (4), 044306. https://doi.org/10.1063/5.0033272.

(13) Engels, B.; Engel, V. The Dimer-Approach to Characterize Opto-Electronic Properties of and Exciton Trapping and Diffusion in Organic Semiconductor Aggregates and Crystals. Phys. Chem. Chem. Phys. **2017**, 19 (20), 12604–12619. https://doi.org/10.1039/C7CP01599B.

(14) Papadopoulos, I.; Álvaro-Martins, M. J.; Molina, D.; McCosker, P. M.; Keller, P. A.; Clark, T.; Sastre-Santos, Á.; Guldi, D. M. Solvent-Dependent Singlet Fission in Diketopyrrolopyrrole Dimers: A Mediating Charge Transfer versus a Trapping Symmetry-Breaking Charge Separation. Adv. Energy Mater. **2020**, 10 (43), 2001496. https://doi.org/10.1002/aenm.202001496.

(15) Cerezo, J.; Santoro, F. FCclasses3 : Vibrationally-resolved Spectra Simulated at the Edge of the Harmonic Approximation. J. Comput. Chem. **2023**, 44 (4), 626–643. https://doi.org/10.1002/jcc.27027.

(16) Lu, T.; Chen, F. Multiwfn: A Multifunctional Wavefunction Analyzer. J. Comput. Chem. **2012**, 33 (5), 580–592. https://doi.org/10.1002/jcc.22885.
